# Gene functions determine stochastic or adaptive futile transcription in cancers through deregulated start site deployments

**DOI:** 10.3389/fbinf.2026.1883461

**Published:** 2026-10-14

**Authors:** Aditi Lakshmi Satish, Praveen Kumar, Ishani Morbia, Umashankar Singh

**Affiliations:** Department of Biological Sciences and Engineering, HoMeCell Lab, Indian Institute of Technology Gandhinagar, Gandhinagar, India

**Keywords:** alternative TSS, cancer, gene function, stochastic, transcriptional cost, TSS distance

## Abstract

Cancers display near-universal hallmarks, including energy addiction, aerobic glycolysis, and biosynthesis. In addition to glucose (and glutamine) addiction, cancers display futile transcription. Discretely measurable futile transcription occurs between transcription start sites (TSSs) and start codons of protein-coding genes. Multiple TSSs for each gene offer various combinations of futile transcription with no effect on the encoded proteins. The relationship between proximal and distal TSS deployment, futile transcription, and energy addiction of cancers remains unclear. By analyzing CAGE and PRO-cap data, we show that TSS deployment dysregulation in cancers increases the energy cost coefficients of cancer transcriptomes. We define the bases of the altered energy cost coefficients of cancer transcriptomes by comparing TSS deployment frequencies, associated entropies, and futile transcription distances across heterogeneous pools of cancers and non-cancers. We show that TSS deployment entropies differ between cancers and non-cancers. It leads to an overall higher distal TSS deployment in cancers but selectively favors an energy-economical proximal TSS deployment for cancer hallmark genes involved in cell proliferation and biosynthesis. We show that the frequency of TSS deployment is linked differently to futile transcription distance and gene function in cancers and non-cancers. This work lays out a theoretical framework describing the stochasticity of TSS deployment in the context of cancerogenesis and the energetics of transcription. It also suggests that the current human TSS landscape has evolved to minimize futile transcription, an outcome favored by stochastic TSS deployment. Under normal conditions of energy metabolism, it forms a threshold that incipient cancer cells breach.

## 1 Introduction

Cellular transformation is at the molecular core of cancerogenesis. A concerted deregulation of multiple checkpoints over a long period leads to the manifestation of cancer (Hanahan and Weinberg, 2011). The outcome of multiple cell-transforming perturbations depends on various factors, such as cellular epigenotypes, genotypes, stemness, and macroenvironmental and microenvironmental influences. Remarkably, over and above this heterogeneity, the path to cellular transformation and its eventual manifestation as a disease is common across cancers to the extent that near-universal hallmarks for cancer have been prescribed (Berardi and Fantin, 2011). These hallmarks of cancer include the loss of tumor-suppressor checkpoints, oncogenic drive, immortalization, and escape from programmed cell death as core features, for which distinct sets of genes have been identified (Berardi and Fantin, 2011; Auslander et al., 2019; Hanahan and Weinberg, 2000; Swanton et al., 2024). It can be argued that the universal nature of cancer hallmarks is due to the primordial and highly conserved nature of molecular pathways that guide some of these fundamental normal cellular processes. Some of the first set of universal hallmarks of cancer to be described was perturbations in well-conserved processes, including cell proliferation (cell cycle), contact inhibition (cell migration and cell–cell communication), and the Warburg effect (energy metabolism and biosynthesis). These features of cancer, which deviate from nature’s law of “optimization and least action,” can be justified only if they present evolutionary advantages to an incipient cancer cell, leading to their selection during tumorigenesis. With the advent of modern techniques, deeper insights into cancerogenesis have been obtained (Swanton et al., 2024; Bradner et al., 2017). We now understand the molecular nature of genetic and epigenetic changes, their heterogeneity in a tumor cell mass, and their influence on gene expression (Swanton et al., 2024; Bradner et al., 2017; Dawson and Kouzarides, 2012; Jones et al., 2016). Altered gene expression is the ultimate bottleneck through which mutations and epimutations alter cellular fates, contributing to the manifestation of hallmark features and the multistep process of tumor evolution.

Altered gene expression is one of the most well-studied phenomena in cancers. A lingering question in such studies has been the choice of appropriate controls for comparative gene expression analyses. Despite such limitations, several reproducible and predictable patterns of gene expression deregulation have been discovered in cancers that transcend the heterogeneity of cancer and normal sample types (Gao and Teschendorff, 2017; Rapin et al., 2014; Kagohara et al., 2018; Su et al., 2021; Yang et al., 2015; Tahiri et al., 2014; Jha et al., 2022). The molecular outcomes of common oncogenic, tumor-suppressor, and restriction checkpoint mutations/epimutations are often traceable to downstream transcription regulators, transcription factors, and associated gene expression. A common feature of gene expression deregulation in cancers is the transcriptional addiction of cancer cells (Bradner et al., 2017; Quek et al., 2022; Wise and Thompson, 2010), evidenced by glutamine addiction (Cluntun et al., 2017), high RNA polymerase II activity (Bradner et al., 2017; Lee and Young, 2013), and biosynthetic bias in energy metabolism (M et al., 2023). Additional layers of transcription deregulation involve posttranscriptional gene silencing in the form of altered lengths of untranslated regions (UTRs) and microRNAs (Mularoni et al., 2016; Dobosz et al., 2022; Kim and Croce, 2023; Adashek et al., 2020; Weinberg and Morris, 2016). As such, transcription, the first step in gene expression, seems to integrate metabolism and energy addiction, mutations, epimutations, and cellular environments.

Resistance to gene expression deregulation seems to be the key barrier to be breached for any event to stably exert its phenotypic effects. Regulation of transcription is a primary mechanism to control RNA synthesis (Jones et al., 2016; Yang et al., 2015; Frankish et al., 2023; You and Jones, 2012). The identity of what constitutes a promoter and levies regulation on genes is fundamentally determined by the transcription start site (TSS). Given the vastness of our genome as compared with the small number of protein-coding genes, there are immense possibilities for multiple TSSs for each gene. It is established that there are multiple TSSs for most genes. In some cases, this leads to alterations in splicing and reading frames (Wang et al., 2016; Hirabayashi et al., 2019; Arner et al., 2015; Hashimoto et al., 2015; FANTOM Consortium and the RIKEN PMI and CLST (DGT) et al., 2014; Shiraki et al., 2003; Kanamori-Katayama et al., 2011; Ni et al., 2010). However, for most protein-coding genes, the choice of TSSs does not impact the final protein product they code for. However, it is not fully understood how TSSs are selected or deployed. While different sequence features are expected to bestow different transcription starting capacities on different TSSs, it remains unknown how exactly TSS selection is made during the process of transcription (Zhao et al., 2024; Demircioğlu et al., 2019; Nepal and Andersen, 2023; Huang et al., 2021). The current understanding is that TSS deployment is largely stochastic, as supported by experimental and computational studies (FANTOM Consortium and the RIKEN PMI and CLST (DGT) et al., 2014; de Klerk and ’t Hoen, 2015; Deng et al., 2014; Raj et al., 2006; Raj and van Oudenaarden, 2008; Kepler and Elston, 2001; Lipniacki et al., 2006; Friedrich et al., 2019; Cao et al., 2020).

However, despite “no difference” in the encoded protein, the deployment of proximal or distal TSSs entails very different energy costs. The understanding that such a crucial cell-fate-determining, energy-intensive process is outsourced plainly to stochasticity pits itself against the evolutionary selection of distal TSSs. Moreover, the incentive underlying the choice of one TSS over the other for the same gene and encoded protein remains unaddressed, especially when a distal TSS is preferred over a proximal one. The functional implications of the overall stochasticity of transcription can be better understood by separately addressing its two major dimensions: the frequency of transcription and TSS selection. The latter may not be significant if TSSs are clustered tightly such that local DNA sequence and chromatin neighborhoods do not distinguish various TSSs in a cluster, and stochasticity prevails in selecting TSSs in a cluster with no preference (Karlsson et al., 2017). However, when alternative TSSs are located far apart, their different chromatin neighborhoods add a regulatory layer to their otherwise stochastic deployment (Preikšaitienė and Kučinskas, 2016; Gonzalez-Sandoval and Gasser, 2016; Harris et al., 2023; Pollex et al., 2024). It is implicit that transcription from distal TSSs would be resource intensive, which shall be justified by the regulatory advantages its deployment provides to the cell.

Long-distance upstream transcription beyond an unavoidable length (minimal length of DNA required for RNA polymerase II positioning and subsequent firing) is futile unless the regulatory benefits offset the overall resource and energy cost (hitherto referred to as “energy cost”). Each base transcribed is an avoidable drain on cellular energy resources. The net energy cost of upstream transcription can be assumed to be a function of upstream region length (distance between TSS and start codon) and transcriptional activity (frequency of deployment of the TSS). The energy costs of futile transcription must pose a challenge to the usability of long-distance upstream TSSs, which gets compounded by alternative translation initiation possibilities due to long 5′ UTRs (Marziali et al., 2019; Makalowska et al., 2005; Renz et al., 2020; McGillivray et al., 2018). In addition, the regulatory features of the TSS-start codon region are likely to suffer a higher mutational load proportional to their lengths (Mularoni et al., 2016; Kutchko et al., 2015; Cottre et al., 2018). This mutational load disadvantage is only likely to resist dependence on long-distance upstream TSSs over long periods. In contrast, the energy costs of transcription from long-distance TSSs are likely to act as an immediate impediment. Thus, the energy costs of transcription would act as a barrier to the usage of long-distance TSSs in cells that are normally sensitive to the fundamental premise of energy conservation.

We use this energy conservation argument to hypothesize that regulatory mechanisms in non-cancer cells, regardless of their cell types, developmental lineages, and other variabilities, resist long-distance futile transcription. We analyze curated CAGE (FANTOM Consortium and the RIKEN PMI and CLST (DGT) et al., 2014; Noguchi et al., 2017; Carninci et al., 2005) and PRO-cap (NCBI GEO and ENCODE) data (ENCODE Project Consortium et al., 2020) to test this hypothesis and report that the disruption of transcriptional energy cost minimization is a feature of cancers in general. Genes linked with cancer hallmarks of enhanced biosynthesis and cell proliferation undergo a high rate of deployment from very proximal TSSs and increased transcription from the highly distal TSSs, thereby increasing total transcriptional energy cost. We argue that transcription cost minimization is a property of non-cancers lost in cancers near-universally akin to being a cancer hallmark. Transcriptional energy cost optimization appears to be a force behind the evolution of TSSs and their deployment patterns, and it is constantly under feedback from biosynthetic and proliferative needs of cells.

## 2 Methods

### 2.1 Estimating TSS distance ( d ) and its derivatives

For each annotated TSS of a protein-coding gene in the CAGE profiles (publicly available FANTOM5 datasets; Supplementary Material, Sections 1, 2), the corresponding first start codon at the 5′ end was identified using GENCODE v45 keeping in consideration the strand identity (20,109 start codons). Genes annotated as antisense and non-coding and those from the pseudo autosomal regions were excluded from the analysis (n = 20,072 start codons). These start codons were then mapped to the corresponding TSSs by their gene identities using bedtools, which gave a total of 57,461 TSS-start codon combinations from 16,302 genes. The distance between the TSS and the start codon was calculated independently for each strand. The distance for forward-strand TSSs was measured as the absolute difference of the start coordinate of the TSS from the start codon start site (n = 29,136 TSSs) and on the reverse strand as the absolute difference of the start codon end coordinate from the TSS end coordinate (n = 28,325 TSSs) wherein the coordinates were all from the plus strand. Details are presented in supplementary data (Supplementary Table S1).

For 5′ UTR benchmarking, we used the hg38 *knownGene* GTF file from UCSC. The GTF file was filtered to get only the 5′ UTR coordinates (n = 185,974). The length of the region on the genome (including introns) was used as the distance of the 5′ UTR.

From the d , derivatives of d were generated as mentioned below with details in supplementary data (Supplementary Tables S2 and S3) and Supplementary Material (Section 3).

#### 2.1.1 Incremental d

$$d_{i}=\frac{d_{u}-d_{p}}{d_{p}}.$$

#### 2.1.2 Fractional d

$$d_{f}=\frac{d_{t}}{d_{\max}}.$$

#### 2.1.3 Gene-level distance HMd

$$HMd_{g}=\frac{\left|\tau \right|}{\sum_{t}^{\tau }\frac{1}{d_{t}}}.$$

### 2.2 Normalizing and scaling of CAGE scores

CAGE samples were curated and classified as cancer (*C*) or non-cancer (*N*) (Supplementary Tables S4 and S5; 1,255 *N* and 476 *C*). The raw CAGE matrices were either normalized or scaled, depending on the analyses. Normalization of a TSS in a sample was done as the share of that score in the entire promoterome independently for *N* and *C*:

$$score_{t,s}=\frac{raw\;score_{t,s}}{\sum_{t=1}^{55048}\sum_{s=1}^{S}raw\;score_{t,s}},$$

where $score_{t,s}$ is the normalized score of TSS t in sample s , $raw\;score_{t,s}$ is the raw score of the TSS t in sample s , t is the TSS, s is a sample in the sample set S , and S is the cancer or non-cancer sample set.

Scaling, however, was performed commonly for both *N* and *C*. A scaling factor was estimated for each sample such that the sum of scores for each sample would be equal after scaling:

$$\gamma_{t,s}=raw\;score_{t,s}*f_{s},$$

where $\gamma_{t,s}$ is the scaled score of TSS t in sample s , $raw\;score_{t,s}$ is the raw score of TSS t in sample s , $f_{s}$ is the scaling factor for sample s , t is the TSS, s is the sample in the sample set S , and S is the cancer or non-cancer sample set.

Note: All parameters derived from the raw score, normalized score, or scaled scores are dependent on the sample set of the parameters. All the parameters described below would therefore contain a value in *N* and one in *C*. The sample ( s ) or sample set ( S ) identities, although not mentioned for brevity, are implied unless mentioned otherwise. The metadata-blind nomenclature of samples (Supplementary Table S4) is traceable to actual FANTOM annotations and metadata (Supplementary Table S5).

### 2.3 Score-derived parameters

Multiple parameters were generated using TSS activity scores and/or d as tabulated (Table 1). Details of these parameters and associated equations are mentioned in Supplementary Tables S6–S12 and Supplementary Material (Sections 4–11).

**TABLE 1:** The table provides a list of major parameters used throughout the manuscript, mathematical operations used to derive them, and references to supplementary data for more details.

Parameter	Formula	Equation number	Supplementary table and Supplementary Materials section
Transcript count index	$TCI_{t}=\sum_{s}^{S}score_{t,s}*10^{9}$	*VI*	Supplementary Table S6 and Section 5
Energy cost coefficient of transcription of a TSS	$E_{t}={\left(TCI*d\right)}_{t}$	*VII*	Supplementary Table S6 and Section 6.1
Energy cost coefficient of transcription of a gene	$E_{g}=\sum_{t}^{\tau }{\left(TCI*d\right)}_{t}$	*VIII*	Supplementary Table S7 and Section 6.2
Base count index	$BCI_{t}=\sum_{s}^{S}{\left(score_{s}*d\right)}_{t}*10^{9}$	*IX*	Supplementary Table S8 and Section 7
Effective distance of a gene	$vd_{g}=\sum_{t}^{\tau }\frac{{\left(TCI*d\right)}_{t}}{\sum_{t}^{\tau }TCI_{t}}$	*X*	Supplementary Table S9 and Section 8.1
Gene-space entropy	$gH_{g}=-\sum_{t}^{\tau }P_{g}\left(t\right)*\log_{\left|\tau \right|}P_{g}\left(t\right)$	*XIII*	Supplementary Table S10 and Section 9
Sample-space entropy	$\mathrm{sH}=\frac{\sum_{i=1}^{10}\left(‐\sum_{s=1}^{100}P_{s}\left(t\right)*\log_{2}P_{s}\left(t\right)\right)}{10}$	*XV*	Supplementary Table S11 and Section 10
Compound entropy	$cH=-\sum_{t}^{\tau }P_{cH}\left(t\right)*\log_{\tau }P_{cH}\left(t\right)$	*XVII*	Supplementary Table S12 and Section 11

### 2.4 TSS equalization simulation

This simulation was done to equalize TSS activity ( TCI ) per gene iteratively for *N* and *C*. For each gene, the TSSs were first sorted in ascending order of their d . Iteratively, consecutive TSS activities were replaced by their means, thus equalizing their corresponding TCI s independently in *N* and *C*. The total number of iterations of TCI equalizations was governed by the maximum TSS count for a gene. In our data, it was the *SORBS2* gene with 39 TSSs; thus, 38 iterations of equalizations (plus one non-equalizing zeroth iteration) were done to exhaust all TSSs. Those genes with a fewer number of TSSs exhaust them much before this iteration and do not participate further. After every iteration, the gene-space entropy and the gene-level E were calculated and compared between *N* and *C* through a gene-paired Wilcoxon test. The simulation was done twice: first, in the ascending order of d per gene (most proximal TSS to the most distal TSS), and second, in the descending order of d per gene (most distal TSS to the most proximal TSS).

### 2.5 Promoterome entropy maximization simulation

A simulated promoterome was generated with an equal number of random samples from *N* and *C* and completely entropized samples. The promoterome starts as a matrix of random samples of *N* and *C* scaled scores only. With every iteration, one sample each of *N* and *C* was replaced by completely entropized samples. Over 50 iterations, the simulated promoterome shifted from completely *N* and *C* to completely entropized. A completely entropized sample was generated using the mean of scaled scores of any sample. All TSSs of this sample thus have the same score. In every iteration, the simulated promoterome was used to calculate the E , gH , sH , and cH . Since the sample selection involved randomization, 10 subiterations were performed for each iteration, and the mean of each of the parameters was calculated as a single value per iteration. The TSSs were then segregated by the d -bins: 0–1, 1–5, 5–10, 10–50, and >50 kb. For each d -bin, an exponential curve was fit for the $\sum_{t=1}^{33232}{sH}_{t,i}$ versus $\sum_{t=1}^{33232}E_{t,i}$ data for every iteration. The sH – $E_{t}$ relationship was best described by the exponential growth function with a rate constant parameter ( $y=y_{0}+Ae^{R_{0}x}$ ) for all bins except for the first, where the correlation could not be interpreted (Supplementary Table S13). The actual mean sH for each d -bin obtained from the *N* and *C* data was used to interpolate the corresponding $E_{t}$ . The curve fitting and interpolation were done using Origin 2017.

### 2.6 Promoterome substitution simulation

A simulated promoterome was constructed with 100 randomly selected cancer samples. In every iteration, the cancer samples were replaced with an iteration count number of non-cancer samples. Each iteration involved 10 subiterations, where the sample-space entropy and energy cost coefficient were recorded per TSS, and the mean of the subiterations was used as the representative value per iteration. The TSSs were split by the distance binned by 0.1 or 1 kb and plotted to compare sH and $E_{t}$ for every iteration.

### 2.7 Functional category enrichment

All functional category enrichment analyses were performed on GOrilla. The terminal quartiles (Q1 and Q4) of the C/N ratios for each of the parameters ( vd,gH,sH , and vd/cH ) sorted were used to produce two gene sets. Gene Ontology (GO) biological process (BP) and molecular function (MF) term enrichment was performed on these two sets in two different directions of comparison using GOrilla (Lynch and Marinov, 2015; Eden et al., 2007; Eden et al., 2009) at default settings for analyses of non-ranked lists. Both gene sets were analyzed as paired gene lists where one gene set was used as a foreground against the other as the background for the enrichment analysis (foreground/background: Q1/Q4 and Q4/Q1). GO terms that satisfied the *p*-value criteria were manually assigned a broader functional category (*Biosynthesis and metabolism*, *Cell proliferation*, *DNA and chromatin organization*, *Signaling and homeostasis*, *Development and differentiation*, and *Others*). The results of GO BP term enrichments using various parameters are presented in supplementary tables as follows: vd (Supplementary Table S14), gH (Supplementary Table S15), sH (Supplementary Table S16), and vd/cH (Supplementary Table S17). Some root-level GO terms could not be assigned one blanket term unequivocally and were also classified as *Others*.

### 2.8 Principal component analysis

We calculated a sample-level effective d for each of the 1,731 CAGE profiles and 15,861 genes (as described in Supplementary Material 8.2; TCI was replaced by scaled scores for the calculation). Principal component analysis (PCA) was performed on the same data using the R package *PCAtools*, and the first three principal components were plotted. The metadata for the PCA annotations were curated manually (see Methods, 2.1) and are available in Supplementary Tables S4 and S5. The variances associated with the principal components were compared through Levene’s test from the *cars* package.

### 2.9 Validation using PRO-cap data

Publicly available PRO-cap data for humans were downloaded from NCBI GEO as hg38-aligned bigWig files along with the corresponding metadata including GSM accessions, ENCODE accessions, and biological sample details (Supplementary Table S18). The data were curated manually to include cancers and non-cancers and exclude embryonic stem cell-like samples. The curation resulted in 305 *N* and 110 *C* (see Supplementary Material, Section 2). Since PRO-cap peaks could not be unequivocally assigned to genes, we used the PRO-cap signal mapping to CAGE peak coordinates (see Supplementary Material, Section 12). No *de novo* peak calling was performed for PRO-cap data. A total of 52,904 distance-mapped TSSs were reported commonly between the PRO-cap *N* and *C* samples. Out of these, 52,185 TSSs were common to the 55,048 commonly represented TSSs in CAGE *N* and *C* sample sets. All score-derived parameters were calculated, replacing the CAGE data scores with PRO-cap scores. Details of PRO-cap analyses are presented in supplementary data as follows: ENCODE and curated PRO-cap annotations (Supplementary Table S18), normalized PRO-cap scores for 52,185 TSSs (Supplementary Table S19), summary of the parameter comparisons across experiments and TSS set (Supplementary Table S20), and PRO-cap vd/cH (Supplementary Table S21). GO BP term enrichments for terminal quartile comparisons using PRO-cap vd/cH are mentioned in Supplementary Table S22.

### 2.10 Cost description

TSS- and gene-level costs of transcription were measured for CAGE and PRO-cap data using approximations described earlier (Lynch and Marinov, 2015):

$${COST}_{RNA,e}≃N_{r}\left(46\cdot L_{r,mat}+2.17\cdot \delta_{r}tL_{r,pre}\right).$$

The transcriptional cost of the aggregated promoterome ( CAP ) of *N* ( $CAP_{N}$ ) or *C* ( $CAP_{C}$ ) was calculated using the following formula:

$${CAP}_{s}=\sum_{s}^{S}{score}_{s}\left(46\cdot d_{t,exon}+2.17\cdot \kappa_{s}d_{t}\right),$$

where score is $N_{r}$ equivalent (number of transcripts), $d_{t}$ is the transcribed region length in terms of the ribonucleotide base count (exonic plus intronic), and $d_{t,exon}$ is the transcribed region length in terms of the ribonucleotide base count (exonic only).

Thus, the originally described length parameter *L*_*r*_ has been restricted to *d*. Accordingly, introns and splicing events have been calculated only for *d*. We have allowed each intra-*d* intron to either remain unspliced or get spliced once independently. No combinatorial splicings have been accounted for (Supplementary Table S23).

κ combines all the components due to which transcript counts and the number of phosphate bonds hydrolyzed for RNA synthesis mismatch. Primarily, these include the RNA turnover rate over one cellular generation. Thus, κ combinatorially replaces the rate of degradation δ of the transcribed RNA with cell division time *t*.

CAP is the cost-aggregate of the promoterome.

*COST*_*t*_ and *COST*_*g*_ refer to the cost at the transcript or gene level, respectively; in various representations, they are suffixed by the technique used for TSS identification: CAGE or PRO-cap. The aggregated costs of the promoterome for any *N* or *C* samples are abbreviated as *CAP*_*N*_ and *CAP*_*C*_, respectively.

Notably, the cost of transcription is in terms of the number of phosphate bonds hydrolyzed. Since the nucleotides incorporated in a transcript can get recycled and much of the ribonucleotide unit remains chemically intact during the process, the cost cannot be expressed in terms of nucleotide count but only in terms of the estimates of the actual phosphate bonds hydrolyzed.

The calculated cost aggregated across TSSs of a gene for 52,185 TSSs from CAGE or PRO-cap are compiled sample-wise and presented in Supplementary Table S24 for CAGE and Supplementary Table S25 for PRO-cap.

### 2.11 Statistics and data visualization

All statistical analyses were performed on R version 4.4.1 (14/06/2024). All randomizations and operations involving randomizations were carried out using the R *base* package of the aforesaid version. All paired tests were performed using the *wilcox.test* function of the *base* R package with the argument *paired* set to *T* if the test was a paired Wilcoxon signed-rank test or *F* if it were a Wilcoxon rank-sum test. Statistical details of the data presented in the manuscript are summarized in Supplementary Table S27. The statistics table includes false discovery rates calculated as the fraction of bootstrap iterations that gave the same result for a variable when tested over iterations. For paired data, we swapped the *N* and *C* identities for 50% of the data as a measure of false positives, while for unpaired data, we pooled both groups and randomly sampled a pseudo identity. Plots were generated using the *ggplot2* package of *tidyverse*. The plots corresponding to Gene Ontology and the substitution simulation were made with GraphPad Prism 10.4.1. The interpolation was done using Origin 2017. All schematics were custom made on Apple Keynote. All custom codes are made available on the GitHub repository (https://github.com/umash-singh/d-FuTr).

## 3 Results

### 3.1 Curation of CAGE and GENCODE data reveals an energy-conserving design of TSS deployment and its deregulation in cancers

The human FANTOM CAGE data (Noguchi et al., 2017) provide TSS coordinates, their CAGE scores, and their gene affiliations. We combined them with the GENCODE annotations, which provide start codon coordinates for each gene. The most upstream start codon for any gene was designated as the only one to which all TSSs of the same gene would be mapped. This implied that some TSSs, which were present downstream of the designated start codon, were excluded from our analyses. The distance between TSSs (start of the CAGE peak coordinates) and the start codon was abbreviated as d (Supplementary Table S1; Figures 1A,B; Supplementary Material, schematic 3). d is essentially a count of nucleotides in GRCh38 obtained as a difference in the positions of TSSs and the associated start codons (Supplementary Table S2). All references to d (and its derivatives) henceforth imply the base count as units. This yielded a total of 57,461 TSSs from 16,302 protein-coding genes for our analysis with approximately three TSSs per gene on average, and at least 76% of the genes had more than one TSS. For a large population of TSSs (∼60%), the d values were 1 kb or less (Figure 1C). The <1 kb d tendency was prevalent for the TSSs even when the d profiles were calculated per gene as a harmonic mean of d ( HMd ; see Supplementary Material, Section 3.1; Supplementary Table S3) of the constituent TSSs (Figure 1C). An incremental d profile (a ratio of the d difference between two consecutive TSSs of a gene and the lower d in the pair) obtained by a gene-level analysis of TSSs recapitulated the pattern of d restriction (Supplementary Table S2; Supplementary Figure S1). Referring to a particular TSS located at d for each gene, the consecutive >1 kb TSSs for the majority of genes showed a d increment of about 50% only, with a large population exhibiting a d increment restriction between 4% and 0.1% (Supplementary Figure S1). It follows from these observations that the <1 kb TSS deployment presents an advantage that has been accorded to most if not all genes through the process of evolution.

**FIGURE 1:**
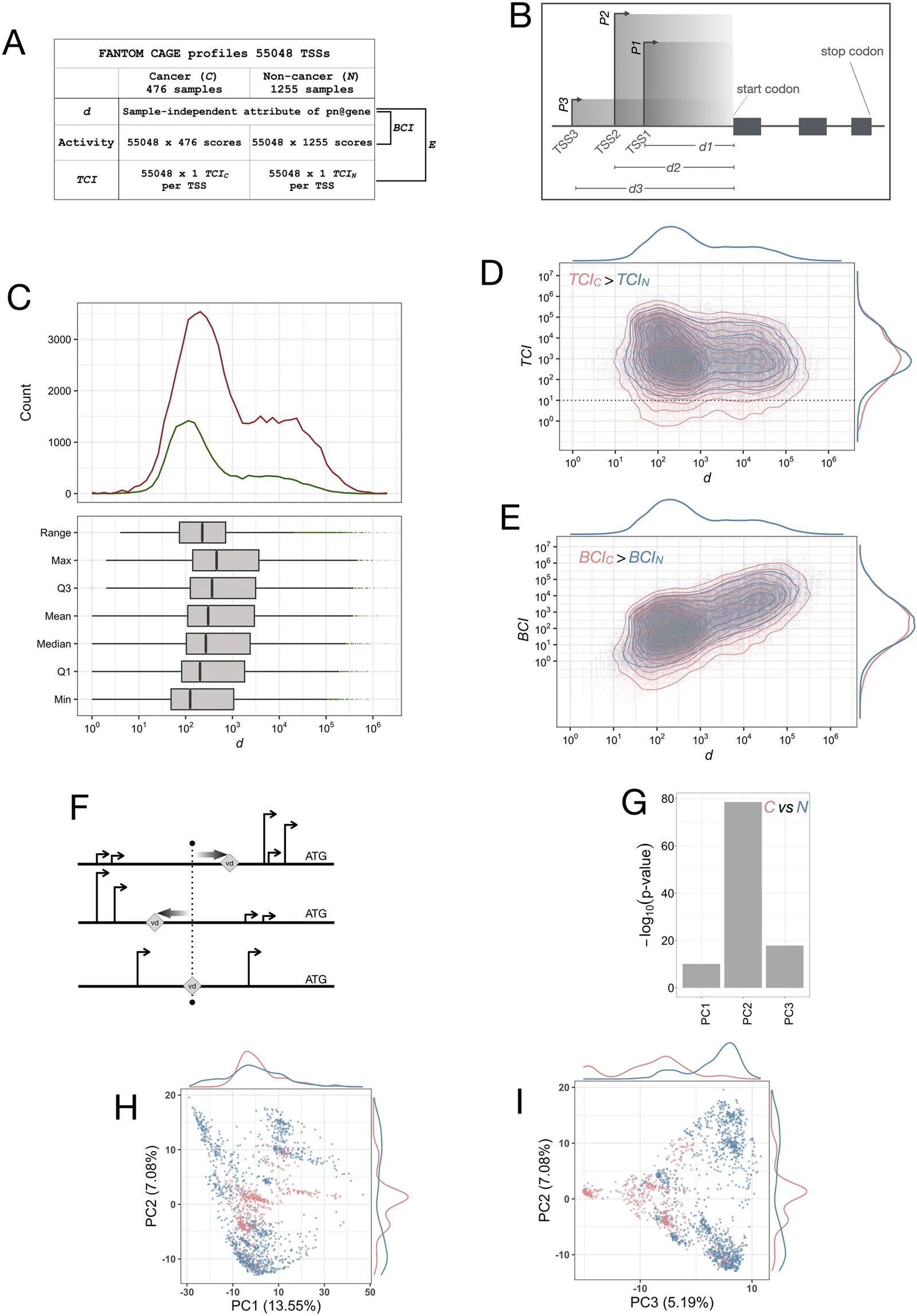
Minimization of the energy cost coefficient of transcription ( E ) by proximation of length transcribed upstream of the start codon ( d ) and transcriptional activity ( TCI )-weighted d ( vd ) distinguishes cancers from non-cancers **(A)** The schematic summarizes CAGE data curation based on GENCODE annotations of start codons for derivation of the parameters d , TCI , BCI , and E and CAGE metadata-based classification of samples into cancers (*C*) or non-cancers (*N*). **(B)** Diagrammatic representation of a hypothetical gene with an ORF marked by start and stop codons and three TSSs with corresponding d ( d1,d2,and d3 ) and activities *P* (*P*1, *P*2, and *P*3), respectively. The area shaded in gray shows the product d∗P and represents the energy cost coefficient E equivalent associated with each TSS. **(C)** The distribution of d values for 56,412 TSSs (red) shows a proximal concentration of TSSs, with the majority concentrated in the range 100–1,000 bps (x-axis). A gene-level d (calculated as a harmonic mean of d of all TSSs for a gene, HMd ) shows that this TSS-level d profile is also distributed across 16,093 genes. **(D)** TCI , the share of a TSS in the aggregated promoterome from a set of samples classified as cancer (*C*) or non-cancer (*N*), is significantly higher in cancer (Wilcoxon signed-rank test paired by 55,048 TSSs, *p*-value= 7.846e-3). Despite the difference, throughout the spectrum of d (x-axis), leaky low-level transcription is visible in *C* (demarcated by the dotted line at TCI value 10 on the y-axis). **(E)** A population size-independent parameter base count index ( BCI ), calculated as a share of CAGE score*d for any TSS in the aggregated promoterome, recapitulates the excess futile transcription in *C* over all ranges of d ( $BCI_{C}>BCI_{N}$ , Wilcoxon signed-rank test paired by 55,048 TSSs, *p*-value < 2.2e-16). **(F)** Schematic representation of effective d ( vd ) using three hypothetical genes with different profiles of TSS locations (vertical arrow heads at different distances upstream of ATG) and activities (heights of the arrowheads). Since vd (gray diamonds) is calculated as an activity-weighted d from all TSSs for every gene, a d -dependent difference in activity would affect the magnitude of vd . The lowermost gene exemplifies a simple case where vd is equidistant from two equally active TSSs (dotted marker), whereas the other two genes have their vd located more proximally (topmost) or distally (middle) due to a combination of d , activity, and number of TSSs. **(G–I)** Principal component analyses of the vd value show clear separation between *C* and *N* despite a large amount of intrasample group heterogeneity in *N*. Levene’s test of PCA values for the first three components shows a highly significant difference between *C* and *N* **(G)**. Marginal densities along the top and right of the PCA scatterplots highlight the differences between *C* (pink) and *N* (blue).

The d values for all the TSSs were benchmarked against the known 5′ UTR lengths (Supplementary Figure S2). As expected, the d values were higher than the UTR lengths due to the non-exclusion of introns in d calculation even though for the majority of TSSs, the d values showed a distribution similar to that of the 5′ UTR lengths (Supplementary Figure S2). Unlike 5′ UTR lengths, however, a substantial number of TSSs showed much higher d values, ranging over two orders of magnitude (Supplementary Figure S2). Such a disconnect between d and 5′ UTR lengths showed that despite concentration of TSSs < 1 kb, the transcriptional regulatory constraints built into the genome enforce transcription from some start sites located so far from the start codons that they vastly exceed the 5′ UTR length. Thus, the transcription regulation through TSS deployment entails futile transcription not necessarily required for downstream translation regulation.

We hypothesized that if alternative TSS deployment does not affect the downstream translation process, then the normal cells would tend to minimize futile transcription; in a “one gene-multiple TSS” system, high- d TSSs must offer distinct regulatory advantages to get deployed. Altered energy metabolism distinguishes transformed cells from normal ones, and as such, the normal energy-conserving nature of TSS deployment would decompose in cancers, leading to an overall more futile transcription through a dysregulated TSS deployment. To test this hypothesis, we compared CAGE data from non-cancer and cancer samples in multiple different ways.

CAGE samples were classified as non-cancer (*N*) or cancer (*C*) through a curated process (Figure 1A; Supplementary Table S4; Supplementary Material, schematic 2) based on the CAGE metadata, and the associated scores for all TSSs were collated (see Methods, 2.2). The representations of TSSs were expectedly non-uniform within, as well as unequal between, the *N* and *C* sample groups. A major fraction of TSSs were consistently detected similarly across >95% of *N* and *C* sample groups (Supplementary Figures S3A,B). However, a significant subset of TSSs had a low intragroup representation of <5%. The number of TSSs represented in <5% samples was remarkably higher in *C* (11,144) than that in *N* (8,095). Of these TSSs with low representation, 5,741 TSSs were common to both *N* and *C*, leaving only 2,354 and 5,403 TSSs unique to *N* and *C*, respectively. Overall, a total of 55,048 TSSs were commonly listed in the *N* and *C* sample groups, whereas 1,346 and 18 TSSs were exclusive to the *N* and *C* populations, respectively. Of the entire spectrum of TSS representation frequency in *N* or *C* only, the TSSs with <5% representation showed a significantly higher d in *C* than that in *N* (Supplementary Figure S3C; Wilcoxon rank-sum test, *p*-value = 8.755e-4). Removing the *N*-and *C*-exclusive TSSs from the comparison did not affect this finding (not shown). The TSSs deployed differently in *C* and *N* (TSS detected in <5% of either *C* or *N*) have a sample-type preference for deployment. Stochastic TSS deployment is unlikely to give rise to such a *C-* or *N*-biased TSS deployment profile. Thus, if the sample type-specific low representation frequency of TSSs is a representation of non-randomness with which TSSs get deployed, then these data indicated that TSSs with less stochastic deployment in *C* have a higher d .

To analyze the overall TSS deployment rates in *N* or *C* (Supplementary Table S5) without any effect of the differences in sample counts, we worked out a population size-independent parameter to represent the relative TSS deployment frequency. The share of each TSS in the total CAGE score count was separately derived for *C* or *N* sample pools and used as a transcript count index ( TCI ), a TSS-specific parameter representing its net deployment frequency in a sample group regardless of the intrinsic intersample variations scaled by 1e9 (Supplementary Table S6, Table 1 Equation *VI*). The choice of this multiplier does not affect intersample comparisons and can be used universally to represent TSS deployment count-derived parameters as “units per billion bases transcribed.” Overall, ${TCI}_{C}$ was significantly higher than ${TCI}_{N}$ (Supplementary Figure S4) even when 54.32% TSSs had ${TCI}_{C}<TCI_{N}$ (Wilcoxon signed-rank test paired by 55,048 TSSs, *p*-value = 7.846e-3). The ${TCI}_{N}$ and ${TCI}_{C}$ showed different relationships (Pearson and Filon’s z, *p*-value < 2.2e-16) with d (Spearman’s correlation coefficient r for ${TCI}_{N}$ versus d = −0.1468 and *p*-value < 2.2e-16, r for ${TCI}_{C}$ versus d = −0.1369 and *p*-value < 2.2e-16) (Figure 1D). While the low TCI was abundant in *C* at all d ranges (Figure 1D, dotted line threshold TCI of 10), the higher TCI in *C* was restricted primarily to a subset of proximal d TSSs. A comparison of ${TCI}_{\max}$ or ${TCI}_{\min}$ between *N* and *C* over all the possible d values showed two distinct patterns for <1 and >1 kb TSSs (Supplementary Figures S5A,B). The <1 kb TSSs in both *C* and *N* showed a wide range of relatively discrete ${TCI}_{\max}$ and ${TCI}_{\min}$ at the extremes with missing intermediate range TCI values. At >1 kb TSSs, however, the ${TCI}_{\max}$ and ${TCI}_{\min}$ showed a continuous distribution. This suggested that a range of transcriptional output is achieved, presumably through a wide range of regulated deployment limited to >1 kb d TSSs (Supplementary Figures S5A,B). Interestingly, at >1 kb TSSs, leaky transcription was detected in *C* as low levels of ${TCI}_{\max}$ were pervasive over all d ranges. Supporting this, across all the d values, only ${TCI}_{\min}$ was lower in *C* than in *N* (Wilcoxon signed-rank test paired by 55,048 TSSs, *p*-value <2.2e-16), while ${TCI}_{\max}$ did not exhibit any difference. Thus, clearly, there are *C*- and *N*-specific d -dependent patterns of TSS deployment. The concentration of TSSs proximal to start codons concomitant with a difference in d -dependent TSS deployment indicated that the futile transcription could be different between *C* and *N*.

### 3.2 Energy-conserving nature of TSS deployment depends majorly on the minimization of d

CAGE scores derived from tag counts represent TSS activity measured as capped mature transcripts originating from a particular site. The resource requirement for transcription from a particular site is directly proportional to the frequency of TSS deployment, measured relatively as the steady state concentration of capped RNA. Ignoring the cellular needs and biological advantages of a specific TSS selection for a particular protein synthesis, it follows mathematically that a proximal TSS selection would minimize the transcriptional resource cost for a gene. Assuming that the TSS location and deployment are orthogonal to each other, the resource cost can be represented by a dot product of TSS deployment (CAGE scores) and upstream excess distance from the start codon ( d ). In principle, this operation can be performed by using the TSS deployment data from not just CAGE but any technique that reports TSS firing as tag counts. The actual energy costs of transcription from that TSS would depend on a combination of constitutive energy costs of transcription (TSS-invariant parameters, such as the energy cost of ribonucleotide biosynthesis and RNA polymerase activity, innate to the mechanism of transcription) and $E_{t}$ (a TSS-variant parameter including the frequency of transcriptional firing represented by TCI and d ; Figure 1B). The net energy cost coefficient of transcription from a TSS could be expressed as a product of TCI and d . $E_{t}$ for *C* and *N* (Table 1 Equation *VII*; Supplementary Table S6) was compared for 55,048 TSSs. $E_{tC}$ was higher than $E_{tN}$ for 45.68% TSSs and lower than $E_{tN}$ for 54.32% TSSs, with overall $E_{tC}$ having a significantly lower value than $E_{tN}$ (Wilcoxon signed-rank test paired by 55,048 TSSs, *p*-value < 2.2e-16; Supplementary Figures S6A,B). This was unexpected as ${TCI}_{C}$ was significantly higher than ${TCI}_{N}$ (Wilcoxon signed-rank test paired by 55,048 TSSs, *p*-value < 0.05; Supplementary Figure S4). Interestingly, however, gene-level E ( $E_{g}$ ) derived by summation of $E_{t}$ of all TSSs separately for *N* $\left(E_{gN}\right)$ and *C* $\left(E_{gC}\right)$ for a gene (Table 1 Equation *VIII*; Supplementary Table S7) was significantly higher for *C* than that for *N* (Wilcoxon signed-rank test paired by 15,861 genes, *p*-value < 2.2e-16; Supplementary Figures S6C,D). $E_{gC}$ was higher than $E_{gN}$ for 52.23% genes, suggesting that the $E_{t}$ differences between *N* and *C* are not random TSS-specific attributes. Rather, the TSS-level E contributes to gene-level E , thereby connecting transcription energy cost coefficient with gene function.

The results so far showed that widespread alterations in TSS deployment are a feature of transcriptional dysregulation in cancers. A net effect of this altered TSS deployment is an increase in transcriptional resource cost. This would only be possible if the altered TSS deployment in cancers was a net use of larger d . Assuming that the *N* and *C* samples from which we analyzed the CAGE data represent the generic TSS deployment properties of non-cancer and cancer cells, respectively, we sought to quantitatively test the possibility of increased d deployment in cancers. This was done by a TSS-level paired analysis of consecutive distal-to-proximal deployment ratios using TCI . This analysis demonstrated an increased distal TSS utilization in *C* for every relatively proximal TSS of a gene (Supplementary Figures S7A,B). We generated a base count index ( BCI ) for the *N* and *C* sample pools (Table 1 Equation *IX*; Supplementary Table S8) that represents the share of deployment of each TSS in the transcription of d out of the total d deployment transcription of a sample group. In $E_{t}$ , TCI represented the TSS activity as relative to the sample group, but d remained absolute. In BCI , however, both TSS activity and d were combined relative to the sample group. In TSS-paired comparisons, $BCI_{C}>BCI_{N}$ (Supplementary Figure S8; Wilcoxon signed-rank test paired by 55,048 TSSs, *p*-value < 2.2e-16) with an overall high variance in cancers (Figure 1E) unlike $E_{t}$ , which was $E_{tC}$ < $E_{tN}$ . Both $E_{t}$ and BCI have two components, TSS activity and d .

Such a decomposed comparison of transcriptional resource cost for TSSs independent of their gene affiliations reinforced the importance of futile d transcriptional cost, TSS localization with respect to the start codons, and their deployment. Through a direct proportionality between BCI , on one hand, and TCI and d , on the other, the following deductions could be worked out:

BCI∝TCI*d,

$$\kappa =\frac{BCI}{TCI*d}.$$

The constant κ is a factor that represents systemic d -functionalization (including the rate of d -deployment and RNA turnover over the duration of one cell cycle). Since the population aggregates of the terms BCI , TCI , and d are identical for both *N* and *C* (see Supplementary Material, Sections 5 and 7), the factor κ is bound to be different between *N* and *C*. We could calculate κ separately for *N* and *C* as follows: $\kappa_{C}$ = 2.71991e-4 and $\kappa_{N}$ = 2.48027e-4. Thus, $\kappa_{C}$ was 9.6615% higher than $\kappa_{N}$ . It followed from these findings that each TSS in cancers is likely to conduct a higher d -dependent futile transcription by this relative magnitude.

However, d is a real TSS-level parameter encoded in the genome. For each gene, a fixed mean d could be represented by a harmonic mean of the d values of its TSSs ( HMd ) (Figure 1C; see Supplementary Material; Section 3.1). TSS deployment (represented by CAGE scores) functionalizes HMd for each gene in a sample-specific manner, giving rise to a virtual effective d for each gene. The effective d for the genes were calculated as a TCI -weighted mean d ( vd ) of all contributing TSSs (Table 1 Equation *X*; Supplementary Table S9). HMd represents an expected effective d for a gene if all the TSSs were equally deployed. In contrast, vd represents the actual effective d , which factors in the inter-TSS deployment differences with high vd indicating an overall higher preference for distal TSSs for a gene (refer to schematic, Figure 1F). vd , which was higher than HMd in *N* and *C* (not shown), was also overall higher in cancers (Wilcoxon signed-rank test paired by 15,861 genes, *p*-value = 2.21e-35; Supplementary Figure S9A). vd showed the power to classify individual *C* samples from *N* samples (Figures 1G–I). Thus, vd functioned as a parameter representing the differences between $E_{gC}$ and $E_{gN}$ in the context of d at the gene level. If these properties of vd are functionally relevant in cancer, we expected functional category enrichment in genes ranked by vd . Genes were ranked by ${vd}_{C}/{vd}_{N}$ ratios, and terminal quartiles (Supplementary Figure S9B) were compared for functional category enrichment (Supplementary Table S14). The low vd quartile, with a decreased vd in cancers, was rich in GO BP terms pertaining to biosynthesis, cell proliferation, cell cycle, and GO MF terms associated with DNA metabolism (Supplementary Figures S10A,B). The upper quartile genes were rich in GO BP terms pertaining to cellular signaling and GO MF terms linked to signal transduction mechanisms (Supplementary Figures S10A,B).

These results suggest that TSSs are deployed through different d -dependent mechanisms in *N* and *C*, resulting in different TSS deployment frequencies. This gives rise to a net higher energy cost coefficient of transcription in *C* at the gene level. This deregulation apparently manifests as a pan-cancer feature even if the *N* and *C* sample groups have internal heterogeneity. Stochasticity being a key mechanism of TSS deployment, we next analyzed the stochasticity of TSS deployment in *N* and *C* deducible from the CAGE data and tested if stochastic TSS deployment relates with d differently in *N* and *C.*

### 3.3 Gene-space entropies show that mitigation of futile d transcription through stochastic TSS deployment is bypassed in cancers

The genome offers a proximal concentration of TSSs in the gene space, and a stochastic deployment of all TSSs would tend to deploy <1 kb TSSs more than the >1 kb ones for every gene. For any given gene, an alteration in stochastic TSS deployment would affect proximal versus distal TSS preferences and a subsequent alteration in E . We estimated the stochasticity associated with TSS deployment of a gene using Shannon entropy in the gene space ( gH ) for the *N* and *C* populations (Supplementary Table S10; Table 1 Equation *XIII*). For any two genes with the same number of TSSs, the gene that deploys all its TSSs more uniformly (majorly stochastic deployment) would therefore have a higher gene-space entropy than the gene that deploys TSSs preferentially (majorly deterministic deployment) (Figure 2A). We found that the gene-space entropies are similarly distributed in *N* and *C*, but the overall $gH_{C}$ was lower than $gH_{N}$ (Wilcoxon signed-rank test paired by 11,928 genes, *p*-value = 1.364e-09; Figure 2B), showing that stochastic deployment of TSSs in the gene space is the dominant mechanism that is resisted in cancers.

**FIGURE 2:**
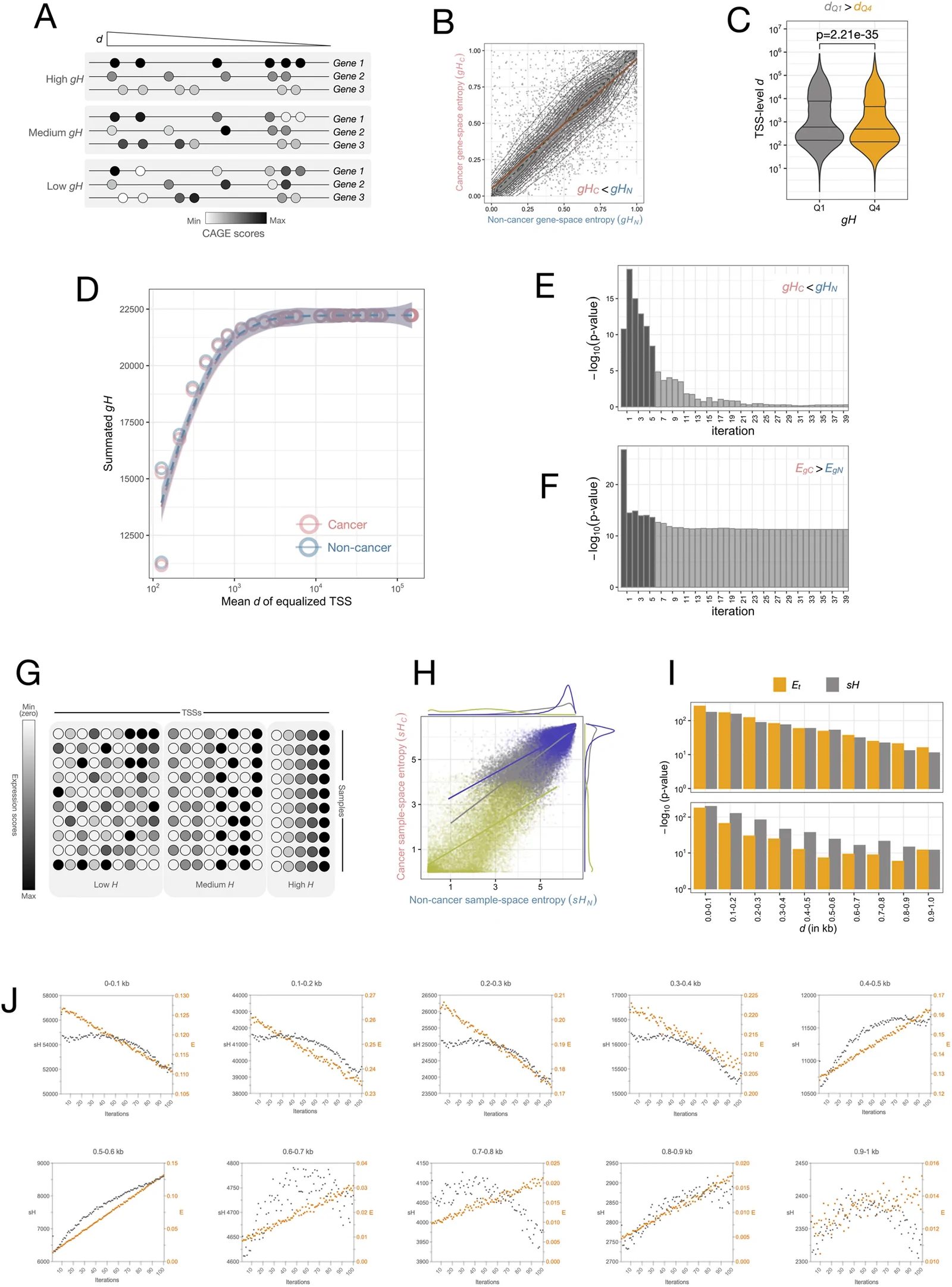
With higher sample-space entropy ( sH ), an E -conserving preference for proximal TSSs through gene-level stochasticity of TSS deployment ( gH ) is systemically lost in cancers but retained for genes supporting biosynthesis and cell proliferation. **(A)** Schematic showing stochasticity of TSS deployment using a hypothetical set of three genes under conditions of high, medium, or low gene-space entropy. The gradient of d indicated at the top shows the location of TSSs (circles in shades of gray representing TSS deployment frequencies calculated from CAGE expression scores), with the ends marked “Gene” representing the minimal d at the start codon. Similar deployment frequencies of all TSSs of a gene generate high gene-space entropy (topmost). Regulated and restricted deployment of some TSSs generates low gene-space entropy (bottom). **(B)** Overall gH is highly similar between *C* and *N* but significantly lower in *C* (Wilcoxon signed-rank test paired by 11,928 genes, *p*-value = 1.364e-09). **(C)** Genes with low gH in cancers have high d TSSs and *vice versa* (Wilcoxon rank-sum test, *p*-value < 2.2e-16), indicating an association between TSS deployment and d . **(D)** A forced increase in gH by equalization of TSS scores starting from the most proximal two TSSs and proceeding toward consecutive distal TSSs shows that *C* retains overall higher gH than that of *N* (y-axis) due to the stochastic deployment of TSSs, with d up to 1 kb (x-axis). **(E,F)** The loss of gH **(E)** with a concomitant increase in $E_{g}$ **(F)** in *C* compared with *N* is highly significant (y-axes) for the first five TSS score-merging iterations (x-axes). **(G)** Schematic representation of stochasticity of TSS deployment, deduced from the entropy of CAGE scores of each TSS in the sample space ( sH ). The intersample variation of deployment probabilities for a TSS (along the horizontal axis) can be high (low sH , left panel), moderate (medium sH , middle panel), or very low (high sH ). **(H)** sH is correlated between *C* and *N* for all TSSs (gray dataset). When split into TSS with non-zero representation in minimum 100 samples (*frequently deployed* TSSs, blue) or *non-frequently deployed* TSSs (olive green dataset), distinct patterns of ${sH}_{C}>{sH}_{N}$ for *frequently deployed* and ${sH}_{C}<{sH}_{N}$ for *non-frequently deployed* TSSs emerge. **(I)** sH -linked $E_{t}$ differences, calculated as log_2_ ratios of products of sH and $E_{t}$ , between *C* and *N* display an association with d . **(J)** Substitution of *C* CAGE scores with those from *N* decreases $E_{t}$ and sH in a d -dependent manner for TSSs with d <0.4 kb ( sH on the lefty-axis (black) and $E_{t}$ on the right y-axis (orange), 50 iterations of CAGE score substitutions from *C* to *N* on the x-axis). These data show that the unexpected correlation between the energy cost coefficient of transcription and consistency of a TSS deployment in cancers or non-cancers is tightly linked to the proximity of TSS to the start codon.

Next, the two terminal quartiles of genes sorted by the $\left({gH}_{C}/{gH}_{N}\right)$ ratios (Supplementary Table S10) were compared. The subset of genes with higher entropic TSS deployments in cancers had a significantly lower d and HMd profile of TSSs (Figure 2C Wilcoxon rank-sum test, *p*-value = 6.32e-18; Supplementary Figure S11 Wilcoxon rank-sum test, *p*-value = 9.19e-05). Supporting these results, we observed that although gH does not show a strong correlation with $E_{g}$ or d (not shown), it exhibited a bimodal distribution such that two groups of genes could be segregated by using a gH threshold of 0.4 (Supplementary Figure S12A). The subset of genes with gH>0.4 also had higher TSS d profiles (Wilcoxon rank-sum test, *p*-value = 6.32e-18, Supplementary Figure S12B), indicating a more regulated TSS deployment. The genes qualifying into the gH>0.4 category independently in *C* and *N* showed no significant differences in their TSS d profiles (not shown). Different sets of genes fell into the gH <0.4 category independently in *C* and *N*. The genes belonging to this low gH cluster in cancers had significantly higher d TSSs compared with those in non-cancers (Wilcoxon rank-sum test, *p*-value = 0.016; Supplementary Figure S12C). Although these results did not reveal if within a gene an entropic versus deterministic TSS deployment is linked to proximal or distal TSS shift, they did establish that stochastic TSS deployment is sensitive to the overall TSS d profile of a gene. Most importantly, the low gH preference in cancers for genes with high- d TSSs raised interesting possibilities that we investigated further.

To understand how the dependence of gene-space H on d differs between *N* and *C*, we calculated the gene-space H and associated $E_{g}$ for all genes by iteratively equalizing the activities of consecutive TSSs in a sorted order of d (Figure 2D; see Methods, 2.4). Through this mechanism, we increased the gene-space H by generating the maximum possible H for the first $\left(n+1\right)$ number of TSSs in every n ^th^ iteration of TSS activity equalization. The effects of <1 or >1 kb TSSs of any gene on its gene-space H and $E_{g}$ could now be established by observing how TSS equalization affected these parameters. We performed this analysis on TSSs sorted by d in ascending (from the most proximal toward the most distal TSS) or descending (from the most distal toward the most proximal TSS) order. Over the iterations, $gH_{C}$ remained less than $gH_{N}$ and eventually equalized past 10 iterations (Figure 2E; Wilcoxon signed-rank test paired by 11,928 genes per iteration, *p*-value < 1e-3). Similarly, $E_{gC}$ remained higher than $E_{gN}$ even if the change stabilized after 10 iterations (Figure 2F; Wilcoxon signed-rank test paired by genes per iteration, *p*-value < 1e-3). The majority of gH and $E_{g}$ changes were restricted to the equalization of the first five TSSs promoterome-wide (highlighted in Figures 2E,F). As >90% of genes have five TSSs or fewer (Supplementary Table S2 and not shown), a similar finding was also obtained through the equalization of the last five TSSs (Supplementary Figures S13A,B). These findings suggested that in a TSS landscape spanning over a wide range of d , stochasticity favors proximal TSS deployment in accordance with the d distribution of TSSs. The gene-space H excess in *N* over *C* is due to the preferred utilization of the first five TSSs, and this confers a low $E_{g}$ on *N* as compared with that on *C*.

gH being tightly linked to proximal TSS deployment and E differences between *C* and *N* raised the possibility that it would affect genes in a function-dependent manner. Similar to vd, we observed that the Q1 of genes ranked by $\left({gH}_{C}/{gH}_{N}\right)$ ratios were enriched in GO BP terms pertaining to cell proliferation and biosynthesis and MF terms linked to DNA/RNA binding (Supplementary Figures S14A,B; Supplementary Table S15). The Q4 set was enriched in GO BP terms associated with signal transduction, similar to that observed for vd (Supplementary Figure S10A; Supplementary Table S14) and MF terms associated with extracellular structure regulation and ribosomal function. Gene-space entropy is focused on looking at inter-TSS variability within genes for a population. However, it does not account for any sample-space heterogeneity of a population. To understand the role of intersample heterogeneity, we compared the TSS deployment probabilities in the sample space between the *N* and *C* sample groups.

### 3.4 Sample-space entropy reveals that selective deployment of high- d TSSs alongside a predominantly stochastic deployment of low- d TSSs is widespread in cancers

We hypothesized that (1) non-cancers would manifest tissue-type heterogeneity through selective TSS deployment, which would lead to high intersample variability in TSS activities, and that (2) a relative loss of tissue-specific gene expression patterns in cancers would reduce this intersample variability in TSS activities between samples with an effect on $E_{t}$ . This could be tested by a cancer versus non-cancer comparison of entropies for individual TSSs across the sample space. These sample-space entropies ( sH ) would be derived from the probabilities of deployment of each TSS across distinct samples (Table 1 Equation *XV*; Supplementary Table S11). An entirely stochastic deployment of TSSs would escape regulation and become similarly deployed in various samples (Figure 2G). Based on our findings so far, we argued that stochastic deployment of TSSs across samples would perturb the E of the system. To begin with, we needed to understand the relationship between sample-space entropy and $E_{t}$ in general, independent of the *N* or *C* sample identities. Similar to the TSS equalization simulation on gH (see Methods, 2.4), we performed an alternative simulation focused on the sample space with equal contributions from *N* and *C*. We iteratively equalized the scores of TSSs across all samples to steadily maximize the sample-space entropy and tested the corresponding effect on E . The sample space is gene-blind; therefore, all TSSs of a sample were equalized, unlike gH , where all TSSs of a gene were equalized (see Methods, 2.5). This controlled entropy increment of artificially generated promoteromes showed a strong increase in sH and $E_{t}$ as compared with the naturally observed sH and $E_{t}$ in *C* and *N* (Supplementary Table S13). Interestingly, there was a positive association between sample-space entropy and $E_{t}$ (Supplementary Figure S15) for all TSSs except those with d < 1 kb. This restrained range of actual sample-space entropy and associated $E_{t}$ as observed in *C* and *N* (Supplementary Figure S15) indicated that TSS deployment is tightly regulated in the sample space, with only a limited contribution from stochasticity. This regulated TSS deployment minimizes $E_{t}$ and restrains entropy in the sample space.

We next analyzed entropies in the sample space derived from TSS-specific CAGE scores (Supplementary Table S11) in different ways. First, we calculated sH (a schematic in Figure 2G) from *N* and *C* samples for a restricted set of TSSs with non-zero CAGE scores from at least 100 *N* and 100 *C* samples. As CAGE is biased to report only TSS activity, not inactivity, and since entropy values and associated information cannot be deduced from inactive TSS, such constraints allowed sample-space entropy comparisons between *N* and *C* just from active TSSs without any detection bias. Of the 55,048 TSSs reported in CAGE data, 33,232 TSSs satisfied these constraints (Supplementary Table S11). For these *frequently deployed* 33,232 TSSs, sH was strongly increased in cancers (Wilcoxon signed-rank test paired by 33,232 TSSs, *p*-value < 2.2e-16; blue label in Figure 2H). Thus, the *frequently deployed* TSSs are deployed with low intersample variability in cancers, whereas the intersample variability for these TSSs is higher in non-cancers. Conversely, the *non-frequently deployed* 21,816 TSSs (olive green label in Figure 2H) gave rise to an *N*-biased distribution of sH (Wilcoxon signed-rank test paired by 21,816 TSSs, *p*-value < 2.2e-16). The overall sample-space variabilities in TSS deployments between *N* and *C* (gray label in Figure 2H) could be classified into two different pools: the *frequently deployed* ones, which have higher sH in *C*, and the *non-frequently deployed* ones with lower overall sH in *C*.

To understand the cooperation between sH and $E_{t}$ , we compared the products or ratios of $E_{t}$ and sH for each TSS between *C* and *N* (Supplementary Figure S16A) over the entire range of d . The *non-frequently deployed* TSSs, which have lower sH and $E_{t}$ in *C*, also had lower $sH*E_{t}$ in *C* (Supplementary Figures S16B,C; *C* < *N*, Wilcoxon signed-rank test paired by 21,816 TSSs, *p*-value < 2.2e-16). The *frequently deployed* TSSs, however, showed a significant excess of $sH*E_{t}$ in *C* owing to their higher sH and cooperating $E_{t}$ (Supplementary Figures S16D,E; Wilcoxon signed-rank test paired by 33,232 TSSs, *p*-value < 2.2e-16). The *non-frequently deployed* TSSs by definition have high variability across samples with deployment in less than 100 samples. Thus, independent of their TSS activity, their sample-space entropy would remain lower than that of the *frequently deployed* TSSs. Such low deployment frequencies across samples would lower their overall TCI and consequently $E_{t}$ . Expectedly, the *non-frequently deployed* TSSs had a lower $sH*E_{t}$ in *C* (Supplementary Figures S16A–C, Wilcoxon signed-rank test paired by 21816 TSSs, *p*-value < 2.2e-16). The *frequently deployed* TSSs, however, showed the opposite behavior with sH and $E_{t}$ exhibiting a *C* bias. As a result, $sH*E_{t}$ was also higher in cancers for these TSSs (Supplementary Figures S16A,D,E, Wilcoxon signed-rank test paired by 33,232 TSS, *p*-value < 2.2e-16). It was interesting to note that the opposing behavior of $sH*E_{t}$ between the two categories of TSSs was driven by a cooperation of sH and $E_{t}$ . Such concomitant differences in sample-space variability and $E_{t}$ between *N* and *C* could be explained by the frequency of deployment and the distance. We thenceforth analyzed the influence of d -dependent TSS deployment on sH .

The $E_{t}/sH$ profiles were analyzed for various d bins (Supplementary Figure S17). We observed that for the *non-frequently deployed* TSSs, the nature of sH , $E_{t}$ , and $E_{t}/sH$ remained *C* < *N* at all d values, with the first 0–1 kb d bin being the most significant (*p*-values tend to 0, Supplementary Figure S17A). The *frequently deployed* TSSs, in contrast, showed an opposite difference (*C* > *N*) for sH , $E_{t}$ , and $E_{t}/sH$ (Supplementary Figure S17B; Wilcoxon signed-rank test paired by d bins, *p*-value < 0.05) except the last bin. However, the most significant difference between *C* and *N* for all three parameters ( sH , $E_{t}$ , and $E_{t}/sH$ ) were from the first d bin (0–1 kb) (Figure 2I, for $E_{t}$ and sH ). This suggested that the two categories of TSSs, *frequently deployed* and *non-frequently deployed*, have contrasting properties to balance the energy cost coefficient of transcription across the sample space in a d -dependent manner. To elucidate these differences further, we performed an incremental substitution of the *C* sample space with the *N* sample space and calculated sH and $E_{t}$ (see Methods, 2.6). These simulations using sH and associated $E_{t}$ differences between *N* and *C* for all TSSs reinforced that the lowest d bin of 0–1 kb accounts for the majority of differences (Supplementary Figure S18). A finer resolution analysis of these TSSs split into 0.1-kb bins showed that the sH and $E_{t}$ differences are concentrated in a d of about 0.4–0.5 kb specifically for the *frequently deployed* TSSs (Figure 2J). We could conclude that in *C*, high sH is associated with low d and high TCI (deployment) of TSSs leading to an overall $E_{t}$ excess in most TSSs. Further, by comparing sH -factored TCI and d , we found that sH drives high TCI and low d in cancers. With increasing sH , TCI increased more sharply in *C* as compared with *N* (Supplementary Figure S19A; Fisher’s z test for 55,048 TSSs, *p*-value = 0.0053). On the contrary, with increasing sH , d decreased more sharply in *C* as compared with *N* (Supplementary Figure S19B; Pearson and Filon’s z for 55,048 TSSs, *p*-value = 0). Reinforcing these findings, the same relationship between sH and TCI or d is obtained when using ratios ( d or TCI/sH ) instead of the product ( d or TCI*sH ) (not shown). These findings implied that cancers in general deploy low d TSSs with higher TCI and less heterogeneity across the sample space. By corollary, these results implied that high- d TSSs are deployed with low sH and low TCI . This mechanism apparently leads to an $E_{t}$ saving outcome in *C*, which is limited only to TSSs with d less than 0.5 kb. These results showed that (i) high- d TSSs undergo more selective deployment in *C*, (ii) stochastic deployment of TSSs conserves $E_{t}$ by retorting to low d in *N*, and (iii) even in *C,* the higher stochastic deployment targets a small subset of genes with a low d profile.

Similar to vd and gH , sH also showed an association with gene functions relevant to cancers. Genes ranked by ( ${sH}_{C}/{sH}_{N}$ ) ratios were subjected to functional category enrichment (Supplementary Table S16). The Q4 genes, representing an increased entropy in cancers, were enriched in genes involved in cell proliferation and biosynthesis (Supplementary Figure S20A) as GO BP terms and DNA/RNA binding as GO MF terms (Supplementary Figure S20B) and the Q1 genes primarily enriched in signaling (Supplementary Figures S20A,B). Combined with the gH findings, these results suggested that a low gH and high sH cooperate in cancers to achieve a regulated deployment of proximal TSSs for genes involved in core cancer hallmarks. We combined the gene and sample spaces to estimate the overall combined entropy affecting TSS deployment and its functional implications in cancer.

### 3.5 Compound entropy represents a system-level stochasticity by combining gene-level TSS selection with the sample-level heterogeneity

The effective stochasticity associated with the deployment of any TSS deducible from the CAGE scores would actually be a combination of the stochasticity in TSS deployment for any gene (estimated as gene-space entropy gH ) and stochasticity in TSS deployment across samples (estimated as sample-space entropy sH ). This combined stochasticity can be estimated by a compound entropy ( cH ) calculated from independent non-exclusive combinations of sample-and gene-space probabilities for each TSS (schematic in Figure 3A; Table 1 Equation *XVII*). From the compound probabilities, one cH per gene was calculated in the gene space (Supplementary Table S12).

**FIGURE 3:**
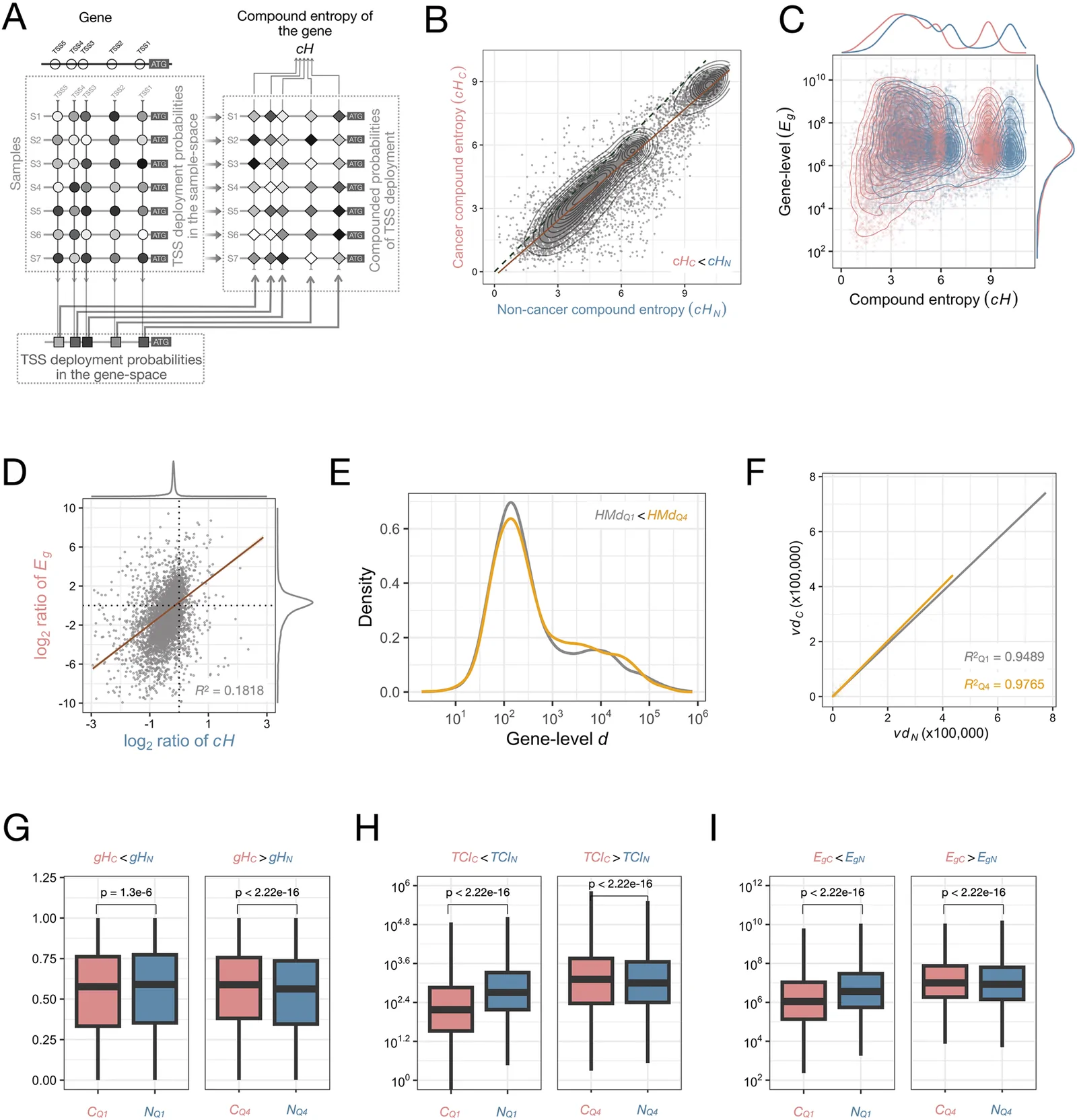
The overall stochasticity of TSS deployment ( cH ) favors a proximal transcription start and minimization of E at the gene level, which is disrupted in cancers. **(A)** Schematic representation of the calculation of compound entropy ( cH ) for a hypothetical system of a 5 TSS gene (TSSs labeled TSS1–TSS5) and seven samples (labeled S1–S7). ATG demarcates the start codon of the gene; the various TSSs are drawn at varying d as unfilled circles. Circles with gray shade depict sample-space probabilities, squares with gray shades depict gene-space probabilities, and diamonds with gray shades depict compound probabilities derived as products of the sample-and gene-space probabilities of each TSS. The compound-space probabilities are used to calculate one cH value per gene. Different shades of gray represent different probabilities derived from differing CAGE scores. **(B)** cH is tightly correlated between *C* and *N* but significantly lower in *C* (Wilcoxon signed-rank test paired by 11,928 genes, *p*-value < 2.2e-16). **(C)** cH shows a bidirectional relationship with $E_{g}$ . *C* has a larger range of $E_{g}$ than *N* at lower cH , with a conspicuous set of genes having low cH and low $E_{g}$ in *C* ( cH < 6; red arrow). At cH > 6, $E_{g}$ in *C* exceeds $E_{g}$ in *N*, with a set of genes having high cH and high $E_{g}$ in *C*. **(D)** Products of cH and $E_{g}$ show a strong correlation with a *C-*biased distribution showing that low cH and low $E_{g}$ co-occur more frequently in *C* than in *N*. **(E)** HMd of genes belonging to terminal quartiles Q1 (green) and Q4 (red) of cH ( ${cH}_{C}/{cH}_{N}$ ) shows that the HMd of Q1 is significantly lower than the HMd of Q4 (Wilcoxon rank-sum test, *p*-value = 1.729e-2), suggesting that genes with a high TSS d profile are more prone to stochastic TSS deployment in *C*. **(F)** vd scatterplots between *C* and *N* with a regression line for the same Q1 (*R* ^2^ = 0.9489; gray line) or Q4 (*R* ^2^ = 0.9765; orange line) genes show a significant difference between Spearman’s correlation coefficients (Fisher’s z test for 2,982 genes, *p*-value < 2.2e-16), with the Q4 gene set having a *C*-biased distribution. Results in **(B**–**F)** show that higher cH in *C* is associated with higher d , which increases $E_{g}$ . At the same time, the lower cH in *C* is associated with higher $E_{g}$ , but lower d , suggesting an activity-driven $E_{g}$ excess in *C* from low- d TSSs. **(G–I)** Consistent with the findings described above, we observed that gH **(G)**, TCI **(H)**, and $E_{g}$ **(I)** follow a *C < N* pattern for cH ( ${cH}_{C}/{cH}_{N}$ ) Q1 genes (Wilcoxon signed-rank test paired by 2,982 genes, *p*-values ≤ 1.3e-6), whereas these parameters follow a *C > N* pattern for the Q4 genes (Wilcoxon signed-rank test paired by 2,982 genes, *p*-values < 2.2e-16).

Before applying compound entropy calculations for differences between *C* and *N*, we sought to understand its relationship with $E_{g}$ in simulated promoteromes (see Methods, 2.5). Using the artificial promoteromes with a controlled increase in entropy, we established that cH and $E_{g}$ covary directly (Supplementary Figure S21). The cH – $E_{g}$ relationships from these simulations also suggested that the overall actual entropy of TSS deployments, although different between *C* and *N*, exists in a state of very low $E_{g}$ due to gene-wise bundling of TSSs. This low $E_{g}$ state is not achievable by disregarding a regulated gene-wise TSS deployment sensitive to gene function and TSS d profiles.

Comparison of cH between *C* and *N* showed an *N*-biased distribution with ${cH}_{C}<{cH}_{N}$ (Figure 3B; Wilcoxon signed-rank test paired by 11,928 genes, *p*-value < 2.2e-16). cH and $E_{g}$ comparisons showed different relationships between cH and $E_{g}$ in *C* and *N* (Figure 3C). At the gene level, $\left(C/N\right)$ ratios of cH and $E_{g}$ showed a highly significant positive correlation even if cH was tightly restricted to *C* < *N* (Figure 3D; linear regression coefficient *R* ^2^ = 0.1818, *p*-value < 2.2e-16). *C* showed a higher $E_{g}/cH$ than *N*, reinforcing that for every unit increment in cH , the increment in *C* is higher than that in *N* (Supplementary Figure S22; Wilcoxon signed-rank test paired by 11,928 genes, *p*-value < 2.2e-16). Next, we ranked genes by cH ratios from *C* and *N* and compared the terminal quartiles Q1 and Q4 (Supplementary Figure S23). The Q1 genes had a significantly lower HMd profile (Figure 3E; Wilcoxon rank-sum test, *p*-value = 0.017). Unlike HMd (the expected d representative of a gene if all TSSs were equally weighted, identical for *C* and *N*), the TCI -weighted d ( vd ) was higher (observed effective d for a gene) in cancers for the Q1 and Q4 genes (Figure 3F; Wilcoxon signed-rank test paired by 2,982 genes each, *p*-value for Q1 = 2.959e-12 and *p*-value for Q4 = 1.035e-13). Interestingly, three parameters, not directly factored in calculation of cH ( $E_{g}$ , TCI , and gH ) were lower in *C* than in *N* in the Q1 genes as compared with the Q4 genes (Figures 3G–I; Wilcoxon signed-rank test with *p*-values indicated above the bar plots). Ranked by the *C/N* ratios, the Q1 genes thus stood out as a set of genes for which low cH was tightly associated with low $E_{g}$ . The Q4 genes associated with a higher compound entropy in cancers also showed an enrichment of cell proliferation and biosynthesis genes like the parameters described earlier (Supplementary Figures S24A,B).

Calculations of all the entropies, including cH , were dependent on the CAGE scores but independent of the d profile of TSSs. To uncover how entropies in TSS deployment still affected TSSs in a d -dependent manner, and consequently gene-level E , we calculated a combined parameter of cH and vd . With the two parameters being inversely related to each other (Supplementary Figure S25), we calculated vd/cH (Figure 4A) and used its *C/N* ratios to rank genes (Supplementary Table S12).

**FIGURE 4:**
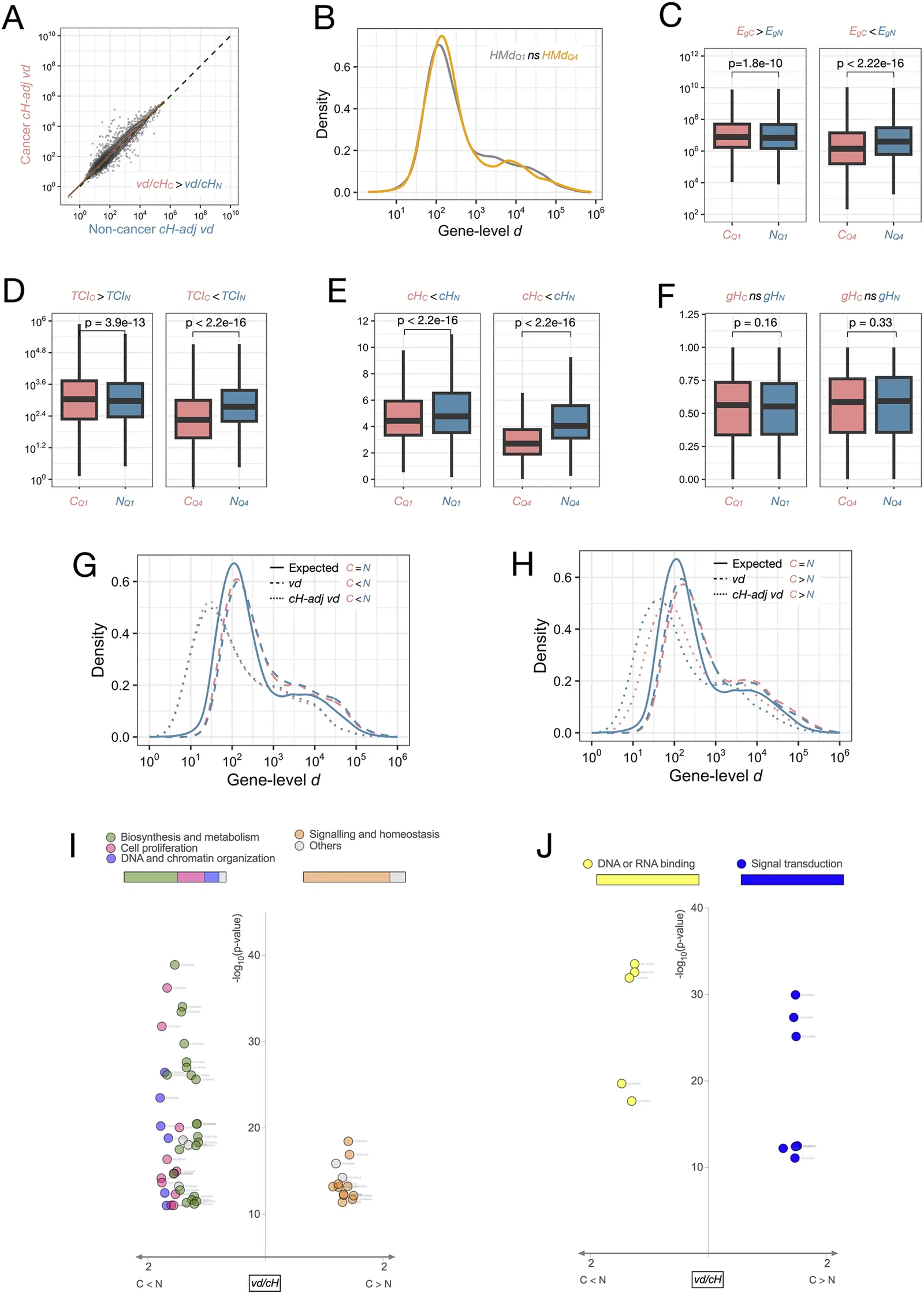
The minimizing effect of cH on distal TSS deployment, represented by vd/cH , is selectively impaired in cancers for genes involved in cell proliferation and biosynthesis. **(A)** A combination of vd and cH (calculated as vd/cH for each gene) is significantly lower in cancer (Wilcoxon signed-rank test paired by 11,928 genes, *p*-value < 2.2e-16). **(B)** The Q1 and Q4 genes sorted by log_2_ ratios of vd/cH from *C* and *N* show that, unlike for cH (Figure 5D), the compound entropy-factored effective d does not affect genes based on their TSS d profiles. **(C–E)** The Q1 and Q4 genes have highly significant different $E_{g}$ , TCI , and cH profiles between *C* and *N*; low cH -factored vd genes have higher $E_{g}$ **(C)**, TCI **(D)**, and cH **(E)** in cancers and *vice versa* (Wilcoxon signed-rank test paired by 2,982 genes in Q1 and Q4, all *p*-values ≤ 1.8e-10). **(F)** gH shows no significant differences between *C* and *N* for either Q1 or Q4 genes. **(G,H)** HMd (expected), vd , and vd/cH comparisons between *C* and *N* for Q1 **(G)** and Q4 **(H)** genes show that both vd and vd/cH are decreased in *C* for Q1 and increased in *C* for Q4 genes (Wilcoxon signed-rank test paired by 2,982 genes in Q1 and Q4, *p*-values < 2.2e-16). The data underlying **(G)** and **(H)** correspond to the information in Supplementary Table S3( HMd ), Supplementary Table S9 ( vd ), and Supplementary Table S12 ( vd/cH and their quartiles). **(I,J)** Functional category enrichment comparison between the Q1 and Q4 genes shows that genes belonging to the biological processes cell proliferation and biosynthesis **(I)** and molecular function DNA or RNA binding **(J)** are enriched in *C* as compared with *N* (details in Supplementary Table S14).

Ranked by the *C*/*N* ratios of vd/cH , there was no significant difference in the HMd profile of the Q1 and Q4 genes (Figure 4B). The $E_{g}$ of the Q1 genes were significantly greater than those of the Q4 genes in *N* and *C* (Figure 4C and not shown). The $E_{g}$ excess in *C* was limited to Q1(Figure 4C; Wilcoxon signed-rank test for the Q1 and Q4 genes each paired by 2,982 genes, *p*-values < 1.8e-10). This $E_{g}$ excess in *C* for the Q1 genes was driven by a combination of TCI and d , resembling the overall gene pool (Supplementary Figure S26A; $r_{c}>r_{N}$ and $r_{c}<r_{N}$ for Q1 and Q4, respectively, using Pearson and Filon’s z test, *p*-value < 2.2e-16). The deficit of $E_{g}$ in *C* for the Q4 genes was driven by a leaky deployment of TSSs across all d . For the Q4 genes, even if the effective d was increased in *C* ( ${vd}_{C}>{vd}_{N}$ ; Wilcoxon signed-rank test paired by 2,982 genes, *p*-value < 2.2e-16), the low TCI in *C* (Figure 4D) offset its effect, resulting in a net lower $E_{g}$ (Supplementary Figures S26B).

For the Q1 and Q4 genes, cH was lower in *C* than in *N* with no significant difference in gH (Figures 4E,F). The Q1 genes ranked by vd/cH between *C* and *N* were expected to have higher cH in *C* as compared with *N.* Intriguingly, however, the observed cH was strongly lower in *C* than in *N* for the Q1 genes. The only reason why vd/cH would be lower in *C* even when cH is extremely low is an over-compensatory decrease in vd for the Q1 genes. This extremely low vd in *C* was not inferable from gH as the Q1 and Q4 genes showed no significant differences in their gH between *C* and *N* (Figure 4F). Thus, the combination of vd and cH led us to gene sets with collaborating changes in vd and cH , two otherwise independent parameters. These compensatory changes in vd were evident through comparisons of vd or vd/cH between *C* and *N* for the Q1 (Figure 4G) and Q4 (Figure 4H) genes. These results showed that for the Q1 and Q4 genes, while vd is higher than HMd , vd/cH is lower (Wilcoxon signed-rank test paired by 11,928 genes for *N* and *C*, *p*-value < 2.2e-16; not shown). vd/cH was increased over a wide range of d for the Q4 genes in *C* as compared with *N* (Figure 4H; Wilcoxon signed-rank test paired by 2,982 genes, *p*-value < 2.2e-16). We next evaluated if cH and vd are functionally relevant and if their association with genes explains cancer hallmarks. vd/cH turned out to be the most efficient parameter for ranking the genes leading to functional categorization relevant to cancer hallmarks. Biosynthesis, metabolism, and cell proliferation were highly enriched in the Q1 genes ranked by the *C*/*N* ratios of vd/cH (Figure 4I; Supplementary Table S17). Most emphatically, these Q1 genes were almost singularly enriched in the GO MF terms corresponding to nucleotide metabolism (Figure 4J; Supplementary Table S17). The Q4 genes remained generic with an enrichment of biological processes and molecular functions relevant to signaling. Overall, through vd/cH , the functional categories (Figures 4I,J) that were enriched or depleted in *C* as observed through vd were largely retained (Supplementary Figures S10A,B). However, vd/cH was a much stronger classifier of *C* from *N* through the cancer hallmark relevant genes. vd/cH , a combination of vd and cH , is thus a useful parameter to reveal how TCI -weighted d is exerted in a regulated and non-stochastic manner.

These functional categories enriched in genes ranked by vd and vd/cH differences between *C* and *N* led us to something more fundamental. A pan-GeneOntology analysis showed that the changes in vd were not restricted to just the genes that we identified in our analyses. Rather, all gene mapping to those GO terms recapitulated the expected directions of the differences (Supplementary Figures S27A–R), suggesting that the effect we have observed is widespread throughout the gene functional category and tightly linked to gene function. These findings indicated that there is feedback from gene function on the d -linked TSS deployment for the entire gene set. We argued that if this is true, then we shall observe an effect on gene function feedback on TSS d evolution itself. A comparison of genome-wide TSS and gene-level d profiles showed that chromatin regulatory and cell proliferation-related genes have higher d profiles at the TSS and gene-level (Supplementary Figures S28A–D), whereas the genes related to biosynthesis and metabolism have a lower TSS d profile with no significant gene-level d differences (Supplementary Figures S28E,F). Genes mapping to GO terms discovered using vd/cH showed a similar *C* versus *N* differences for all parameters (Supplementary Figures S29A–R), including d (Supplementary Figure S30). In contrast, gene-level d was significantly lower for biosynthesis and metabolism genes.

Thus, genes have primarily evolved TSSs at varying d under feedback from their function. The secondary evolution of TSS deployment preferences in cancers further accentuate the d -dependent preferences of TSSs to adapt to their proliferative and biosynthetic needs. The d -dependent TSS deployment from generally lower d TSSs for biosynthetic genes would naturally support cancer cell proliferation. The low- d TSSs could have been selected for biosynthetic genes to support rapid early embryonic cell division under nutritional and metabolic constraints. In the context of cancerogenesis, these low-*d* TSSs become a driver for clonal selection of cells with low-cost transcription of biosynthetic genes. The most significantly different TSS and gene-level d profiles from the genomic profiles were those of the genes involved in chromatin regulation (Supplementary Figures S28 and S30), suggesting that the adaptive TSS d switches operate at these genes capable of rewiring the cancer epigenomes for a better proliferative and biosynthetic outcome for incipient cancer cells.

### 3.6 PRO-cap datasets reinforce the futile transcription excess and gene function-linked proximal TSS deployments in cancers

We next used publicly available PRO-cap datasets from NCBI GEO (Supplementary Table S18) to test if the findings obtained using CAGE datasets are also observed using an alternative and more real-time mRNA capture technique. PRO-cap datasets from completely independent sets of 305 *N* and 110 *C* samples were processed as described in the schematic diagram (Supplementary Material, Section 12). Samples were classified as *C* or *N* by referring to the metadata and application of the same classification schema as was used for CAGE datasets including elimination of any samples with embryonic stem cells or similar. PRO-cap data were not used for *de novo* peak calling. Also, since there is no standard PRO-cap peak to gene and start site annotation available, we retained the CAGE peak coordinates for the calculation of d . PRO-cap signals were obtained for the CAGE peaks, quantile normalized, and ascribed to respective TSSs. Thus, we were able to derive 52,904 TSSs for which non-zero signals were available using PRO-cap independently in *C* and *N* samples, or 52,185 TSSs with CAGE and PRO-cap jointly in *C* and *N* samples (see Section 2 for details). Neither the TSSs common to both techniques (52,185) nor the TSSs exclusive to either showed a significant deviation in the d profile from what was observed for CAGE-represented TSSs (data not shown). Restricting the analyses to either only CAGE-represented TSSs (55,048) or only PRO-cap-represented TSSs (52,904) did not affect the *C* versus *N* differences for all parameters ( TCI , $E_{g}$ , BCI , sample-filtered sH , cH , and vd/cH ) except for $E_{t}$ , vd , gH , and score-filtered sH (Supplementary Table S20). Having benchmarked the PRO-cap and CAGE datasets with each other for comparability, we restricted all further comparisons between *C* and *N* using CAGE or PRO-cap data only to a common set of 52,185 TSSs. Similar to the findings obtained using CAGE data, PRO-cap data also showed that TCI , $E_{g}$ , and BCI were increased in *C* as compared to *N* (Supplementary Table S20; Supplementary Figures S31A–C), while *vd* and cH were decreased in *C* (Supplementary Table S20; Supplementary Figure S31D,E). In a stronger reinforcement of low- d TSS deployment for cancer phenotype-supporting genes, we observed that PRO-cap data capture a highly entropic ( $gH_{C}>gH_{N}$ ) proximal shift of TSSs overall ( $vd_{C}<vd_{N}$ (Supplementary Table S20; Supplementary Figure S31D), resulting in overall $E_{tC}>E_{tN}$ (Supplementary Table S20). These data showed that by using CAGE data, the proximal shift was diluted by the background TSS activities with higher d and was discoverable only through a terminal quartile comparison of TSSs sorted by vd ascending. In contrast, by using PRO-cap data, this proximal shift was visible overall without having to depend on terminal quartile comparisons. Supporting this, we observed that PRO-cap captures the d -dependent differential deployments of TSSs in *C* more efficiently than CAGE at both extremes of d (high d and low d ; Supplementary Figure S32).

Independently, the PRO-cap signals captured the proximal shift consistently in *C* samples, resulting in a very low cH ( $cH_{C}<cH_{N}$ ) such that despite vd being significantly lower in *C* than in *N*, vd/cH was significantly higher in *C* than in *N* (Supplementary Table S21). Thus, PRO-cap captured the phenomenon of E excess in cancers through a consistently high deployment of proximal TSSs more robustly than CAGE. We next asked if the association of vd/cH with biosynthesis genes as observed using CAGE data is recapitulated using PRO-cap. PRO-cap-derived vd/cH values were used to sort genes (Supplementary Table S21), and terminal quartiles were compared using GOrilla. Emphatically, we observed a significant enrichment of biosynthesis and cancer phenotype-supporting genes (Supplementary Table S22). A total of 103 out of 165 GO BP terms obtained in Q1 versus Q4 comparisons using CAGE or PRO-cap were identical (each having a BH FDR-corrected *p*-value < 1e-5). Thus, we concluded that the discovery of gene function-linked shift in proximal or distal TSS deployments in cancers is not an artifact due to CAGE or a limited set of samples. Instead, it is a phenomenon robust enough to be determined in independent sets of cancer or non-cancer samples through two different techniques of mRNA 5′ end capture.

### 3.7 Excess cost of futile transcription in cancers is revealed similarly by CAGE and PRO-cap

The energy cost of transcription ( COST ) through d was calculated per sample per TSS independently using CAGE or PRO-cap data. The framework established elsewhere (Lynch and Marinov, 2015) was used for the calculation (details in Methods, 2.10 and Supplementary Material, Section 13). The costs were calculated dually; by ignoring exon-intron boundaries, as well as by including the introns and allowing a unitary splicing of one intron at one statistical instance thereby excluding any combinatorial splicing. Notably, between CAGE and PRO-cap data, the vd/cH patterns of *C* and *N* were highly similar (Figure 5A), whereas the TSS-level COST values ( ${COST}_{t}$ ) were less well correlated (Figure 5B; Supplementary Figure S33A). Gene-wise aggregated COST of TSSs were obtained to generate gene-level COST values ( ${COST}_{g}$ ; Supplementary Figures S33B,C) for *C* and *N* through PRO-cap and CAGE data. GO term enrichment analysis using terminal quartiles of COST *C*/*N* ratios (Supplementary Table S26) revealed that the biosynthesis and cell proliferation-related genes maintain low-cost transcription in cancers. Additionally, the COST of aggregated promoteromes ( CAP ) was obtained individually for each sample in CAGE (CAGE– ${CAP}_{N}$ and CAGE– ${CAP}_{C}$ ) and PRO-cap (PRO-cap- ${CAP}_{N}$ and PRO-cap- ${CAP}_{C}$ ) described in Supplementary Material (Section 14).

**FIGURE 5:**
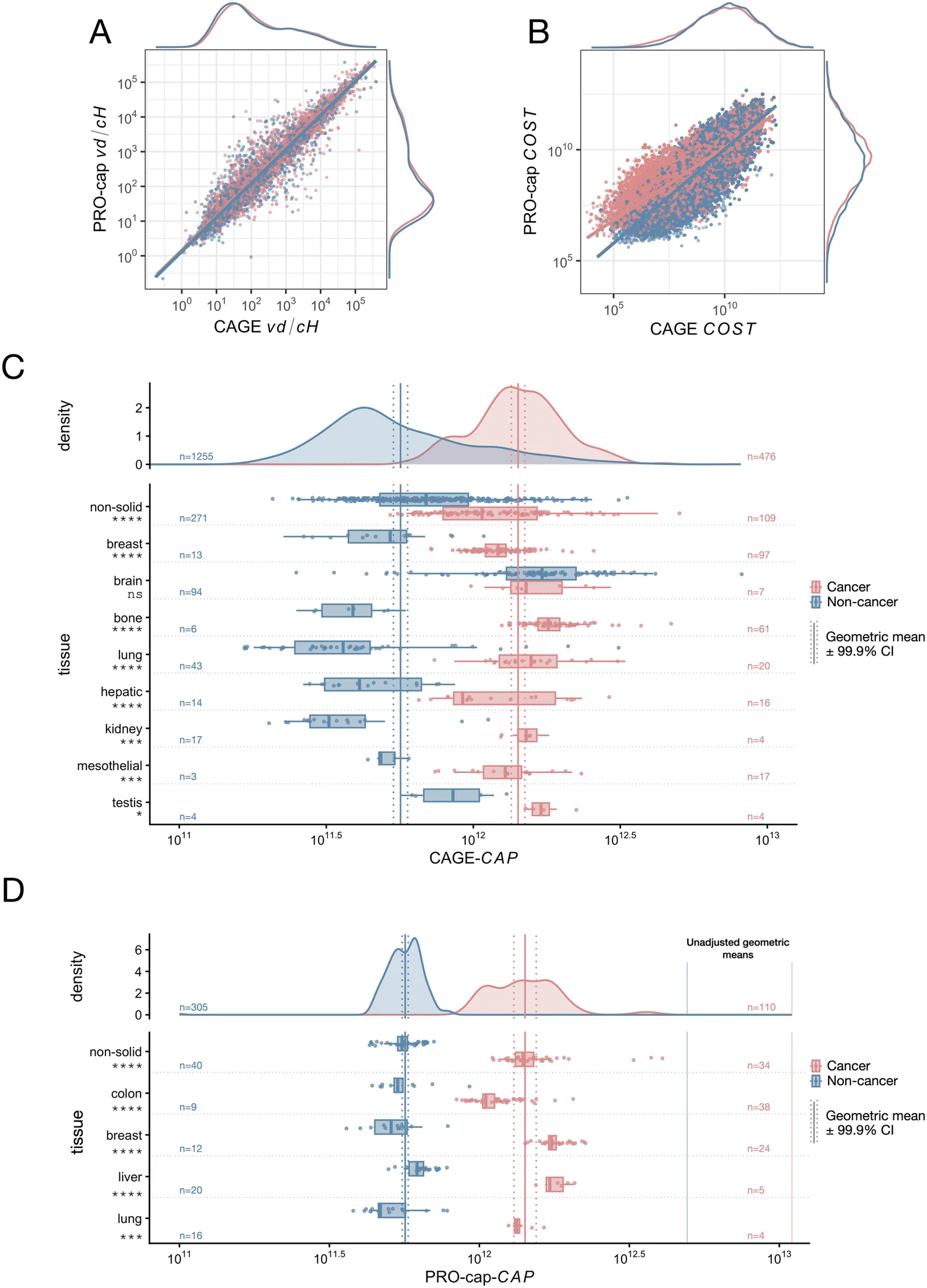
Energy cost of futile upstream transcription revealed by CAGE is reinforced by independently acquired PRO-cap data. **(A)** The most informative CAGE-derived parameter vd/cH showed a remarkable similarity with vd/cH obtained using PRO-cap data. **(B)** Cost of futile transcription for individual TSSs (including unitary splicing cost per intron) is correlated between PRO-cap and CAGE with obvious differences between *C* and *N*. The excess TSS-level costs observed in PRO-cap compared with CAGE are due to non-adjustment of the sequence data obtained from two independent techniques (PRO-cap data are ∼4 times denser than CAGE data) and differences in d -dependent RNA capture by the two techniques (Supplementary Figure S33). **(C,D)** Total promoterome-wide (52,185 TSSs) COST , calculated as “the number of high-energy phosphate bonds hydrolyzed per billion bases transcribed and unitary intron splicing in d, ” are shown for CAGE data **(C)** and PRO-cap data **(D)**. The top panels (labeled density along the y-axes) show promoterome-wide cost distributions for *N* (blue) and *C* (pink) for all CAGE samples **(C)** and all PRO-cap samples **(D)**. The lower panels in **(C,D)** show sample-level distributions of costs for selected tissue-matched pairs of non-cancers and cancers fetched from metadata (individual samples as dots along with box whiskers). The PRO-cap values plotted in **(D)** are downscaled (to account for differences in sequence data depths) to CAGE values using ratios of geometric means of PRO-cap and CAGE data for *C* and *N* independently. The vertical lines flanked by dotted lines mark geometric means and 99.9% CIs. In **(D)**, additional vertical markers show the unadjusted geometric means of costs in PRO-cap. Both unadjusted and adjusted PRO-cap and CAGE data demonstrate an excess COST in *C* over *N* by a factor of approximately 1e0.5. Sample counts are mentioned for each row independently. The asterisks represent *p*-value significance (*, *p*-value < 0.05; **, *p*-value < 0.01; ***, *p*-value < 0.001; ****, *p*-value < 0.0001; ns, non-significant).

These analyses showed that ${CAP}_{C}$ was significantly higher than ${CAP}_{N}$ (Figures 5C,D; for CAGE: geometric mean = 14.20e11 and with 99.9% confidence intervals (CIs) 13.47e11 and 14.98e11 for *C*; geometric mean = 5.66e11 and with 99.9% confidence intervals 5.35e11 and 5.98e11 for *N*; for PRO-cap: geometric mean = 110.21e11 and with 99.9% confidence intervals 101.17e11 and 120.06e11 for *C*; geometric mean = 49.31e11 and with 99.9% confidence intervals 48.18e11 and 50.46e11 for *N*).

The PRO-cap data were scaled to correct for systemic differences in mean sequencing depths between the two techniques and calibrated for CAP per billion bases transcribed. After this correction, strikingly similar cost excesses in *C* over *N* were observed using CAGE and PRO-cap (Figures 5C,D). The downsizing of PRO-cap data to CAGE data retained the same fold-excess of cost in *C* over *N* of ∼10^0.5^. The 99.9% CIs showed wide separation, suggesting that the cost parameter is a reliable predictor of *N* or *C* identity of samples (for scaled PRO-cap: geometric mean = 14.2e11 and with 99.9% confidence intervals 13.04e11 and 15.47e11 for *C*; geometric mean = 5.66e11 and with 99.9% confidence intervals 5.53e11 and 5.79e11 for *N*). Interestingly, we fetched metadata-derived tissue-matched pairs of *N* or *C* sample subsets for CAP comparisons. The results showed that while the cost excess in *C* is near-universally observed for all cancers (brain data for CAGE seem to be an exception), the extent of COST differences between *N* and *C* varies from one tissue type to another. Thus, the COST and CAP parameters provide cancer promoterome predictability with a significant level of confidence.

## 4 Discussion

Quantitative transcriptional deregulation in cancers has been widely reported. Qualitative changes in the transcriptome are however less well studied. Here, we have made the use of publicly available CAGE and PRO-cap data to understand how TSS deployment is qualitatively affected in cancers universally. The following hypotheses form the premise of our analyses: (i) that transcriptional deregulation in cancer is not only quantitative in terms of mRNA abundance but also qualitative in terms of commitment to transcribe from a particular start site; (ii) that transcription is an energy- and resource-intensive process and normal cells would maintain a more energy-conserving transcriptome; (iii) that deregulated transcription in cancers would be driven by energy addiction and associated wasteful transcription; (iv) that transcriptional resource optimization in normal cells would not only minimize unnecessary transcription initiation rather also preferentially avoid unnecessary stochastic transcription from distal start sites; and finally, (v) the flexibility of proximal versus distal TSS deployment would be sensitive to the gene functions with consequences on the adaptive needs of the cell.

A more stochastic TSS deployment allows for random combinations of gene expressions, of which those favoring cell proliferation and survival would be selected in an evolving tumor cell population. The advantages of such a strategy for competing tumor cell subpopulations are multiple. Mutations that could alter gene expression or gene dosage to the selection advantage of cancer cells take long to manifest, whereas stochastic TSS deployment for cancer phenotype-supporting gene expression allows a bypass to this dependence of cancer cells fitness on mutations. However, this function-relevant increased stochasticity of TSS deployment in cancer cells is likely to be affected by local epigenetic landscapes. Since some basal activity of TSSs, especially the distal ones, are DNA sequence encoded, any stochastic and ectopic TSS deployment will offer combinations of gene expressions without being limited by mutations and epimutations affecting them. Stochastic TSS deployment thus provides a molecular basis for multifaceted phenotypic fluidity to evolving tumor cell populations. We show that, for genes belonging to the functional groups biosynthesis and cell proliferation, a lower stochasticity of TSS deployment favors an energy-conserving proximal TSS in accordance with the evolved TSS d profiles in the human genome even if the net $E_{g}$ remains higher due to a disproportionately high TCI . The biosynthesis and cell proliferation gene enrichment in the less stochastically deployed TSS set is accompanied by an exceedingly stochastic deployment of TSSs from high- d TSSs; the latter has no strong functional category enrichment other than processes such as signal transduction, which do not directly and necessarily confer a proliferative advantage on cancer cells. Such a high- d TSS deployment is a gene function agnostic feature of stochastic TSS deployment that seems cancer type independent and highly energy intensive. Alongside genes involved in biosynthesis and cell proliferation, an increase in the regulated deployment of chromatin regulatory genes with molecular functions involving DNA or RNA binding is also observed. This could support the chromatin modulation required for a more regulated proximal TSS deployment of pro-cancer phenotype genes. Metabolic anomalies that drive energy sequestering by cancer cells are near universal to cancers. The absence of these metabolic anomalies in non-cancer cells automatically creates an energy resource bottleneck against stochastic and wasteful TSS deployment. The steady state gene expression profile, as represented by the transcriptomes, can hence be viewed as an outcome of a tug-of-war between high- d TSS deployment and an energy resource curb on it exerted through stochastic deployment of TSSs following the energy-conserving TSS design of the genome. A gradual shift of this balance toward stochastic TSS deployment supported by loss of energy resource curbs is a pervasive feature of cancers. We observed it as a significant difference between heterogeneous cancer or non-cancer sample sets for three independently calculated parameters BCI , $E_{g}$ , and vd , each of which represents the d and TSS deployment frequencies in different functional perspectives. It is no coincidence that the very genes involved in biosynthesis and core features of cancer cell fitness, growth, and proliferation exhibit a selection for energy-conserving TSS deployment. Our report is the first evidence that transcriptional cost mitigation provides a feedback mechanism for genes involved in rapid biosynthetic and proliferative requirements of cancer cells.

A net higher energy cost coefficient of transcription in cancers ensues from the largely stochastic deployment of high- d TSSs and only a selective loss of stochastic TSS deployment for cancer hallmark genes. Our results establish that this is achieved through a combination of transcriptional activity and d of TSSs, which can be defined as a consistent preference for more distal TSSs in cancers. A combination of preferential TSS deployment within genes and their consistent deployment in the cancer sample set would prefer a larger effective d . To reconcile these observations and establish a d dependence of the stochasticity in the two dimensions, an explanation for how non-cancers conduct their transcriptomes is needed. The cancer transcriptomes could then be interpreted in light of the properties of the non-cancer transcriptomes. Our results establish that non-cancer cell and tissue type identities depend on distinct TSS deployment. The cell type-specific TSS deployment is a highly regulated process achieved through differentiation and yields low sample-space stochasticity for *frequently deployed TSSs*. The high sample-space stochasticity in cancers for the *frequently deployed TSSs*, which also have low d TSSs, can be understood as a disregard for the functional identity of the normal precursor cells for any cancer type as long as the cancer-type independent hallmarks of biosynthesis and cell proliferation are supported by energy-conserving transcription. The low TCI deployment of high- d TSSs with low sH in cancers is neither concentrated targeted to genes as per their functions nor seems to be of any adaptive advantage. It just appears to be a leaky deregulated transcription from normally silent TSSs. Thus, the TSS deployment patterns in cancers display a convergent TSS deployment that is poised against the divergent TSS deployment evolved to attain and maintain cellular diversity, as observed in non-cancers.

TSS localization with respect to the start codons has evolved to minimize futile transcription. This is evident in the proximal concentration of TSSs with the strongest mode at d <1 kb; apparently, d is limited by the 5′ UTR length constraints. In the absence of such an evolutionary design, a simple stochastic deployment of TSSs would incur a higher futile transcription and consequently a higher energy and resource cost of transcriptomes. The gene-space entropies are higher in non-cancer cells, indicating that the energy-saving mechanism due to stochastic deployment of proximally concentrated TSSs is sufficient to overcome the regulated tissue-type deployment of >1-kb TSSs. This higher stochasticity in the gene space as an energy-saving mechanism for non-cancer transcriptomes significantly limits E . Cancers maintain a higher gene-space entropy of TSS deployment, which is inherently more expensive. Long-distance transcription entails a higher resource usage, and non-cancer cells exercise a low stochasticity mechanism to deploy them. This energy- and resource-intensive stochastic deployment of TSSs in cancers is likely to derive support from addiction of cancer cells to glucose, glutamine, and an overt preference for biosynthetic pathways, including nucleotide biosynthesis. While this gene function-linked TSS choice and futile transcription appears to be widely applicable as a cancer versus non-cancer differentiator, there are alternate possibilities that could render this phenomenon less universal and vary from one cancer to another. For instance, the observed pattern could be a downstream consequence of *MYC*-driven transcriptional amplification, higher RNA polymerase II activity in transcription-addicted cancer cells, or metabolic reprogramming. Since these mechanisms depend on specific combinations of mutations and epimutations, the observed TSS preferences in cancers could partially be affected by the genetic and epigenetic states of cancers and not manifest as a universal cancer hallmark.

The futile transcription distance affected by the choice of TSSs is not functionally irrelevant. The stochastic deployment of distal TSSs in cancers selectively spares genes that code for some of the most fundamental features of cancer cells. These include cell proliferation, DNA and chromatin organization, and RNA processing (including transcription and splicing). This discriminative transcription of cancer phenotype-supporting genes preferentially from proximal TSSs cannot be achieved merely through stochastic deployments. On the contrary, these genes present a case for a selective and deterministic disallowance for stochastic distal TSS deployment. Apparently, two different spheres of stochasticity affect TSS deployments in a representative cancer cell: a gene function-agnostic, very high stochasticity for distal TSSs, and a gene function-sensitive, low stochasticity TSS deployment for proximal TSSs. Such a duality of stochastic TSS deployment would require spatiotemporal segregation of TSSs in the nuclear space, and colocalizing TSSs would share similar transcriptional resources with similar stochastic or regulated deployment outcomes. A d -dependent TSS colocalization is not established in cancers. However, if similar transcriptional activity outcomes depend on TSS colocalization, then it follows from our analyses that non-cancers and cancers have fundamentally different TSS colocalization patterns. Non-cancers would maintain a chromatin topology, and hence, TSS colocalization is necessary for the functional outcome of the specific cell type. Cancers, conversely, abandon the cell type-specific regulated TSS colocalization patterns and render the chromatin topology stochastic. In evolving tumor cell masses, the cells that maintain and enrich chromatin topologies compatible with regulated proximal TSS deployments for cell proliferation and biosynthesis genes save the energy cost coefficients of transcription and get selected in an energy-constrained tumor environment. Such a selection of cancer phenotype-supporting TSS colocalization would follow as a secondary epigenetic outcome due to a primary change in the TSS deployment landscapes of chromatin regulatory genes. This argument derives support from the observations that the GO BP term chromatin regulation (highly enriched in the cancer-specific changes in vd , $E_{g}$ , gH , sH , cH , and vd/cH ) co-occur with DNA/RNA binding as the only GO MF terms.

The increased stochasticity in TSS deployment allows a larger set of start site options to cancer cells. Rapidly dividing cancer cells benefit from an energy-conserving proximal TSS deployment for genes with necessary high expression levels (biosynthesis, proliferation, transcription, and replication). Such a possibility, supported by our findings, would propel the selection of cells with stochastic TSS deployment in a constantly evolving tumor mass. Increased stochasticity of TSS deployment would overall conserve E by passively increasing the utilization of proximal TSSs, which are far more numerous in the genome. In addition, however, the enhanced stochasticity of TSS deployment could be an epiphenomenon in cancers due to underlying regulation of transcriptional control mechanisms at multiple levels.

A combined interpretation of our findings suggests that there is a feedback between the transcriptional resource cost for a gene and the fitness advantage for a cancer cell accorded by the function of that gene upon its expression. This feedback selects against a chancy and high-cost transcription from distal TSSs for those genes that are fundamental drivers of cancer cell fitness and selection in an evolving tumor. Additional support for this argument comes from the observation that the functional categories of genes for which there is a stochastic distal TSS preference in cancers do not support the cancer phenotype, unlike those with a proximal TSS preference. The parameter vd/cH combines two critical components: the effective d for a gene regardless of the stochasticity or regulation underlying its manifestation and compound entropy that represents the entire measurable stochasticity of TSS deployment for each gene across a sample set. d is a fixed parameter, whereas TCI reduces the sample space to 1. As such, by themselves, the parameters d and TCI represent components of the overall energy cost coefficients ( vd and $E_{g}$ ) without taking into account the stochasticity associated with TSS deployments. d being identical to all samples for any given genome can only be deployed differently in different samples with different E outcomes. cH combined the stochasticity in two independent dimensions (samples and genes) using which the stochasticity underlying any vd could be estimated. These results established that cH is a gene function-relevant estimator of the stochasticity with which TSSs at different d are deployed relative to each other in heterogeneous pools of cancers or non-cancers. By themselves, neither of these two parameters ( vd or cH ) are expected to be concentrated at genes with specific cancer-relevant functions. It follows then that a combination of vd and cH is also unlikely to be a gene-function-relevant parameter. Unexpectedly, however, vd/cH combines these two parameters in a way that it allows cancer versus non-cancer distinction. vd/cH differences between *C* and *N* show that the effective d of transcription is achieved through a limited stochastic TSS deployment that is calibrated by the functional outcome of the gene in question. Of all the parameters tested ( gH , sH , cH , vd , and vd/cH ), the most significant enrichment of biosynthesis and cell proliferation genes was obtained by this compound parameter of vd/cH . Thus, at these cancer hallmark phenotypes driving genes, the effective d minimization and overall stochasticity of TSS deployment are not independent. These two parameters cooperate such that high cH drives low vd for cancer phenotype-driving genes at a higher energy cost coefficient. As a corollary, this less stochastic deployment of high- d TSSs spares cancer hallmark driving genes at a high E with apparently much-attenuated functional feedback on TSS deployment. Finally, the estimated cost of transcription ( COST ) allows a relatable measure of energy cost differences between *C* and *N* in terms of hydrolyzed phosphate bonds and differentiates *C* from *N*.

Thus, the energetics of transcription contributes to cancer-supporting gene expression patterns through feedback between transcribed futile distance and the corresponding gene functions. The TSS deployment in non-cancer cells follows an energy-conserving design, and cancer cells universally subvert it to the extent it benefits their selection in the process of tumor evolution.

## Data Availability

The datasets presented in this study can be found in online repositories. The names of the repository/repositories and accession number(s) can be found in the article/Supplementary Material.

## References

[B1] Adashek J. J. Kato S. Parulkar R. Szeto C. W. Sanborn J. Z. Vaske C. J. (2020). Transcriptomic silencing as a potential mechanism of treatment resistance. JCI Insight 5 (11), 134824. 10.1172/jci.insight.134824 32493840 PMC7308055

[B2] Arner E. Daub C. O. Vitting-Seerup K. Andersson R. Lilje B. Drabløs F. (2015). Transcribed enhancers lead waves of coordinated transcription in transitioning mammalian cells. Science 347 (6225), 1010–1014. 10.1126/science.1259418 25678556 PMC4681433

[B3] Auslander N. Wolf Y. I. Koonin E. V. (2019). *In silico* learning of tumor evolution through mutational time series. Proc. Natl. Acad. Sci. U. S. A. 116 (19), 9501–9510. 10.1073/pnas.1901695116 31015295 PMC6510994

[B4] Berardi M. J. Fantin V. R. (2011). Survival of the fittest: metabolic adaptations in cancer. Curr. Opin. Genet. Dev. 21 (1), 59–66. 10.1016/j.gde.2010.10.001 21112206

[B5] Bradner J. E. Hnisz D. Young R. A. (2017). Transcriptional addiction in cancer. Cell. 168 (4), 629–643. 10.1016/j.cell.2016.12.013 28187285 PMC5308559

[B6] Cao Z. Filatova T. Oyarzún D. A. Grima R. (2020). A stochastic model of gene expression with polymerase recruitment and pause release. Biophys. J. 119 (5), 1002–1014. 10.1016/j.bpj.2020.07.020 32814062 PMC7474183

[B7] Carninci P. Kasukawa T. Katayama S. Gough J. Frith M. C. Maeda N. (2005). The transcriptional landscape of the mammalian genome. Science 309 (5740), 1559–1563. 10.1126/science.1112014 16141072

[B8] Cluntun A. A. Lukey M. J. Cerione R. A. Locasale J. W. (2017). Glutamine metabolism in cancer: understanding the heterogeneity. Trends Cancer 3 (3), 169–180. 10.1016/j.trecan.2017.01.005 28393116 PMC5383348

[B9] Cottrell K. A. Chaudhari H. G. Cohen B. A. Djuranovic S. (2018). PTRE-seq reveals mechanism and interactions of RNA binding proteins and miRNAs. Nat. Commun. 9 (1), 301. 10.1038/s41467-017-02745-0 29352242 PMC5775260

[B10] Dawson M. A. Kouzarides T. (2012). Cancer epigenetics: from mechanism to therapy. Cell. 150 (1), 12–27. 10.1016/j.cell.2012.06.013 22770212

[B11] de Klerk E. ’t Hoen P. A. C. (2015). Alternative mRNA transcription, processing, and translation: insights from RNA sequencing. Trends Genet. 31 (3), 128–139. 10.1016/j.tig.2015.01.001 25648499

[B12] Demircioğlu D. Cukuroglu E. Kindermans M. Nandi T. Calabrese C. Fonseca N. A. (2019). A pan-cancer transcriptome analysis reveals pervasive regulation through alternative promoters. Cell. 178 (6), 1465–1477.e17. 10.1016/j.cell.2019.08.018 31491388

[B13] Deng Q. Ramsköld D. Reinius B. Sandberg R. (2014). Single-cell RNA-seq reveals dynamic, random monoallelic gene expression in mammalian cells. Science 343 (6167), 193–196. 10.1126/science.1245316 24408435

[B14] Dobosz P. Stempor P. A. Ramírez Moreno M. Bulgakova N. A. (2022). Transcriptional and post-transcriptional regulation of checkpoint genes on the tumour side of the immunological synapse. Hered. (Edinb.) 129 (1), 64–74. 10.1038/s41437-022-00533-1 35459932 PMC9273643

[B64] Eden E. Navon R. Steinfeld I. Lipson D. Yakhini Z. (2009). GOrilla: a tool for discovery and visualization of enriched GO terms in ranked gene lists. BMC Bioinfo. 10, 48. 19192299 10.1186/1471-2105-10-48 PMC2644678

[B65] Eden E. Lipson D. Yogev Z. (2007). Discovering motifs in ranked lists of DNA sequences. PLoS Comput. Biol. 3 (3), e39. 17381235 10.1371/journal.pcbi.0030039 PMC1829477

[B15] ENCODE Project Consortium Moore J. E. Purcaro M. J. Pratt H. E. Epstein C. B. Shoresh N. Adrian J. (2020). Expanded encyclopaedias of DNA elements in the human and mouse genomes. Nature 583 (7818), 699–710. 10.1038/s41586-020-2493-4 32728249 PMC7410828

[B16] FANTOM Consortium and the RIKEN PMI and CLST (DGT); Forrest A. R. R. Kawaji H. Rehli M. Baillie J. K. de Hoon M. J. L. Haberle V. (2014). A promoter-level mammalian expression atlas. Nature 507 (7493), 462–470. 10.1038/nature13182 24670764 PMC4529748

[B17] Frankish A. Carbonell-Sala S. Diekhans M. Jungreis I. Loveland J. E. Mudge J. M. (2023). GENCODE: reference annotation for the human and mouse genomes in 2023. Nucleic Acids Res. 51 (D1), D942–D949. 10.1093/nar/gkac1071 36420896 PMC9825462

[B18] Friedrich D. Friedel L. Finzel A. Herrmann A. Preibisch S. Loewer A. (2019). Stochastic transcription in the p53-mediated response to DNA damage is modulated by burst frequency. Mol. Syst. Biol. 15 (12), e9068. 10.15252/msb.20199068 31885199 PMC6886302

[B19] Gao Y. Teschendorff A. E. (2017). Epigenetic and genetic deregulation in cancer target distinct signaling pathway domains. Nucleic Acids Res. 45 (2), 583–596. 10.1093/nar/gkw1100 27899617 PMC5314760

[B20] Gonzalez-Sandoval A. Gasser S. M. (2016). On TADs and LADs: spatial control over gene expression. Trends Genet. 32 (8), 485–495. 10.1016/j.tig.2016.05.004 27312344

[B21] Hanahan D. Weinberg R. A. (2000). The hallmarks of cancer. Cell. 100 (1), 57–70. 10.1016/s0092-8674(00)81683-9 10647931

[B22] Hanahan D. Weinberg R. A. (2011). Hallmarks of cancer: the next generation. Cell. 144 (5), 646–674. 10.1016/j.cell.2011.02.013 21376230

[B23] Harris H. L. Gu H. Olshansky M. Wang A. Farabella I. Eliaz Y. (2023). Chromatin alternates between A and B compartments at kilobase scale for subgenic organization. Nat. Commun. 14 (1), 3303. 10.1038/s41467-023-38429-1 37280210 PMC10244318

[B24] Hashimoto K. Suzuki A. M. Dos Santos A. Desterke C. Collino A. Ghisletti S. (2015). CAGE profiling of ncRNAs in hepatocellular carcinoma reveals widespread activation of retroviral LTR promoters in virus-induced tumors. Genome Res. 25 (12), 1812–1824. 10.1101/gr.191031.115 26510915 PMC4665003

[B25] Hirabayashi S. Bhagat S. Matsuki Y. Takegami Y. Uehata T. Kanemaru A. (2019). NET-CAGE characterizes the dynamics and topology of human transcribed cis-regulatory elements. Nat. Genet. 51 (9), 1369–1379. 10.1038/s41588-019-0485-9 31477927

[B26] Huang K. K. Huang J. Wu J. K. L. Lee M. Tay S. T. Kumar V. (2021). Long-read transcriptome sequencing reveals abundant promoter diversity in distinct molecular subtypes of gastric cancer. Genome Biol. 22 (1), 44. 10.1186/s13059-021-02261-x 33482911 PMC7821541

[B27] Jha A. Quesnel-Vallières M. Wang D. Thomas-Tikhonenko A. Lynch K. W. Barash Y. (2022). Identifying common transcriptome signatures of cancer by interpreting deep learning models. Genome Biol. 23 (1), 117. 10.1186/s13059-022-02681-3 35581644 PMC9112525

[B28] Jones P. A. Issa J.-P. J. Baylin S. (2016). Targeting the cancer epigenome for therapy. Nat. Rev. Genet. 17 (10), 630–641. 10.1038/nrg.2016.93 27629931

[B29] Kagohara L. T. Stein-O'Brien G. L. Kelley D. Flam E. Wick H. C. Danilova L. V. (2018). Epigenetic regulation of gene expression in cancer: techniques, resources and analysis. Brief. Funct. Genomics 17 (1), 49–63. 10.1093/bfgp/elx018 28968850 PMC5860551

[B30] Kanamori-Katayama M. Itoh M. Kawaji H. Lassmann T. Katayama S. Kojima M. (2011). Unamplified cap analysis of gene expression on a single-molecule sequencer. Genome Res. 21 (7), 1150–1159. 10.1101/gr.115469.110 21596820 PMC3129257

[B31] Karlsson K. Lönnerberg P. Linnarsson S. (2017). Alternative TSSs are co-regulated in single cells in the mouse brain. Mol. Syst. Biol. 13 (5), 930. 10.15252/msb.20167374 28495919 PMC5448164

[B32] Kepler T. B. Elston T. C. (2001). Stochasticity in transcriptional regulation: origins, consequences, and mathematical representations. Biophys. J. 81 (6), 3116–3136. 10.1016/S0006-3495(01)75949-8 11720979 PMC1301773

[B33] Kim T. Croce C. M. (2023). MicroRNA: trends in clinical trials of cancer diagnosis and therapy strategies. Exp. Mol. Med. 55 (7), 1314–1321. 10.1038/s12276-023-01050-9 37430087 PMC10394030

[B34] Kutchko K. M. Sanders W. Ziehr B. Phillips G. Solem A. Halvorsen M. (2015). Multiple conformations are a conserved and regulatory feature of the RB1 5’ UTR. RNA 21 (7), 1274–1285. 10.1261/rna.049221.114 25999316 PMC4478346

[B35] Lee T. I. Young R. A. (2013). Transcriptional regulation and its misregulation in disease. Cell. 152 (6), 1237–1251. 10.1016/j.cell.2013.02.014 23498934 PMC3640494

[B36] Lipniacki T. Paszek P. Marciniak-Czochra A. Brasier A. R. Kimmel M. (2006). Transcriptional stochasticity in gene expression. J. Theor. Biol. 238 (2), 348–367. 10.1016/j.jtbi.2005.05.032 16039671

[B37] Lynch M. Marinov G. K. (2015). The bioenergetic costs of a gene. Proc. Natl. Acad. Sci. U. S. A. 112 (51), 15690–15695. 10.1073/pnas.1514974112 26575626 PMC4697398

[B38] Mullen N. J. Singh P. K. (2023). Nucleotide metabolism: a pan-cancer metabolic dependency. Nat. Rev. Cancer 23 (5), 275–294. 10.1038/s41568-023-00557-7 36973407 PMC10041518

[B39] Makalowska I. Lin C.-F. Makalowski W. (2005). Overlapping genes in vertebrate genomes. Comput. Biol. Chem. 29 (1), 1–12. 10.1016/j.compbiolchem.2004.12.006 15680581

[B40] Marziali F. Dizanzo M. P. Cavatorta A. L. Gardiol D. (2019). Differential expression of DLG1 as a common trait in different human diseases: an encouraging issue in molecular pathology. Biol. Chem. 400 (6), 699–710. 10.1515/hsz-2018-0350 30517074

[B41] McGillivray P. Ault R. Pawashe M. Kitchen R. Balasubramanian S. Gerstein M. (2018). A comprehensive catalog of predicted functional upstream open reading frames in humans. Nucleic Acids Res. 46 (7), 3326–3338. 10.1093/nar/gky188 29562350 PMC6283423

[B42] Mularoni L. Sabarinathan R. Deu-Pons J. Gonzalez-Perez A. López-Bigas N. (2016). OncodriveFML: a general framework to identify coding and non-coding regions with cancer driver mutations. Genome Biol. 17 (1), 128. 10.1186/s13059-016-0994-0 27311963 PMC4910259

[B43] Nepal C. Andersen J. B. (2023). Alternative promoters in CpG depleted regions are prevalently associated with epigenetic misregulation of liver cancer transcriptomes. Nat. Commun. 14 (1), 2712. 10.1038/s41467-023-38272-4 37169774 PMC10175279

[B44] Ni T. Corcoran D. L. Rach E. A. Song S. Spana E. P. Gao Y. (2010). A paired-end sequencing strategy to map the complex landscape of transcription initiation. Nat. Methods 7 (7), 521–527. 10.1038/nmeth.1464 20495556 PMC3197272

[B45] Noguchi S. Arakawa T. Fukuda S. Furuno M. Hasegawa A. Hori F. (2017). FANTOM5 CAGE profiles of human and mouse samples. Sci. Data 4 (1), 170112. 10.1038/sdata.2017.112 28850106 PMC5574368

[B47] Pollex T. Marco-Ferreres R. Ciglar L. Ghavi-Helm Y. Rabinowitz A. Viales R. R. (2024). Chromatin gene-gene loops support the cross-regulation of genes with related function. Mol. Cell. 84 (5), 822–838.e8. 10.1016/j.molcel.2023.12.023 38157845

[B48] Preikšaitienė E. Kučinskas V. (2016). Genomic control process: development and evolution. Eur. J. Hum. Genet. 24(7):1093. 10.1038/ejhg.2015.245

[B49] Quek L.-E. van Geldermalsen M. Guan Y. F. Wahi K. Mayoh C. Balaban S. (2022). Glutamine addiction promotes glucose oxidation in triple-negative breast cancer. Oncogene 41 (34), 4066–4078. 10.1038/s41388-022-02408-5 35851845 PMC9391225

[B50] Raj A. van Oudenaarden A. (2008). Nature, nurture, or chance: stochastic gene expression and its consequences. Cell. 135 (2), 216–226. 10.1016/j.cell.2008.09.050 18957198 PMC3118044

[B51] Raj A. Peskin C. S. Tranchina D. Vargas D. Y. Tyagi S. (2006). Stochastic mRNA synthesis in mammalian cells. PLoS Biol. 4 (10), e309. 10.1371/journal.pbio.0040309 17048983 PMC1563489

[B52] Rapin N. Bagger F. O. Jendholm J. Mora-Jensen H. Krogh A. Kohlmann A. (2014). Comparing cancer vs normal gene expression profiles identifies new disease entities and common transcriptional programs in AML patients. Blood 123 (6), 894–904. 10.1182/blood-2013-02-485771 24363398

[B53] Renz P. F. Valdivia-Francia F. Sendoel A. (2020). Some like it translated: small ORFs in the 5’UTR. Exp. Cell. Res. 396 (1), 112229. 10.1016/j.yexcr.2020.112229 32818479

[B54] Shiraki T. Kondo S. Katayama S. Waki K. Kasukawa T. Kawaji H. (2003). Cap analysis gene expression for high-throughput analysis of transcriptional starting point and identification of promoter usage. Proc. Natl. Acad. Sci. U. S. A. 100 (26), 15776–15781. 10.1073/pnas.2136655100 14663149 PMC307644

[B55] Su K. Yu Q. Shen R. Sun S. Y. Moreno C. S. Li X. (2021). Pan-cancer analysis of pathway-based gene expression pattern at the individual level reveals biomarkers of clinical prognosis. Cell. Rep. Methods 1 (4), 100050. 10.1016/j.crmeth.2021.100050 34671755 PMC8525796

[B56] Swanton C. Bernard E. Abbosh C. André F. Auwerx J. Balmain A. (2024). Embracing cancer complexity: hallmarks of systemic disease. Cell. 187 (7), 1589–1616. 10.1016/j.cell.2024.02.009 38552609 PMC12077170

[B57] Tahiri A. Leivonen S. K. Lüders T. Steinfeld I. Ragle Aure M. Geisler J. (2014). Deregulation of cancer-related miRNAs is a common event in both benign and malignant human breast tumors. Carcinogenesis 35 (1), 76–85. 10.1093/carcin/bgt333 24104550

[B58] Wang X. Hou J. Quedenau C. Chen W. (2016). Pervasive isoform-specific translational regulation *via* alternative transcription start sites in mammals. Mol. Syst. Biol. 12 (7), 875. 10.15252/msb.20166941 27430939 PMC4965872

[B59] Weinberg M. S. Morris K. V. (2016). Transcriptional gene silencing in humans. Nucleic Acids Res. 44 (14), 6505–6517. 10.1093/nar/gkw139 27060137 PMC5001580

[B60] Wise D. R. Thompson C. B. (2010). Glutamine addiction: a new therapeutic target in cancer. Trends biochem. Sci. 35 (8), 427–433. 10.1016/j.tibs.2010.05.003 20570523 PMC2917518

[B61] Yang Z. Jones A. Widschwendter M. Teschendorff A. E. (2015). An integrative pan-cancer-wide analysis of epigenetic enzymes reveals universal patterns of epigenomic deregulation in cancer. Genome Biol. 16 (1), 140. 10.1186/s13059-015-0699-9 26169266 PMC4501092

[B62] You J. S. Jones P. A. (2012). Cancer genetics and epigenetics: two sides of the same coin? Cancer Cell. 22 (1), 9–20. 10.1016/j.ccr.2012.06.008 22789535 PMC3396881

[B63] Zhao Z. Chen Y. Zou X. Lin L. Zhou X. Cheng X. (2024). Pan-cancer transcriptome analysis reveals widespread regulation through alternative tandem transcription initiation. Sci. Adv. 10 (28), eadl5606. 10.1126/sciadv.adl5606 38985880 PMC11235174

