# Supplementary material for: Gene functions determine stochastic or adaptive futile transcription in cancers through deregulated start site deployments
Source: Front Bioinform. 2026 Oct 14;6:1883461. doi: 10.3389/fbinf.2026.1883461 (PMC13713664; doi:10.3389/fbinf.2026.1883461)
Supplement: Supplementary file 1 [file DataSheet1.pdf]

Supplementary Material

1 Supplementary Figures

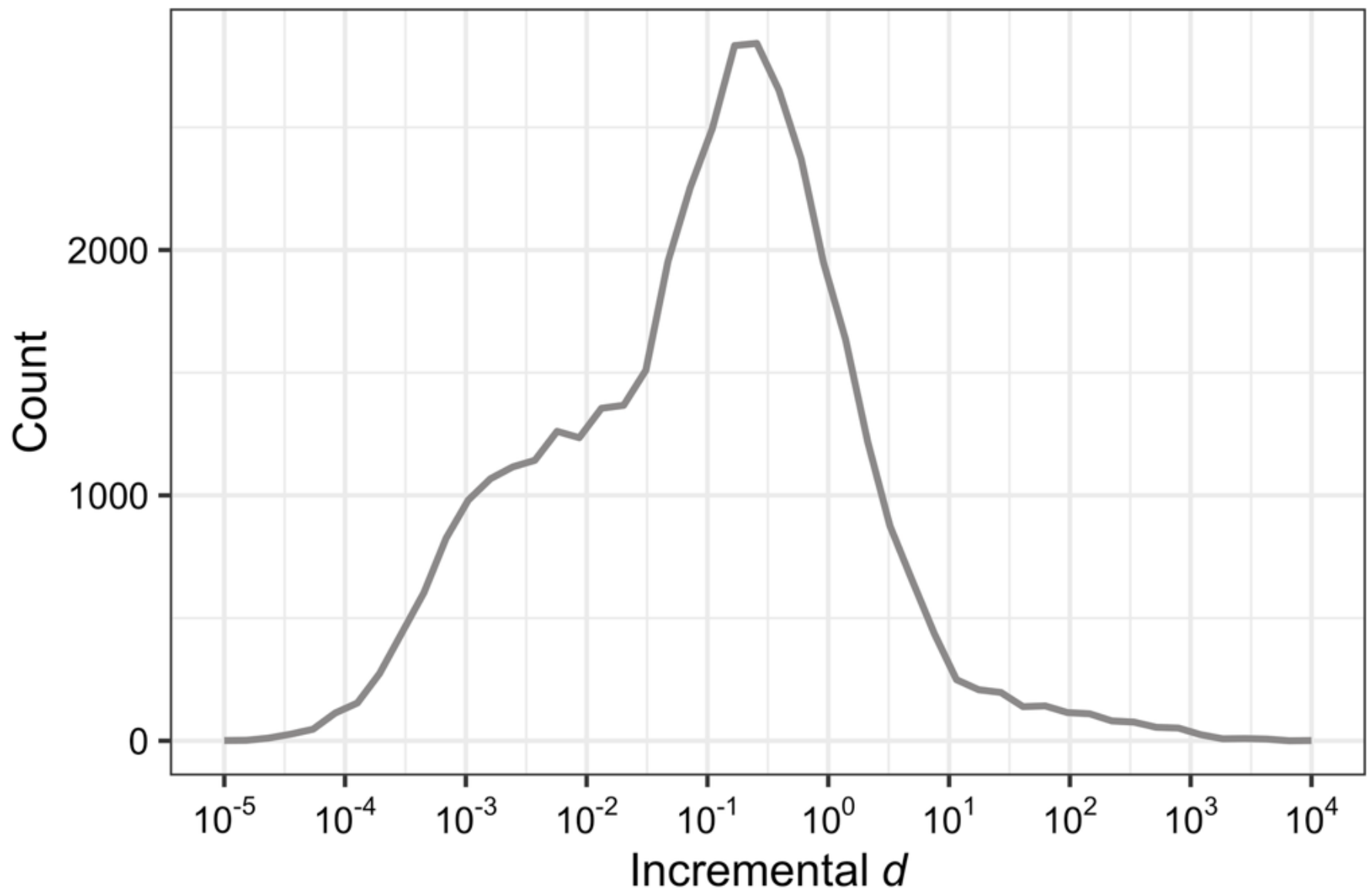

Supplementary Figure 1. Incremental d profiles, calculated gene–wise as ratio of d difference between two TSSs and the d of the proximal TSS in the pair, shows that there is a selection for proximal concentration of TSSs: The frequency distribution shows that the majority of TSSs are at a d which is up to three orders of magnitude (region between 10^-3 and 10^0 on the X-axis) lower than the d expected in the absence of a selection pressure favoring reduction of d.

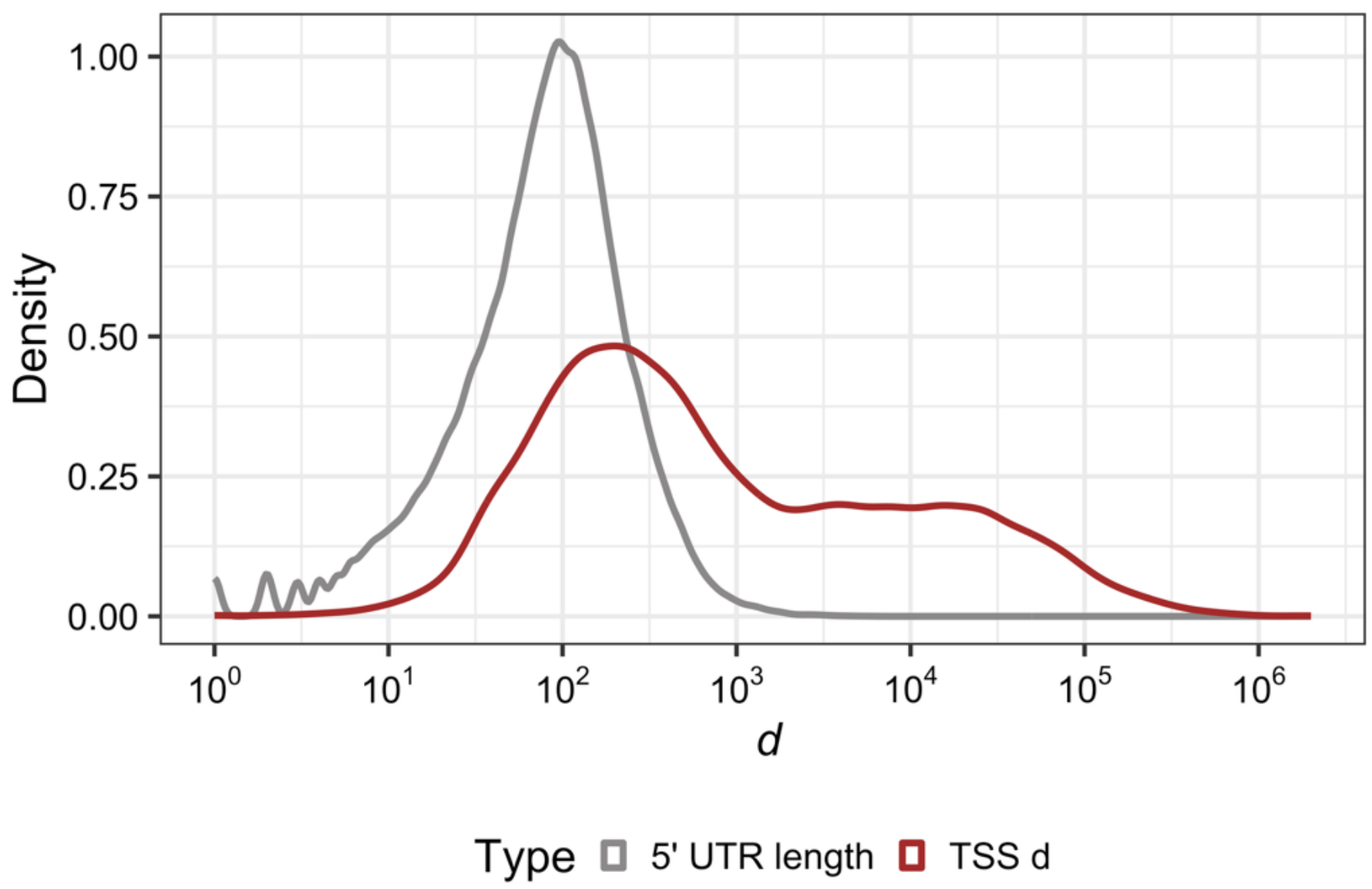

Supplementary Figure 2. Density distribution of 5′ UTR lengths (grey) obtained from GENCODE and TSS *d*–profiles (red) calculated *de novo*: Expectedly, the TSS d distribution shows lengths larger than or equal to the 5′ UTR lengths.

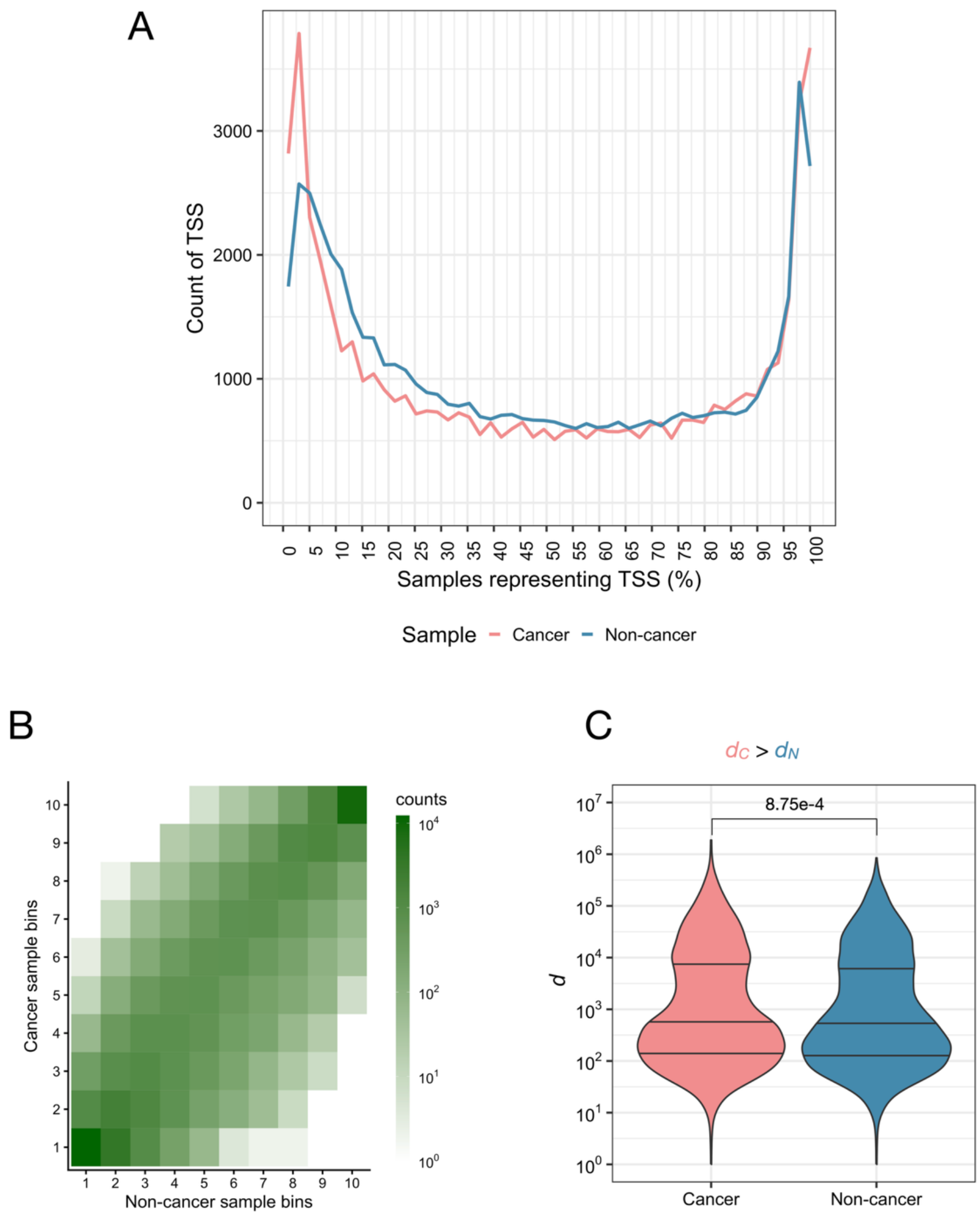

Supplementary Figure 3. TSS counts represented in cancers and non-cancers: (A) TSS representation profiles in cancer (pink) and non–cancer (blue) sample sets highlighting extremes of TSS detection patterns at the two ends of the X–axis. The sample–type restricted TSSs are present in a smaller proportion of the sample set (the left end of the X–axis) whereas the commonly detected TSSs in cancers as well as non–cancers are present in a larger proportion of the sample set (the right end of the X–axis). The two datasets are plotted without any pairing along the X–axis. **(B)** A correlation between the sample count bins (non–cancer on the X–axis and cancer on the Y–axis) in which a TSS occurs shows that the majority of TSSs have similar sample representation. The X and Y axes values are multipliers of the counts depicted in the color bar to the right. **(C)** TSSs that are detected in a sample–type exclusive manner (cancer exclusives in pink and non–cancer exclusives in blue) have a significantly different d. The d of cancer exclusive TSSs (in <5% of non–cancers) are significantly larger than those of the non–cancer exclusive TSSs (in <5% of cancers); (Wilcoxon rank–sum test, p–value = 8.75e-4).

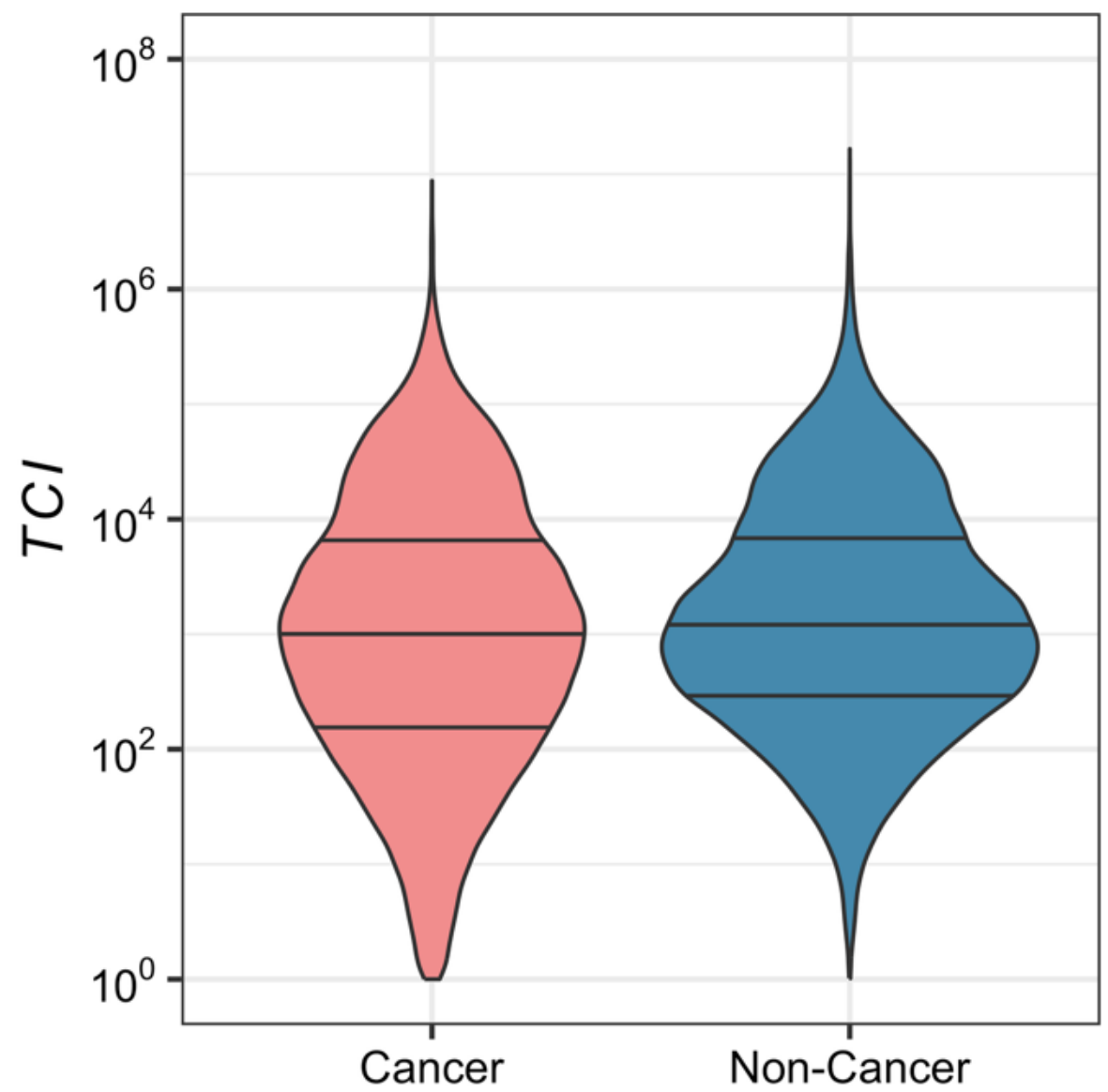

Supplementary Figure 4. $\boldsymbol{TCI}$ **in cancers (**$\boldsymbol{TCI_C}$**, pink) is significantly higher than** $\boldsymbol{TCI}$ **in non–cancers (**$\boldsymbol{TCI_N}$**, blue):** Wilcoxon signed–rank test paired by 55048 TSSs, p-value ≤ 7.846e-3.

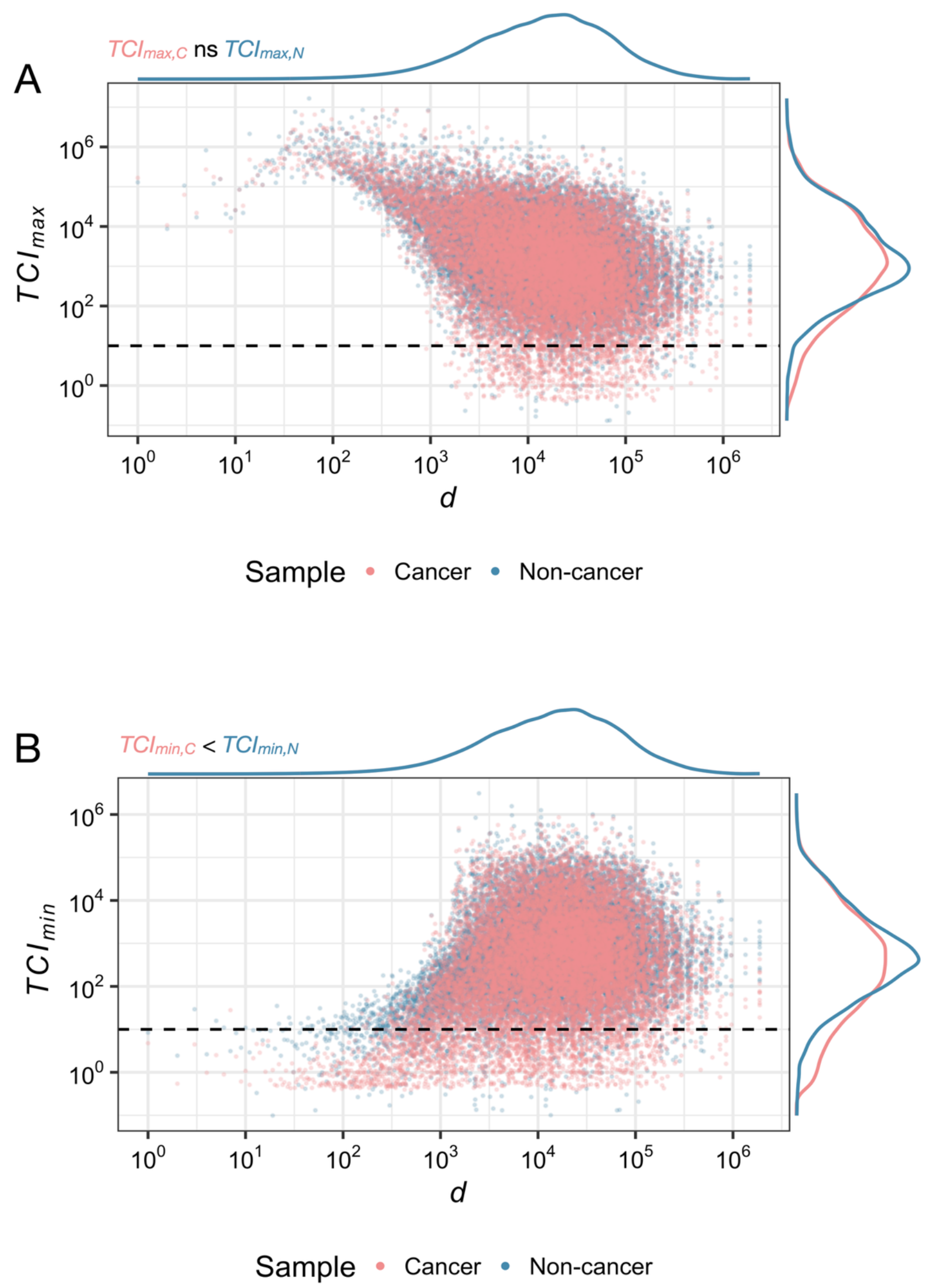

Supplementary Figure 5. Distribution of TSSs with maximum TCI (TCI_{max}) (A) or minimum TCI (TCI_{min}) (B) shows d–dependent differences between C (pink) and N (blue): As d exceeds ~1kb, TCI_{max} and TCI_{min} distributions attain a larger range (between 10^1 and 10^5 on the Y–axes in both the plots) with diminishing differences. Remarkably, even if overall $TCI_C > TCI_N$, the $TCI_{max,C}$ was not significantly higher than $TCI_{max,N}$ (**A**) even if $TCI_{min,C}$ was significantly lower than $TCI_{min,N}$ (Wilcoxon signed–rank test paired by 16458 ds, p–value = 2.85e-82. Pairings of TSSs are only by d, not by gene). For TCI_{max} and TCI_{min} both, a low level of "leaky" transcriptional activity was observed for C (below the dashed horizontal line a Y–axes value of 10^1).

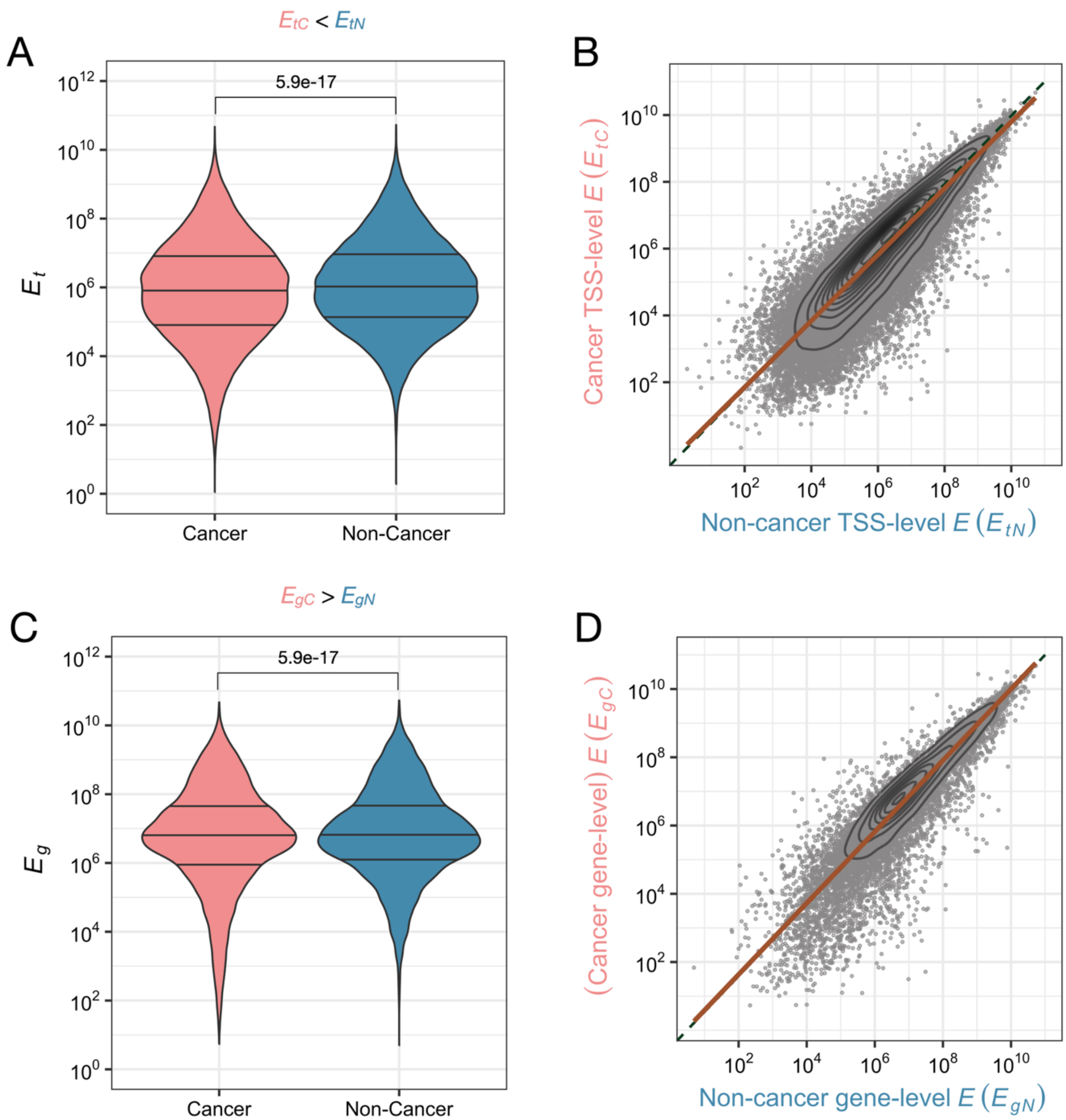

Supplementary Figure 6. TSS–level E (E_t; E_{tN} for non–cancers and E_{tC} for cancers) and gene–level E (E_g; E_{gN} for non–cancers and E_{gC} for cancers) comparisons between cancers and non–cancers reveals that E minimization is a gene function–dependent feature which is compromised in cancers: $E_{tC} < E_{tN}$ without gene–level bundling of TSSs (**A and B**, Wilcoxon signed–rank test paired by 55048 TSSs, p–value = 5.9e-17). This reverses to $E_{gC} > E_{gN}$ after gene–level bundling of TSSs (**C and D**, Wilcoxon signed–rank test paired by 15861 genes, p-value = 1.65e-27).

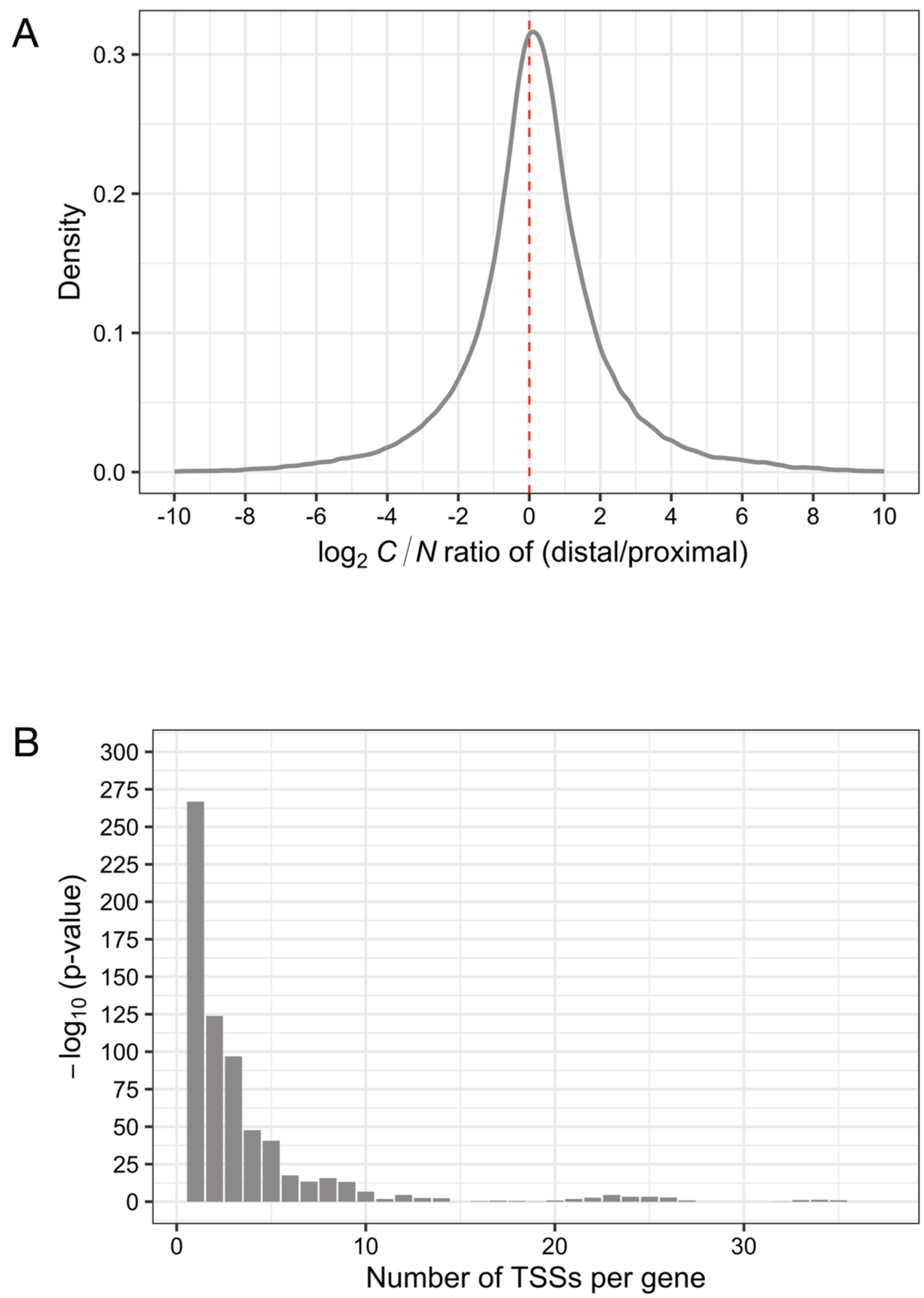

Supplementary Figure 7. A d–independent relative preference for distal TSS deployment for a gene, calculated as TCI ratios of a TSS and its preceding proximal TSS (referred to as distal/proximal ratios), show a bias for distal TSS deployment in cancers: (A) A frequency distribution of the (C/N) of the distal/proximal ratios shows a clear *C*–heavy skew. **(B)** For each pair of consecutive TSSs involved in the calculation of the ratios presented in A, a Wilcoxon signed–rank test comparing $C > N$ was performed and the p–values are presented as $-\log_{10}(p)$ on the Y–axis. The X–axis depicts the number of TSSs per gene. Total number of genes analyzed = 11928. All genes with single TSSs were excluded. No ratios were calculated for the first most proximal TSS for all the genes.

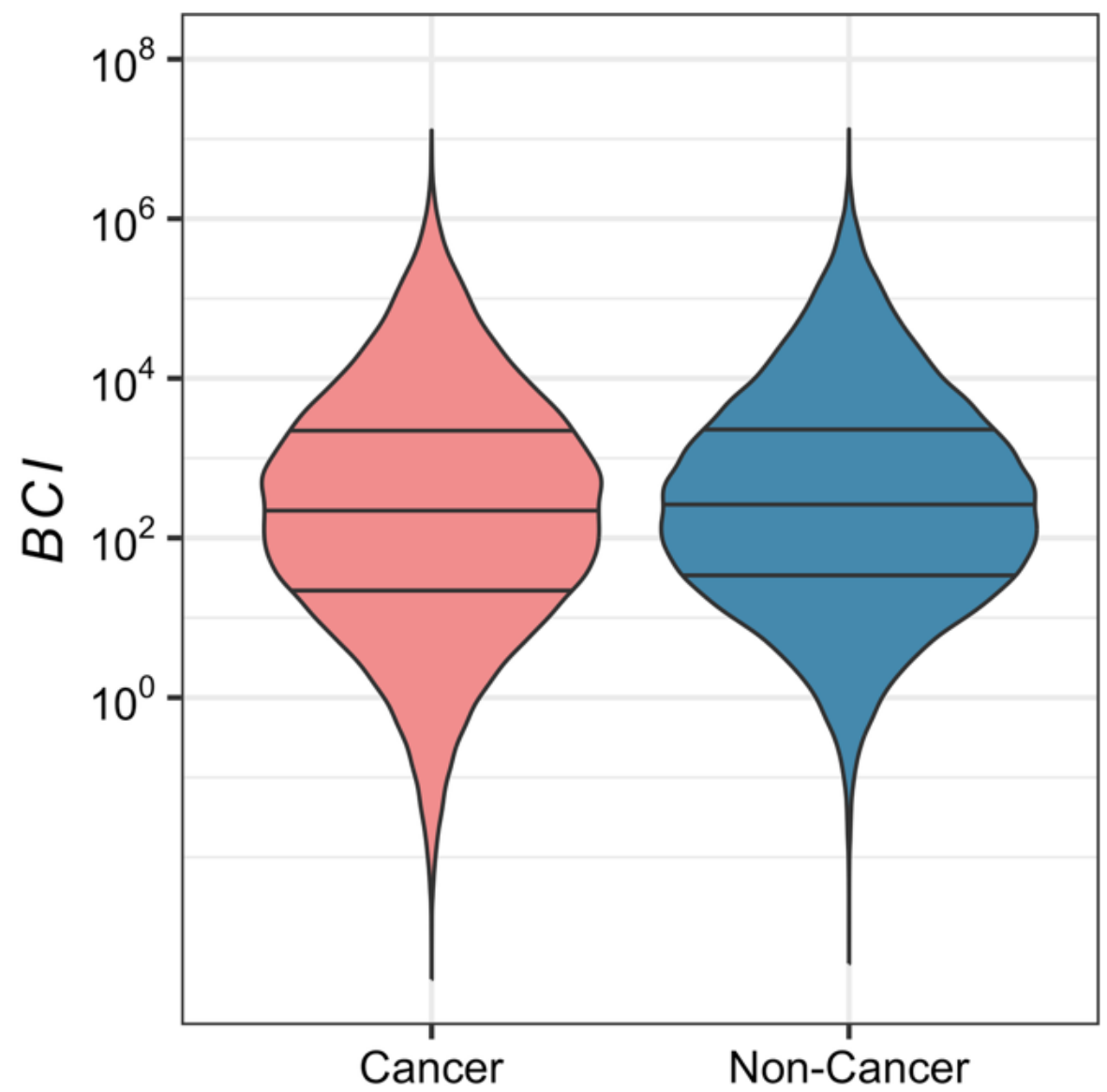

Supplementary Figure 8. BCI in cancers (BCI_C, pink) is significantly higher than BCI in non–cancers (BCI_N, blue); Wilcoxon signed–rank test paired by 55048 TSSs, p–value = 3.67e-37.

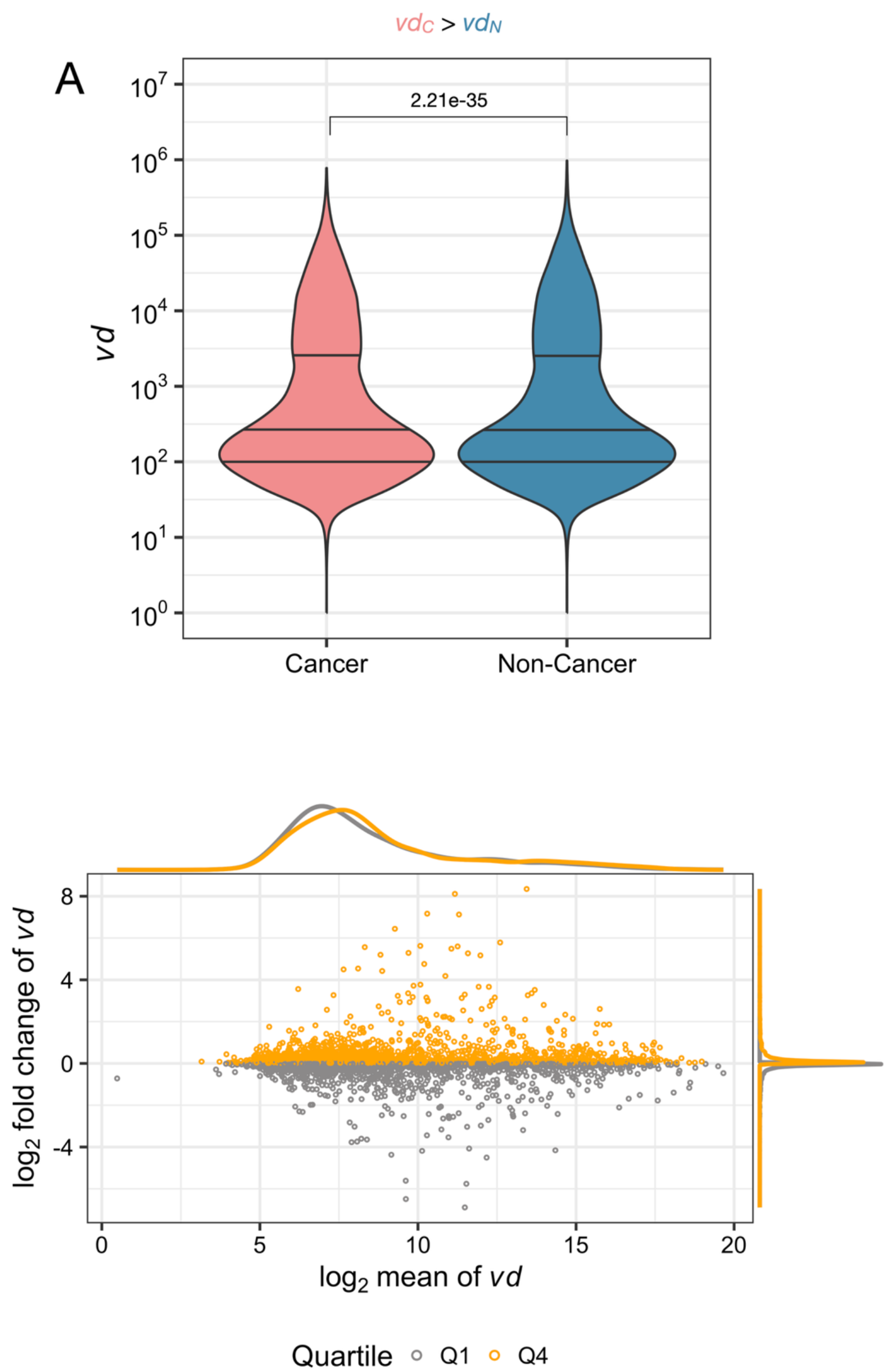

Supplementary Figure 9. TCI-weighted average d (vd) in cancers (pink) is significantly higher than that in non-cancers (blue): (A) Violin plots show vd for cancers (pink) and non-cancers (blue). **(B)** M–A plot of the terminal quartiles of genes sorted by vd ratios calculated as $\frac{vd_C}{vd_N}$ (grey dataset is Q1 with a reduction of vd in cancers and the orange dataset is Q4 with an increase of vd in cancers). All single TSS genes were excluded. These Q1 and Q4 genes were used for functional category enrichment analyses.

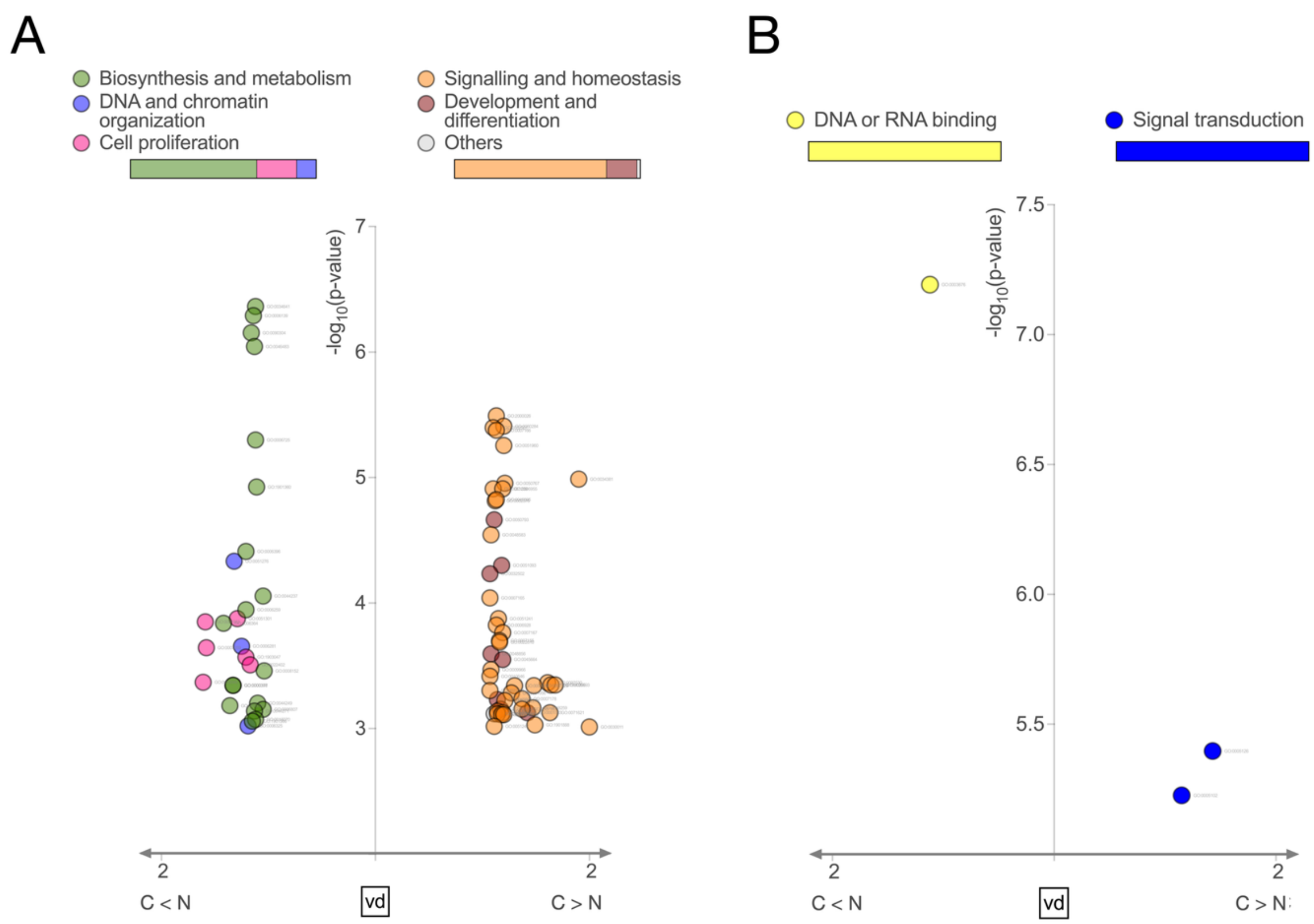

Supplementary Figure 10. vd difference between C and N is linked to gene functions: vd, although overall increased in C over N, is maintained lower in C for cancer hallmark functions, of which biological processes biosynthesis and cell proliferation (**A**), and molecular functions DNA or RNA–binding are noteworthy (**B**). Y–axes show p–value of Fisher exact test for significant enrichment using GOrilla Gene Ontology term enrichment and X–axes show the fold change of vd in C over N. Specific Gene Ontology terms (marked in light grey fonts for every data point and presented in table S14) are merged into color–coded assigned terms presented as legends on the top of the scatter plots.

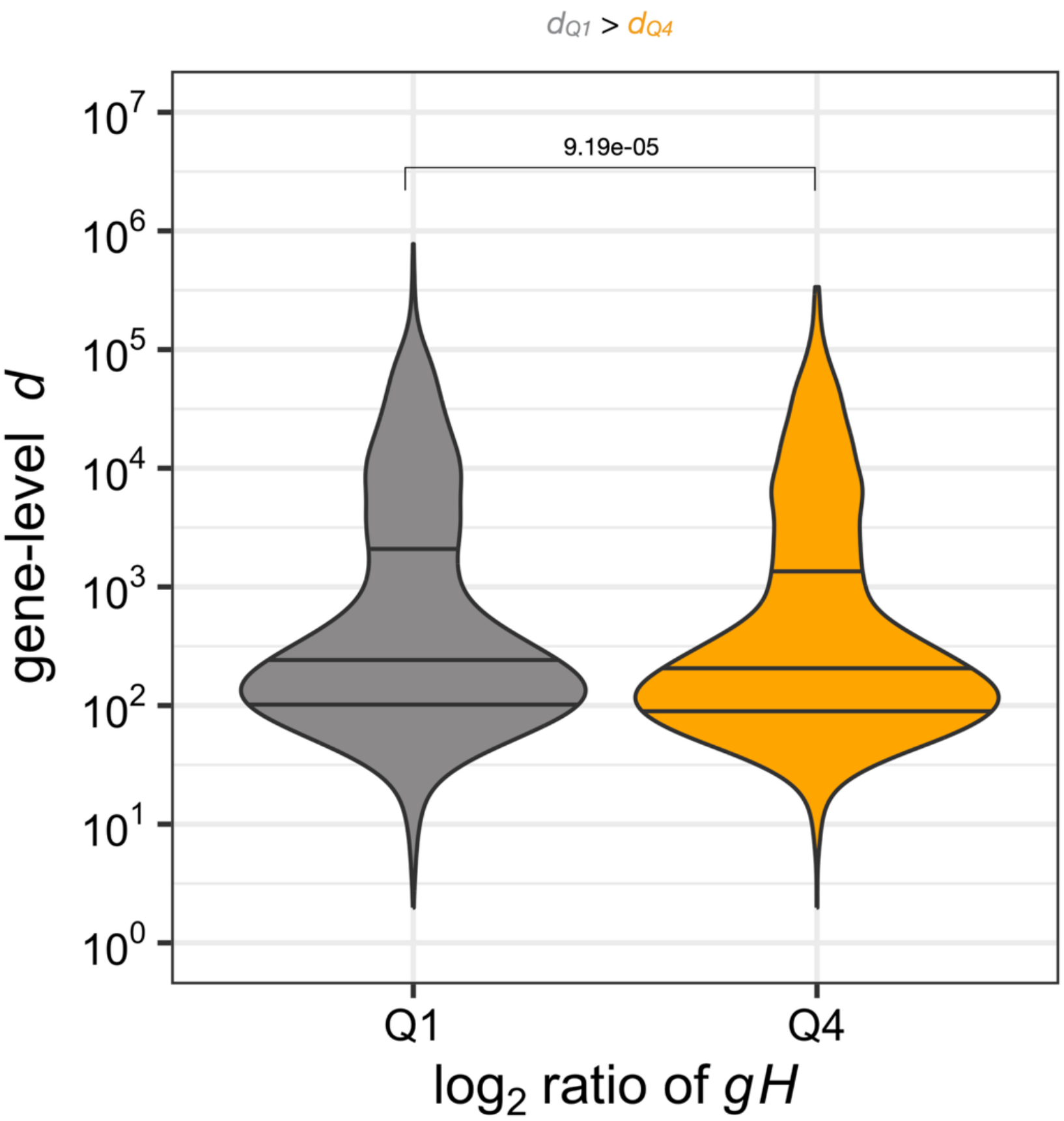

Supplementary Figure 11. Comparison of HMd of the terminal quartiles of genes sorted by gH ratios calculated as gH_C/gH_N (grey dataset is Q1 with a reduction of gH in cancers and yellow dataset is Q4 with an increase of gH in cancers) shows that TSSs with a less stochastic deployment in cancers (lower gene–space entropy of TSS deployment; Q1) have a significantly higher gene–level d; Wilcoxon signed–rank test paired by 11928 genes, p–value = 9.19e-15.

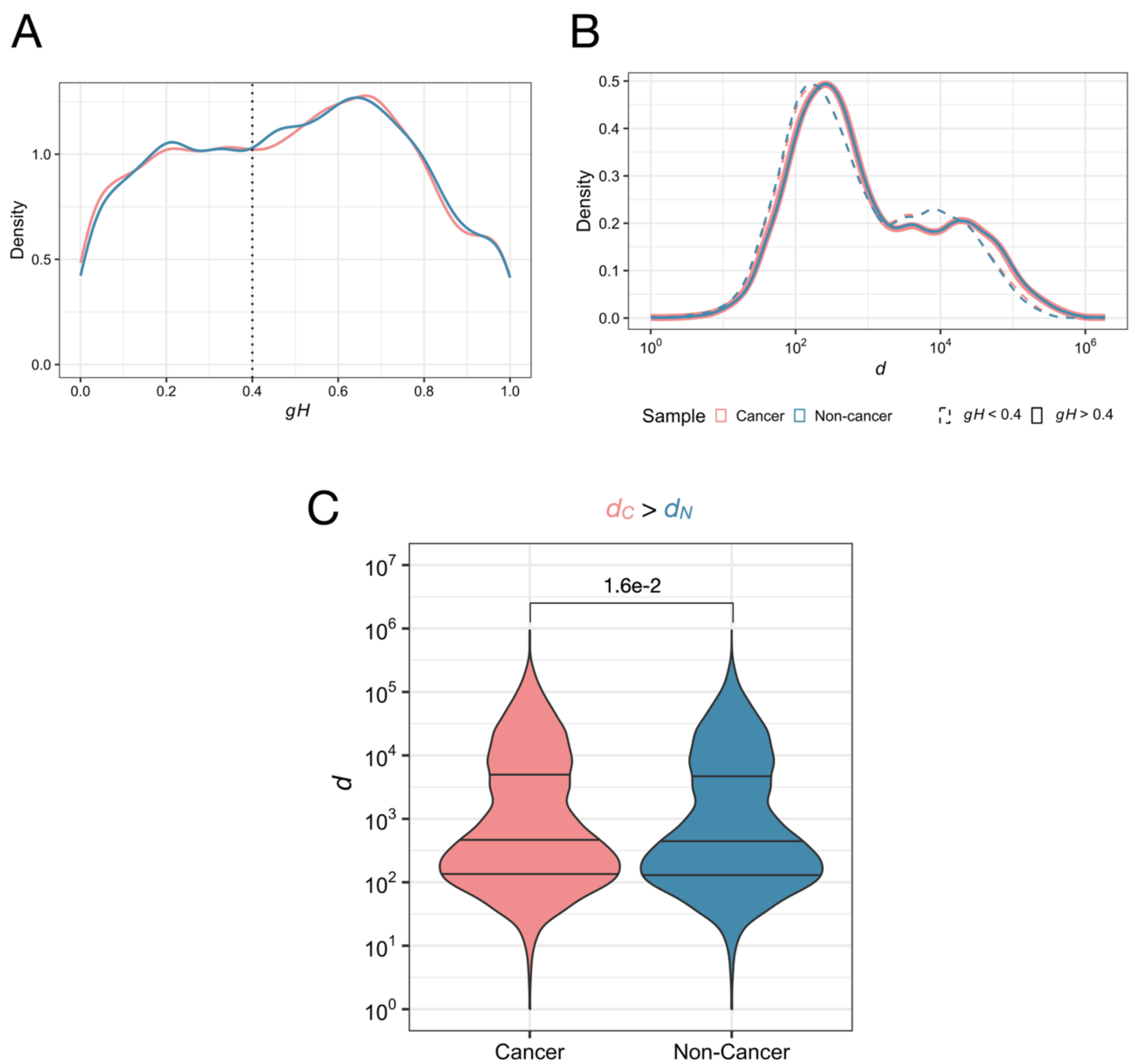

Supplementary Figure 12. A gH threshold of 0.4 classifies genes into two groups: (A) Distribution of gH in N and C with the gH threshold marked as dashed line. **(B)** TSSs belonging to the genes with gH<0.4 also have lower d as compared to the TSSs of genes with gH>0.4 (Wilcoxon rank–sum test, p–value for C = 7.99e-25, and p–value for N = 1.73e-42). **(C)** For the gH<0.4 genes (different sets of genes in C and N) the TSS d profiles are significantly different in cancers and non-cancers (d_C>d_N; Wilcoxon rank–sum test, p–value = 0.016).

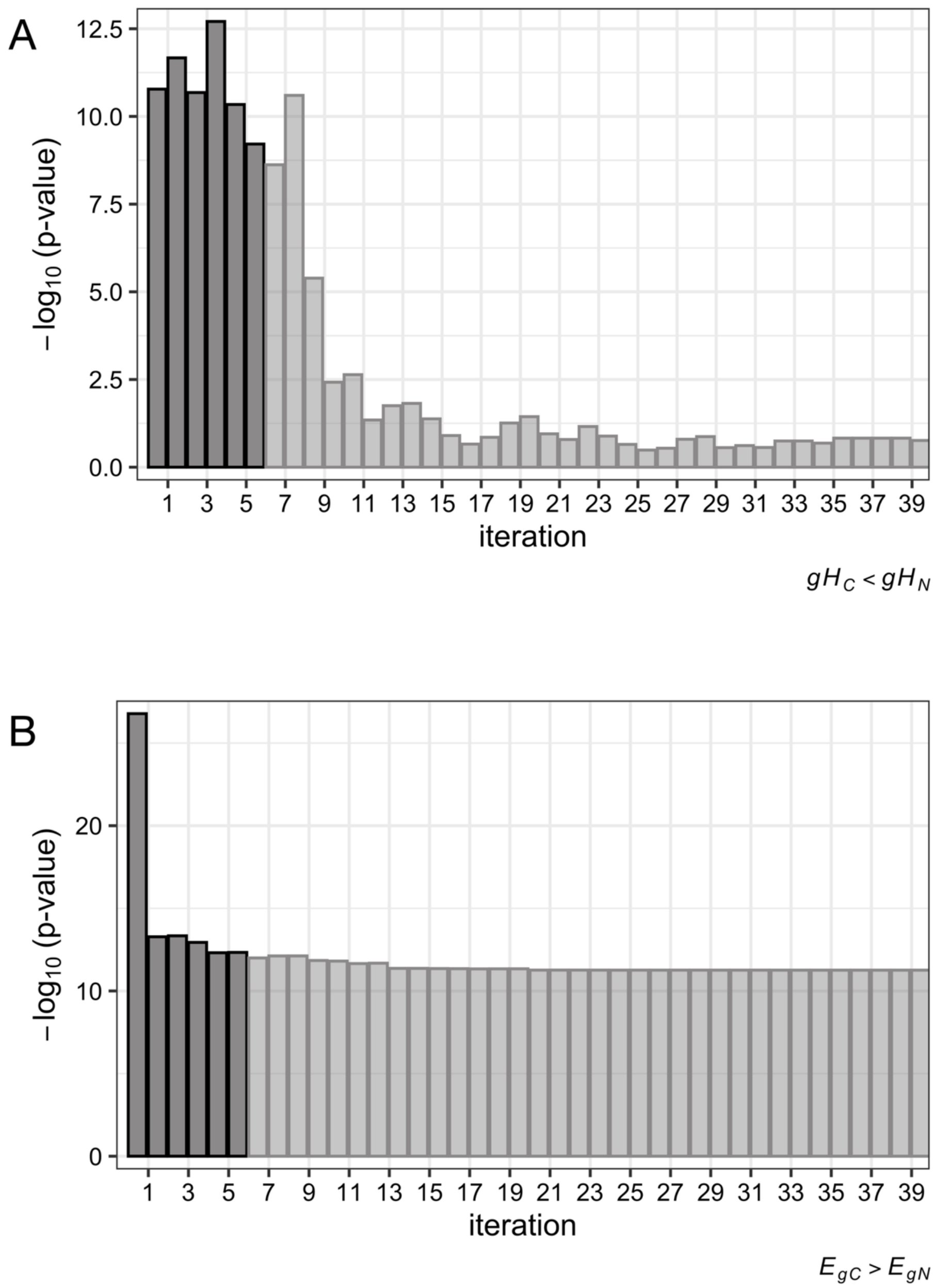

Supplementary Figure 13. Trend of gene-level entropy and energy with iterative TSS activity equalization per gene ranked with respect to the highest d: (A) Induced gH maximization by merging of TSS scores from the most distal towards the most proximal TSS shows that gH_C remains lower than gH_N when merging the terminal 10 TSSs (six iterations highlighted for comparison with the proximal to distal merging, data in Fig 2E) with high significance. **(B)** The same iterations as described for A above were used to calculate E_{gC} and E_{gN}. E_{gC} remained higher than E_{gN} over most iterations even if the maximal E_g excess in *C* is linked only to the difference in the deployment of the two most distal TSSs. Y–axes show $-\log_{10}(p)$ and X–axes show TSS score–merging iterations (p–values reported from Wilcoxon signed–rank tests paired by 11928 genes in every iteration).

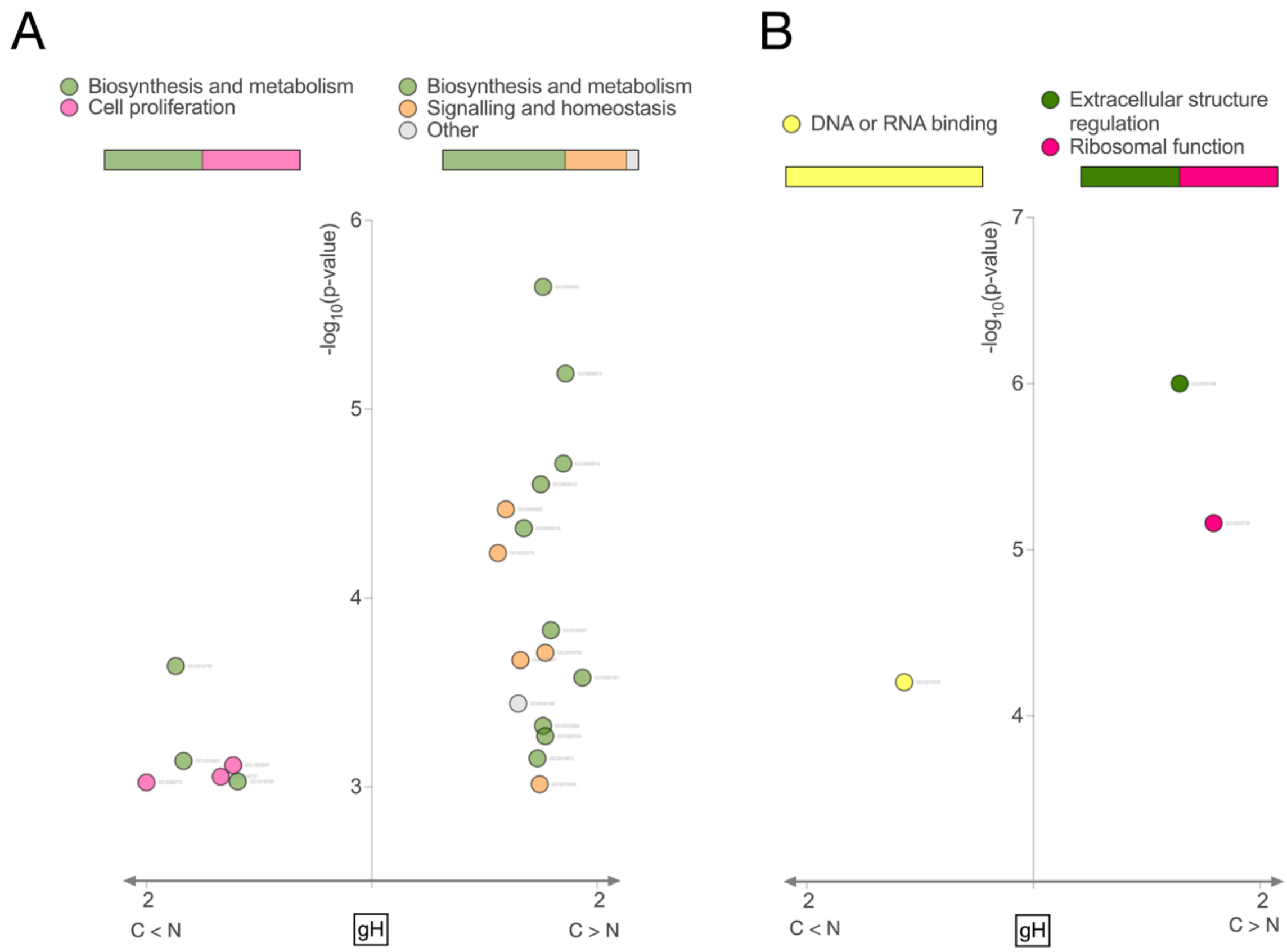

Supplementary Figure 14. Gene–level stochasticity of TSS deployment (*gH*) is retained in cancers for genes supporting biosynthesis and cell proliferation: The genes with low *gH* in *C* are significantly enriched with biological processes of biosynthesis and cell proliferation **(A)** and molecular function of DNA/RNA–binding **(B)**. Y–axes show p–value of Fisher exact text for significant enrichment and X–axes show the fold change of *gH* in *C,* over *N*; more details in table S15.

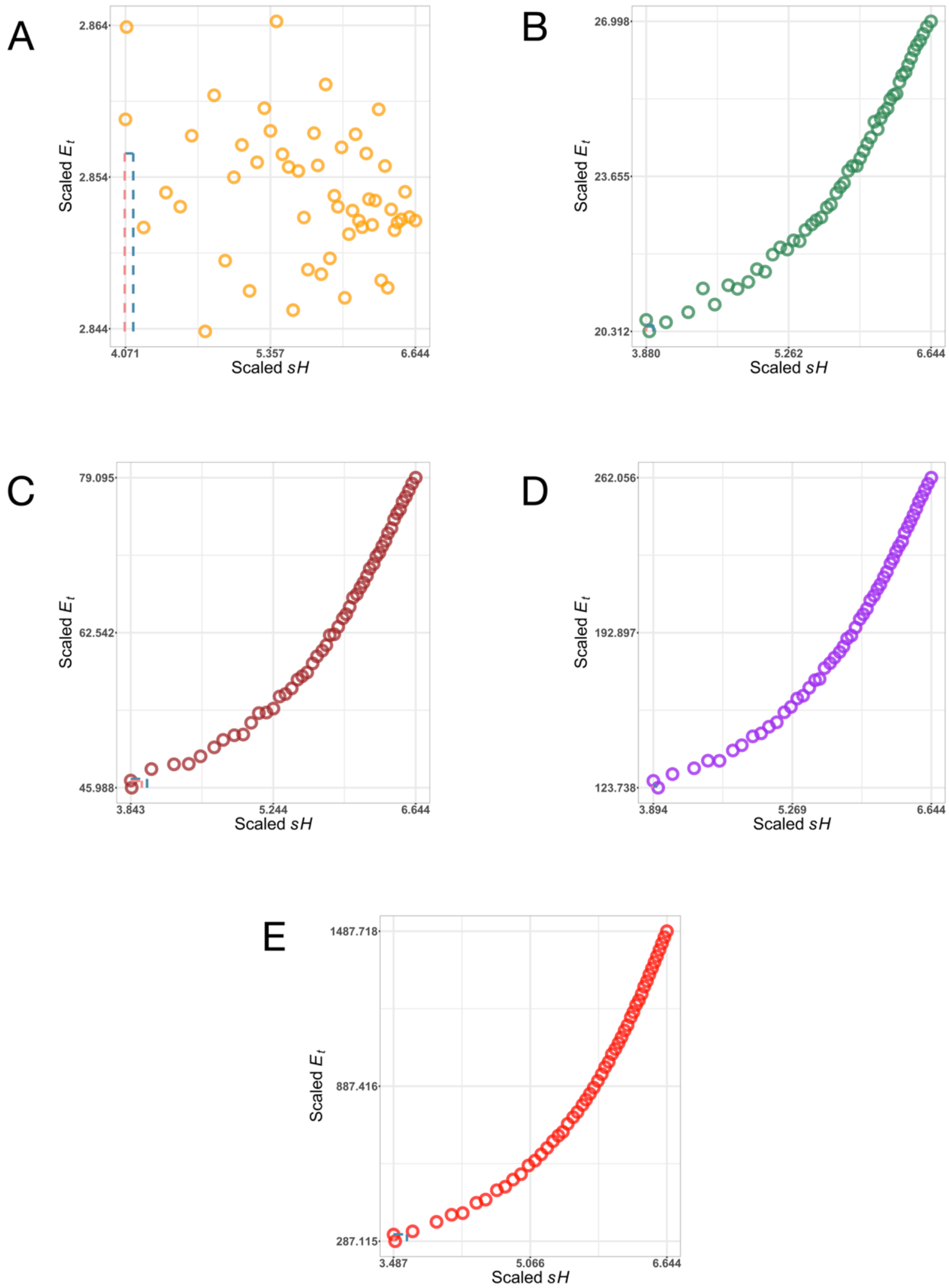

Supplementary Figure 15. Controlled sH increment of artificially generated promoteromes (see Methods, 2.13) shows that a change in sH affects E_t in a d–dependent manner: (A) TSSs with d<1kb do not show an increase in E_t as sH is forced to increase. **(B–E)** TSSs with d 1– 5kb **(B)**, 5–10 kb **(C)**, 10–50 kb **(D)** and >50 kb **(E)** all show an $E_t$increase as sH is forced to increase. The pink and blue dotted lines show E_t interpolations using actual sH from C (pink) or N (blue). Notably, only for TSSs with d<1kb the sH and E_t remain in the range showing that the sH for the d<1kb TSSs is high enough to resist further forced increment.

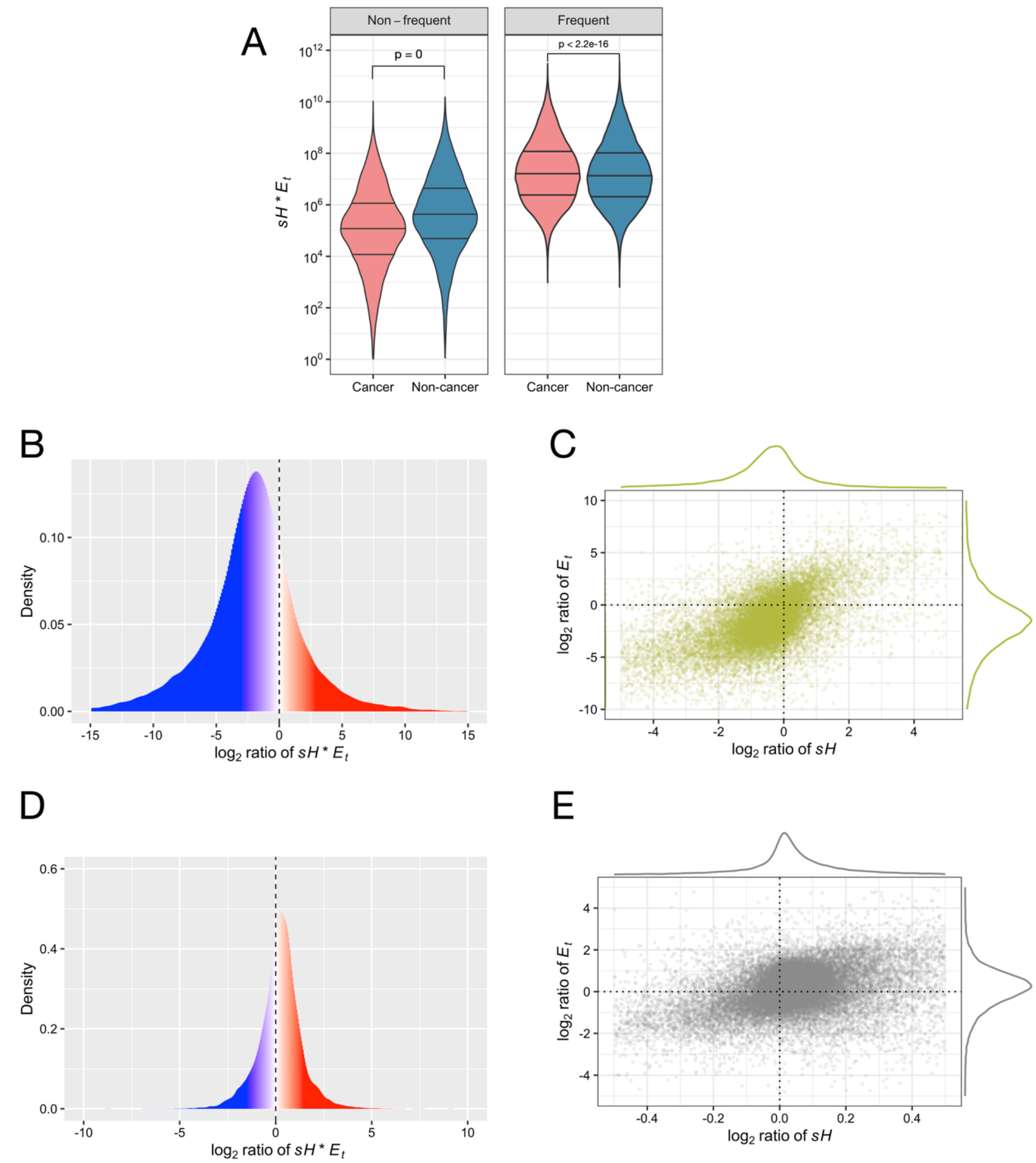

Supplementary Figure 16. sH and E_t cooperate differently in cancers and non–cancers: (A) *Non–Frequently Deployed* TSSs have $sH * E_t$ significantly lower in cancers whereas the same parameter is significantly higher in cancers for *Frequently Deployed* TSSs (Wilcoxon signed–rank test paired by 21816 and 33232 TSSs respectively, p–values < 2.2e-16). **(B and C)** (*Non–Frequently Deployed* TSSs): A density distribution of C/N ratios of $sH * E_t$ from shows a decrease in C (X–axis values <0, blue AUC). A scatter plot of C/N ratios of sH (X–axis) and E_t (Y–axis) shows that the majority of low sH and E_t (bottom left quadrant) are observed in C. **(D and E)** (*Frequently Deployed* TSSs): The distribution of C/N of $sH * E_t$ ratios (**D**), and sH versus E_t scatter plot (**E**) for the *Frequently Deployed* TSSs shows that the *Frequently Deployed* TSSs have a pattern of cooperation between sH and E_t which is opposite to that observed for *Non–Frequently Deployed* TSSs.

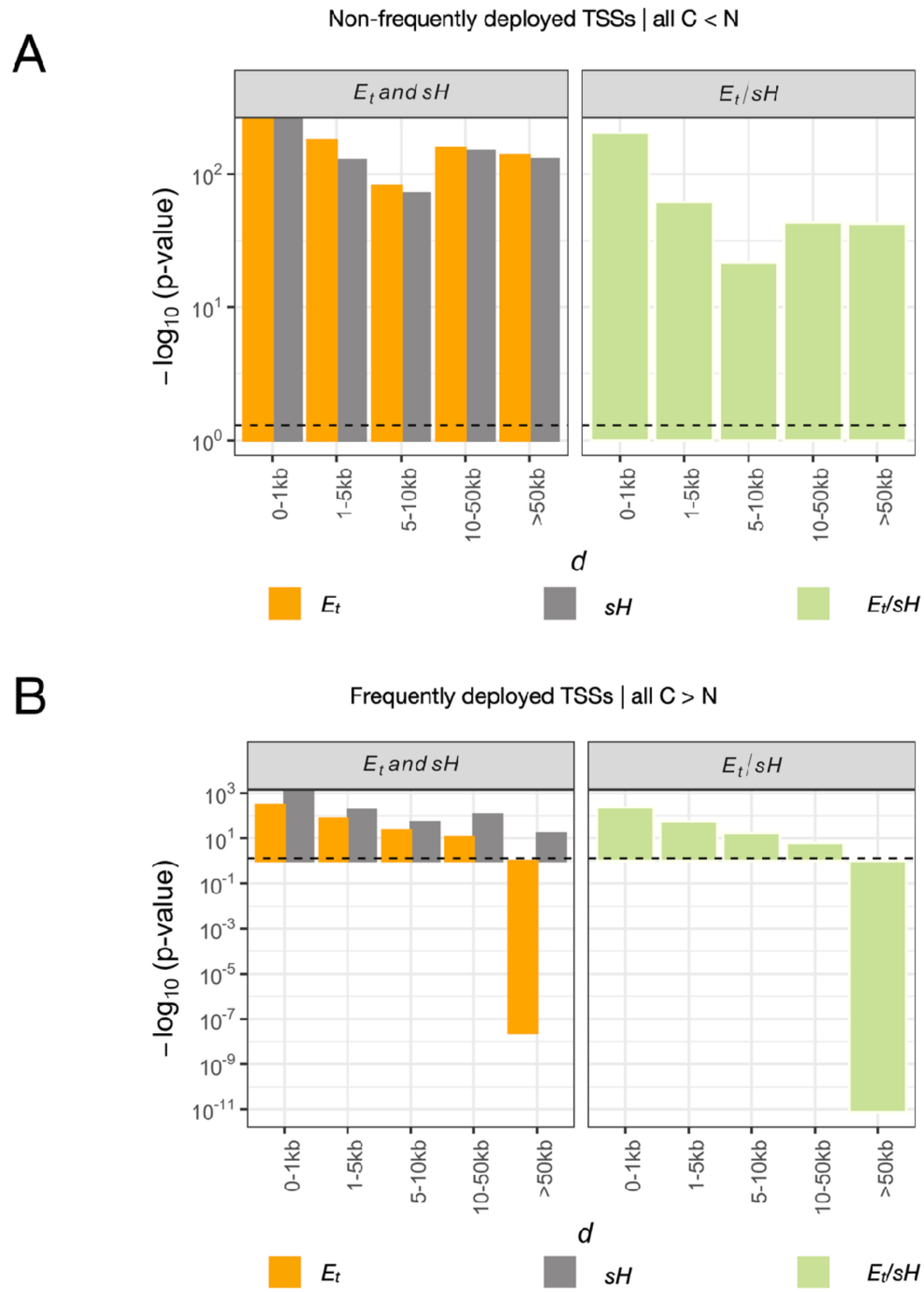

Supplementary Figure 17. *Frequently* or *Non–Frequently Deployed* TSSs exhibit different d–dependent patterns of E_t as a function of sH (calculated as E_t/sH) in *C* and *N:* (A) *Non–Frequently Deployed* TSSs show a decline in the significance of E_t/sH difference (*C*<*N*) with increasing d till 10kb but increase at TSSs with d >10kb. However, at all d values the difference remains highly significant (Wilcoxon signed–rank test paired by TSSs in the d-bins, p–value < 0.05). **(B)** *Frequently Deployed* TSSs (CAGE scores available from ≥ 100 samples) show a declining significance of difference (*C* > *N*), albeit significant, still ≤50kb. Extremely distal TSSs (>50kb) show no significant increase in *C* as compared to *N* (Wilcoxon signed–rank test paired by TSSs in the d-bins, p–value < 0.05 except for the last bin). Parameters are color-coded: E_t (orange), sH (grey). E_t/sH (green). X–axes show the discrete TSS d–bins and Y–axes show $-\log_{10}(p)$ of the test of significance. p–value of 0.05 indicated by a horizontal dashed line.

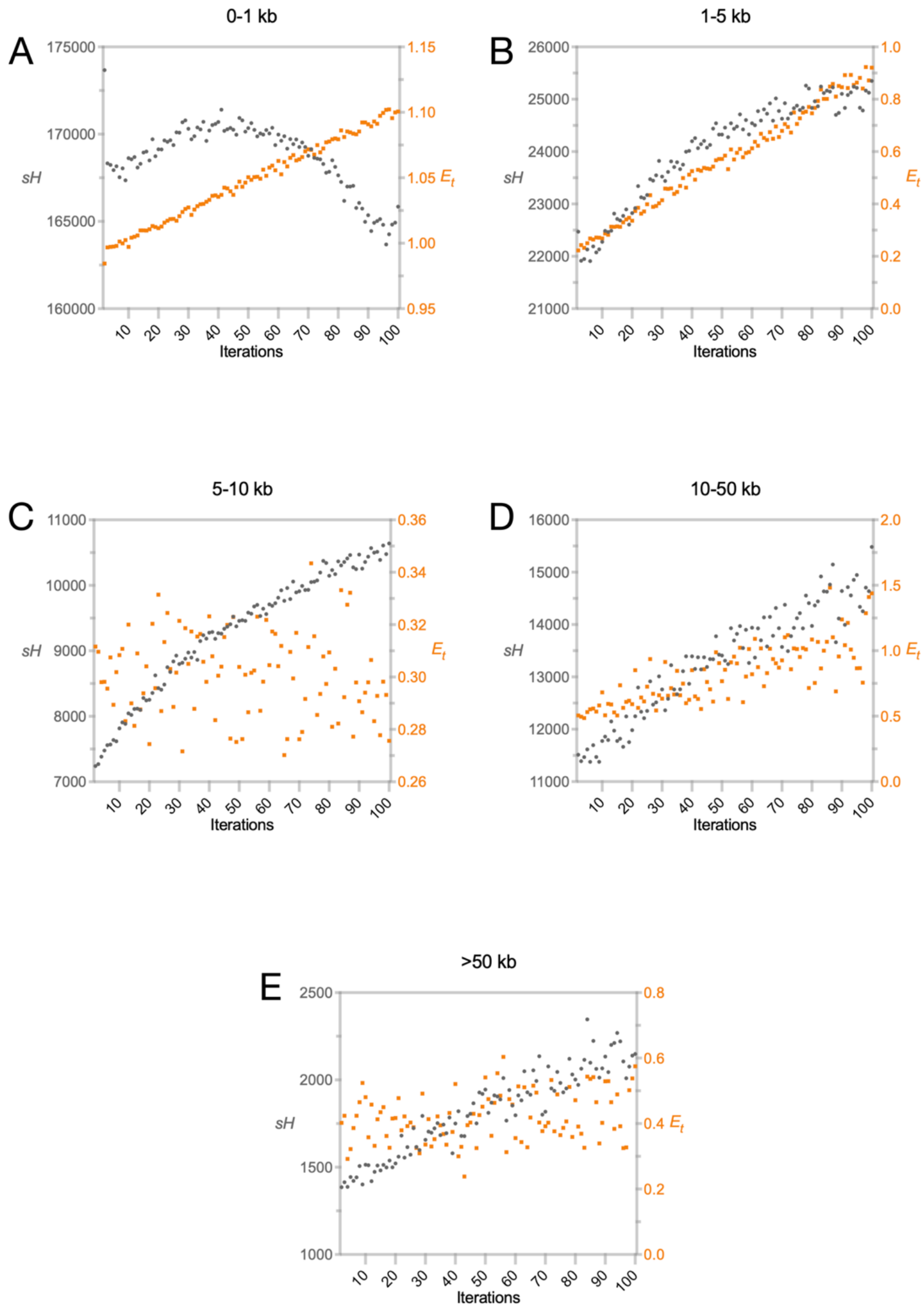

Supplementary Figure 18. *sH* differences between *C* and *N* are revealed by iterative and incremental substitution of CAGE scores of *C* with those from *N*: The different scatter plots represent data split by d–bins; 0–1 kb **(A)**, 1–5 kb **(B)**, 5–10 kb **(C)**, 10–50 kb **(D)** and >50 kb **(E)**. The entire substitution spanning 50 independently performed iterations on randomized inputs is plotted from 0% *N* and 100% *C* (left ends of the X–axes) to 100% *N* and 0% *C* (right ends of the X–axes). Left Y–axes show sH and right Y–axes show corresponding E_t. A complete disconnect between E_t (increasing with iterations) and sH (decreasing upon restoration of *C* to *N*) is visible only for the TSSs with d<1kb (A) with other bins showing varying degrees of direct correlation between $E_t$and sH (B–E). The findings (panel A compared with the rest) demonstrate that higher sample–space entropy in *C* conserves E_t only for the most proximal TSSs.

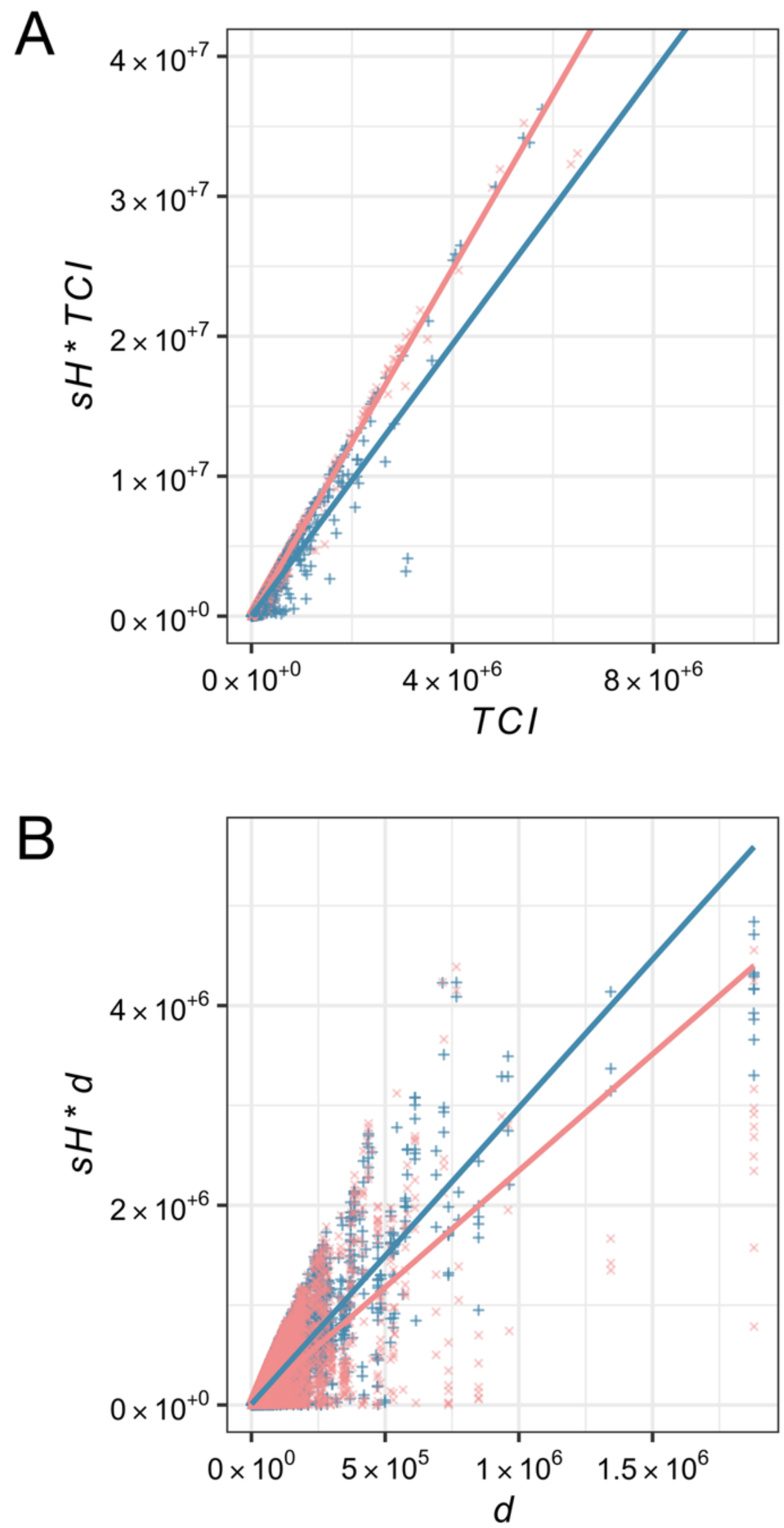

Supplementary Figure 19. E_t increase in C is due to transcriptional activity–excess which overrides the d conservation in cancers. sH–linked TCI is increased in C (**A**) whereas sH–linked d is decreased in C (**B**). The units of $sH * TCI$ and $sH * d$ and TCI are arbitrary.

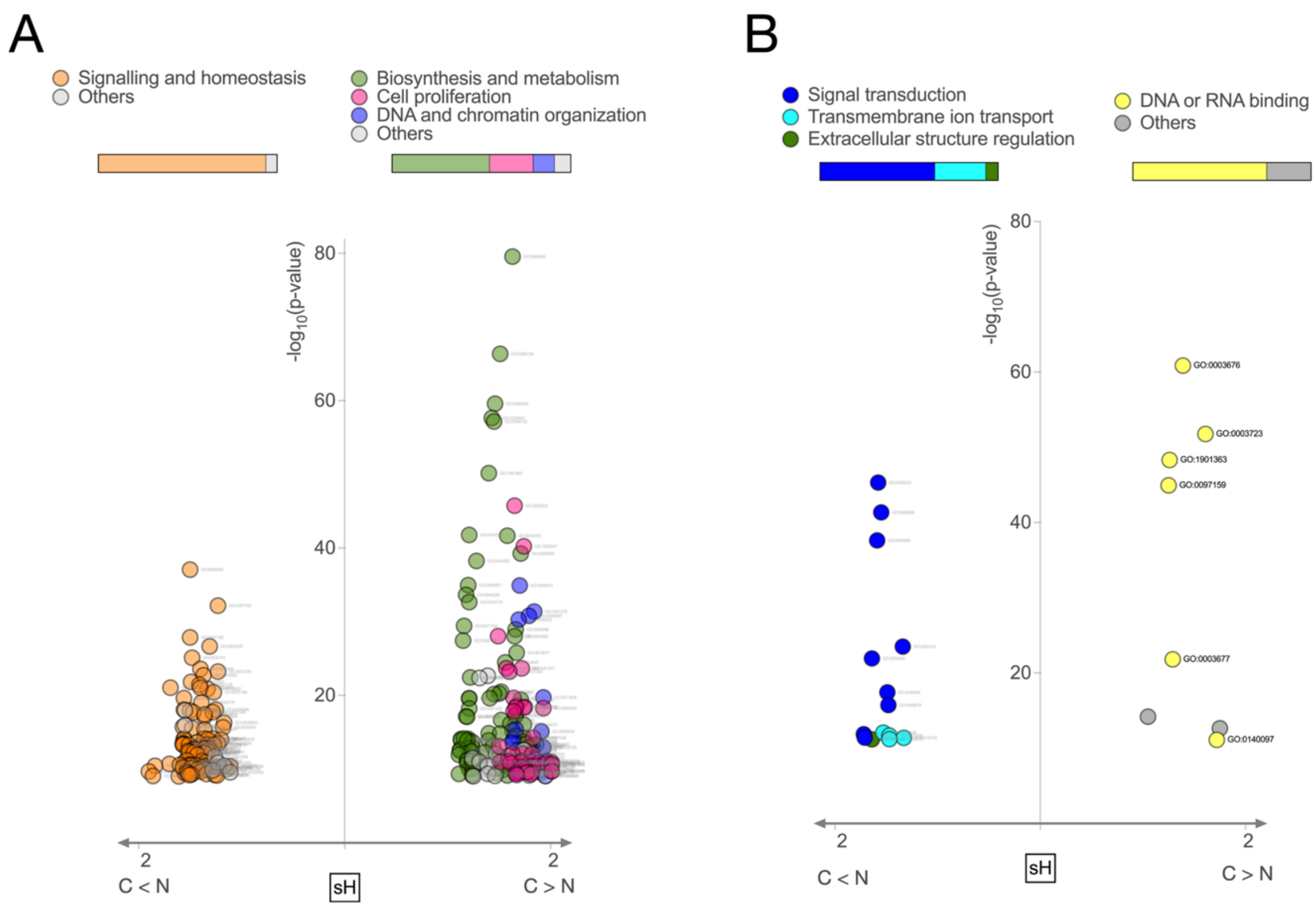

Supplementary Figure 20. Frequent and stochastic deployment of genes involved in biosynthesis and cell proliferation across cancers as revealed by *sH*: High sH in cancers is significantly associated with genes involved in biological processes biosynthesis and cell proliferation **(A)** and molecular functions DNA/RNA–binding **(B)**, recapitulating the gene functional category enrichments observed for vd and gH (details in table S16).

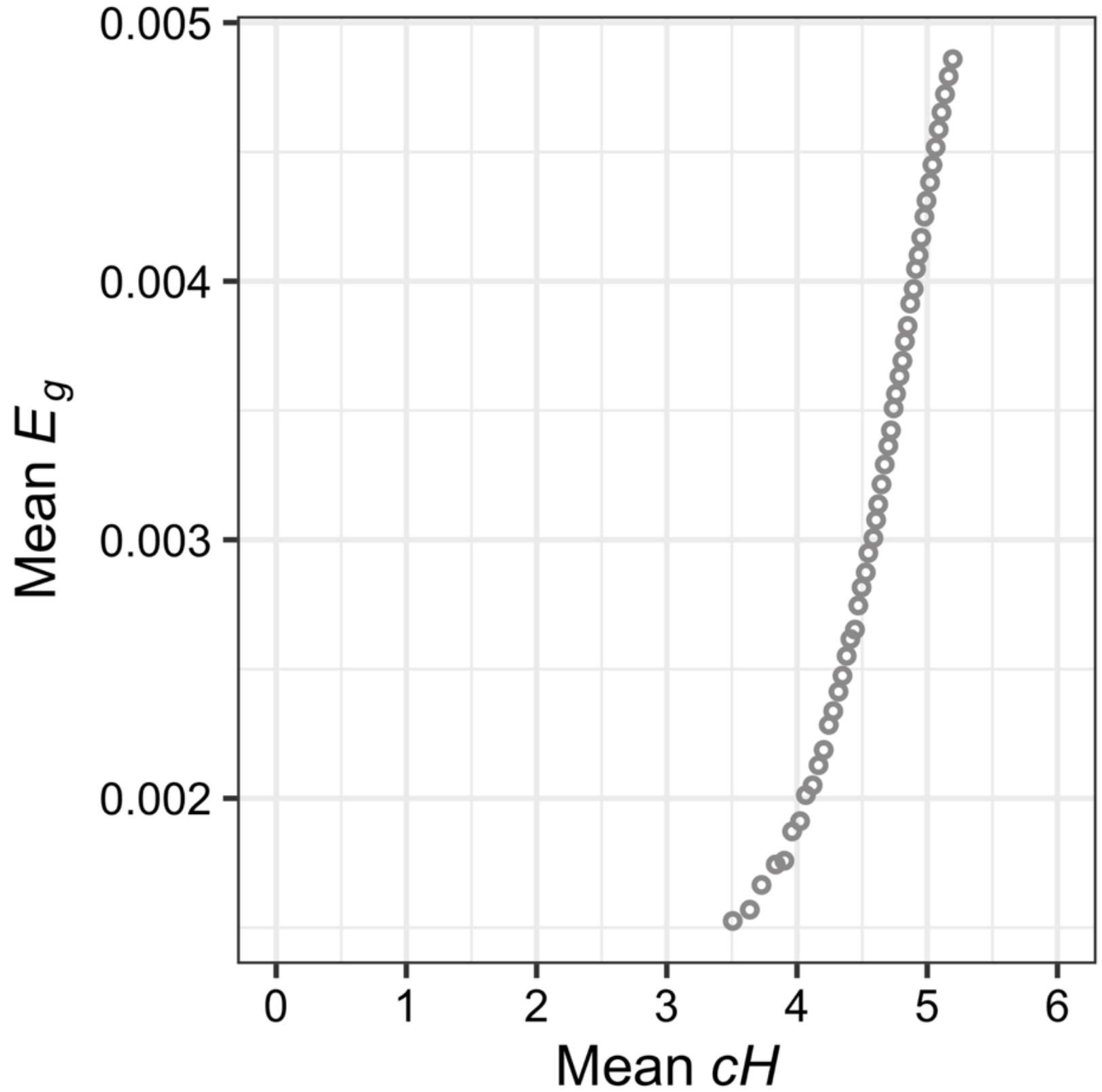

Supplementary Figure 21. Artificial promoterome reconstructed using actual CAGE data with equal contributions from *C* and *N* randomly shows that E_g increases rapidly with externally forced increase in cH: The actual cH and E_g observed in CAGE samples range similar in cH but much lower in E_g. The gene identity–blind increase in TSS scores increases E_g tremendously. cH and associated low E_g observed in actual samples is thus a relationship governed by gene–level bundling of TSSs implying a gene function feedback governing cH and related E_g.

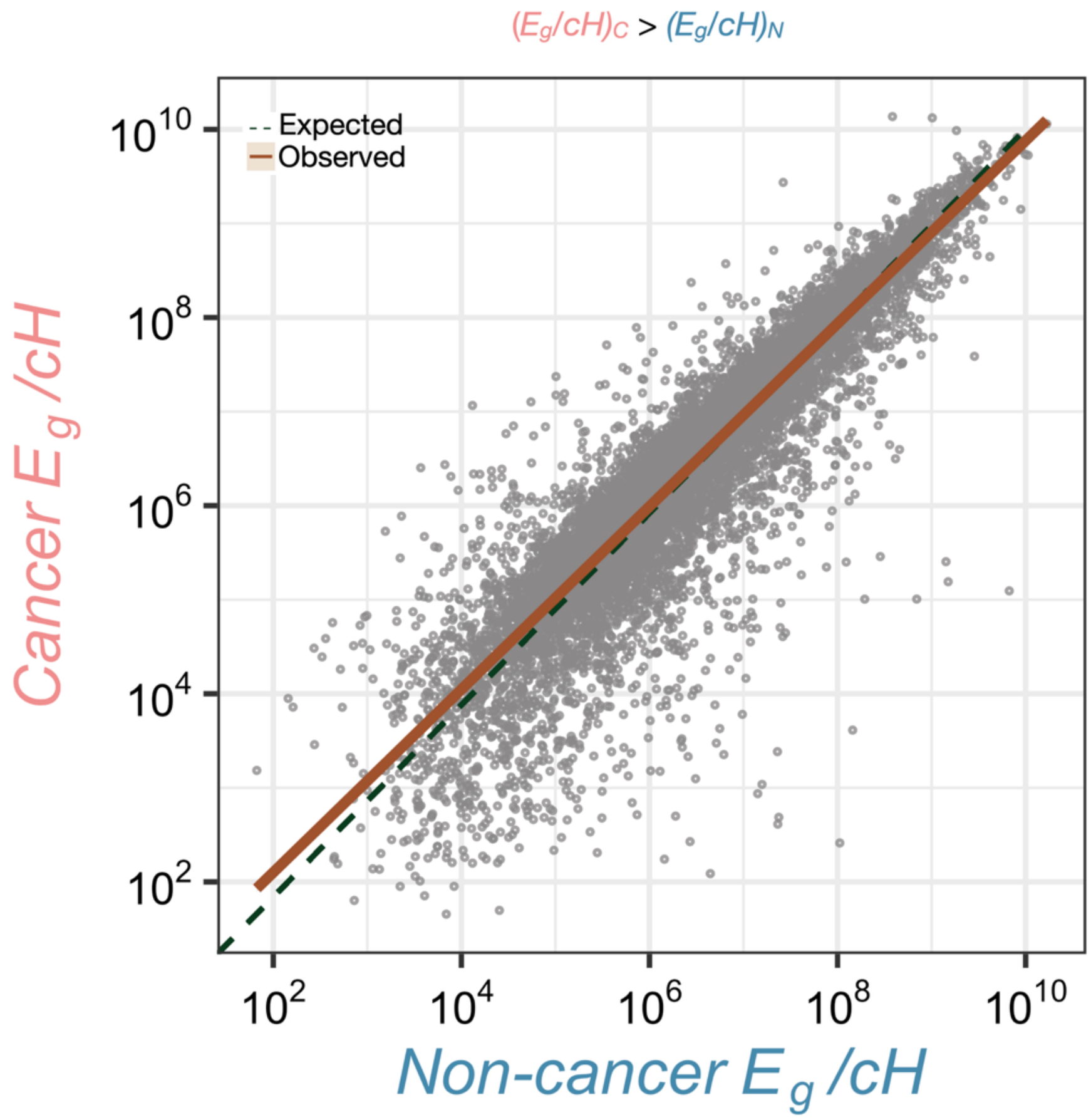

Supplementary Figure 22. E_g as a function of cH, calculated as E_g/cH comparison between *C* and *N* shows similarity between *C* and *N* with a *C*–biased distribution: For every unit increase in cH, E_g increases in *C* more than that in *N*. The correlation scatter plot shows that this trend holds true for most genes.

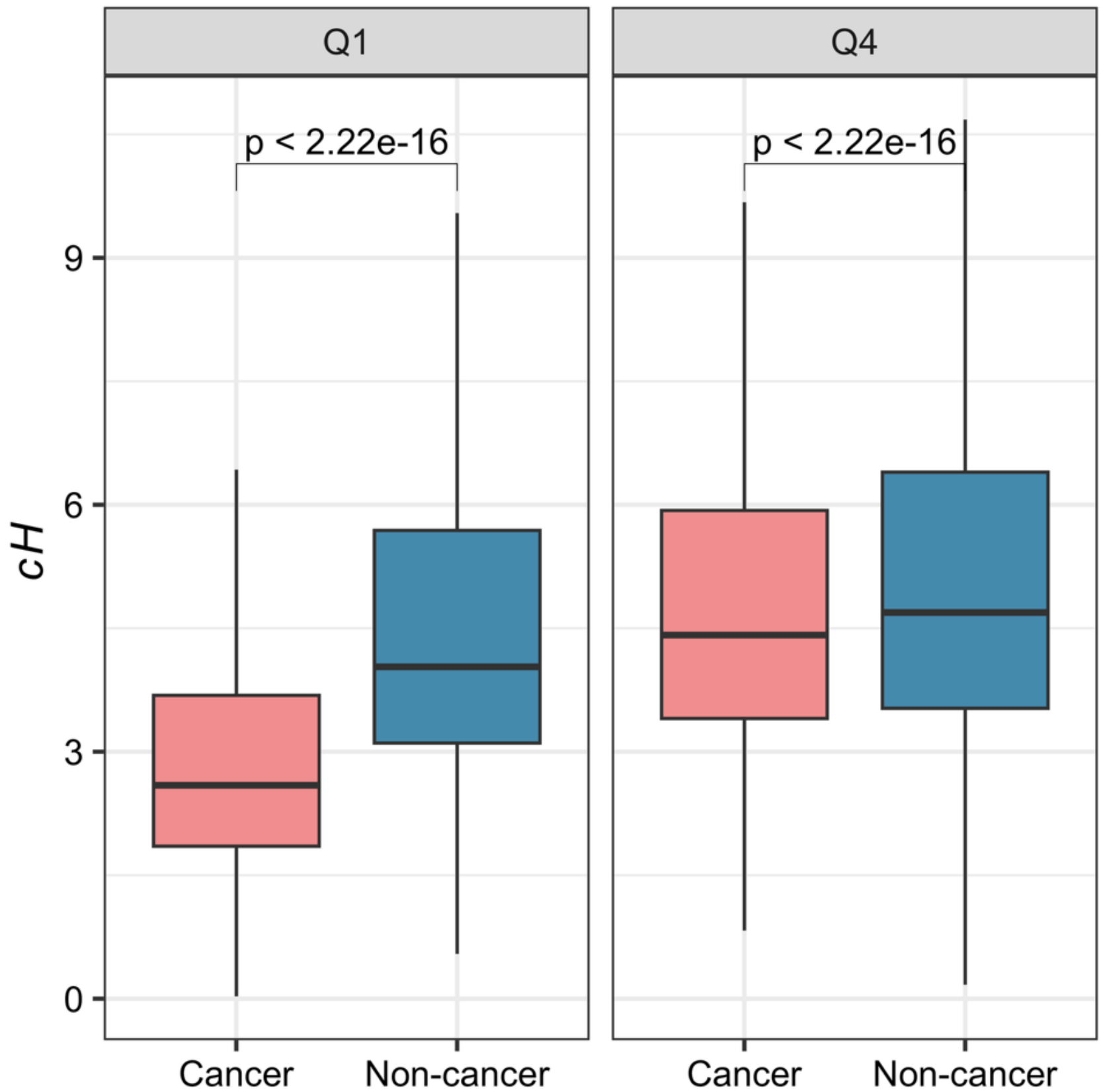

Supplementary Figure 23. *cH* is overall lower in *C* than *N*: The Q1 as well as Q4 (terminal quartiles) of cH_C/cH_N have cH significantly lower in cancers but the magnitude of difference is stronger in Q1 genes.

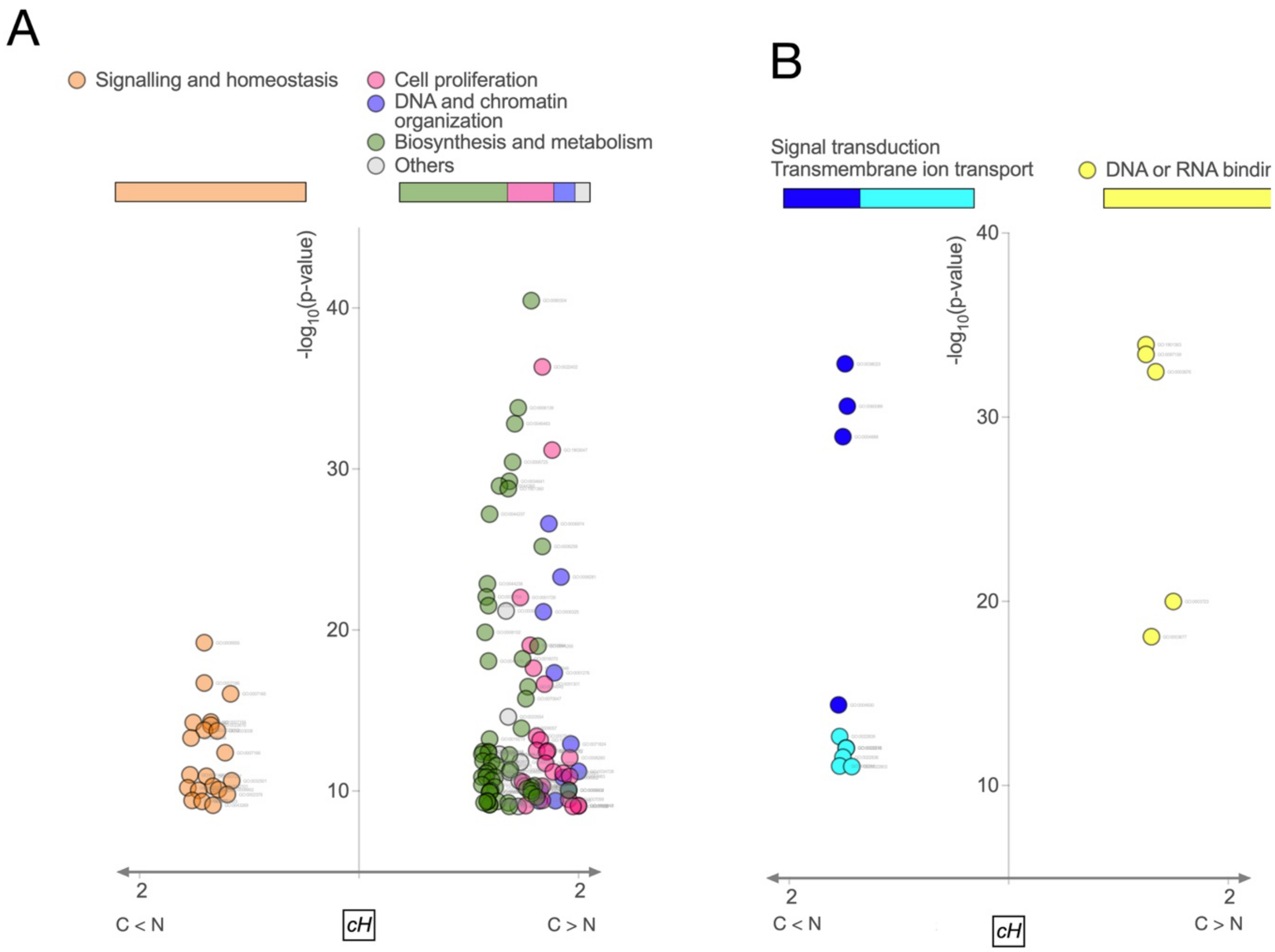

Supplementary Figure 24. *cH* difference between *C* and *N* is linked to gene functions: cH, although overall lower in C compared to N, shows a strong enrichment in C for genes involved in the same cancer hallmark functions as observed earlier for vd, gH and sH, including biological processes biosynthesis, chromatin organization and cell proliferation **(A)**, and molecular functions DNA or RNA–binding are noteworthy **(B)**. Y–axes show p–value of Fisher exact test for significant enrichment using GOrilla Gene Ontology term enrichment and X–axes show the fold change of cH in *C,* over *N*. Specific Gene Ontology terms (marked in light grey fonts for every data point) are merged into color–coded assigned terms presented as legends on the top of the scatter plots.

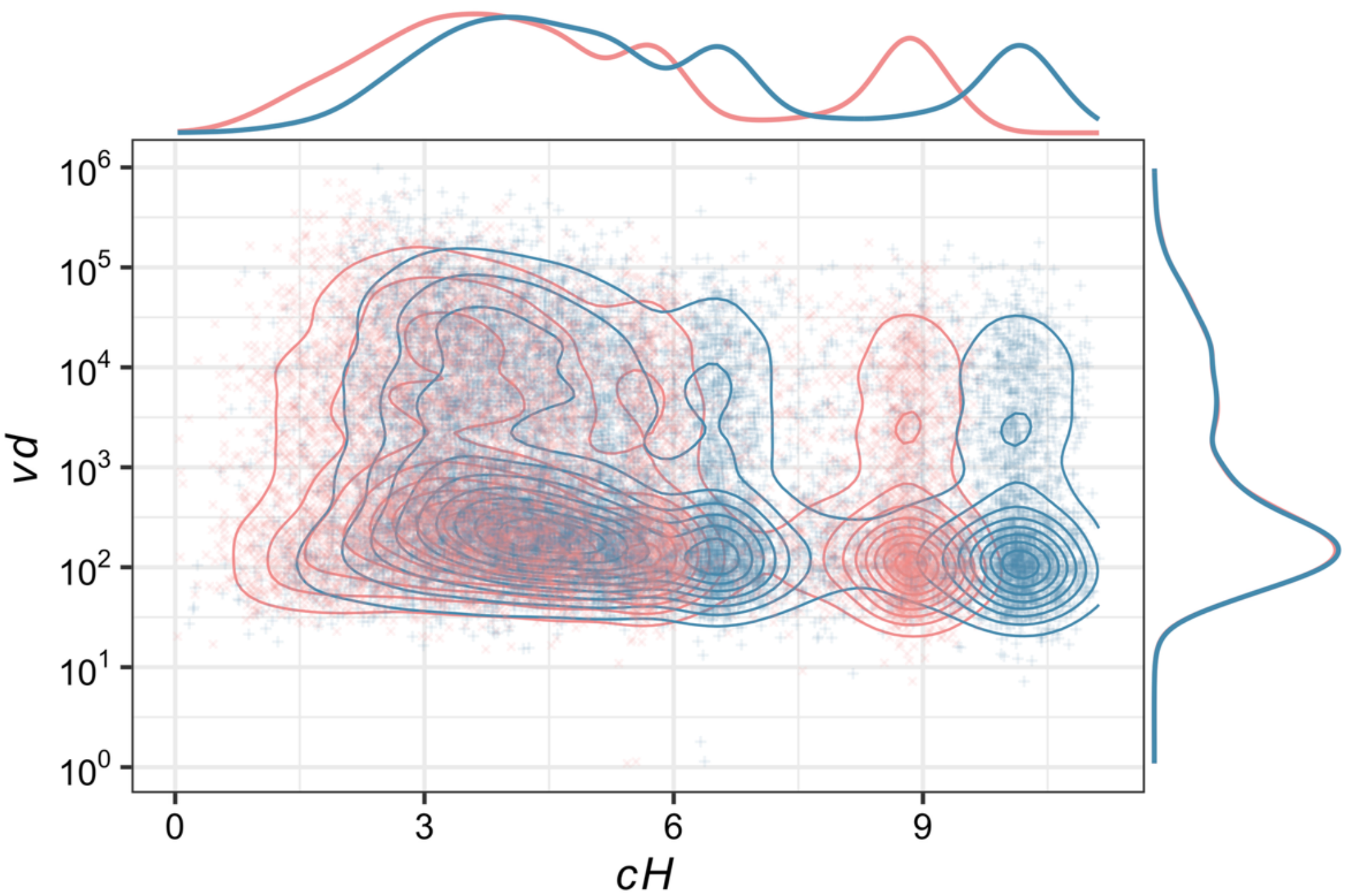

Supplementary Figure 25. Overall distribution of cH (X–axis) and vd (Y–axis) for 11928 genes in C (pink) and N (blue): The vd differences between C and N arise at the lowest and the highest extremes of cH. vd in C is lower than that in N at cH values lower than 5. It shows that the enhanced preference for extremely low d TSSs is less stochastic, and by corollary more deterministic, in nature. The scatter does not present the data as paired between C and N by gene identities. The pairing is only by the plotted parameters cH and vd.

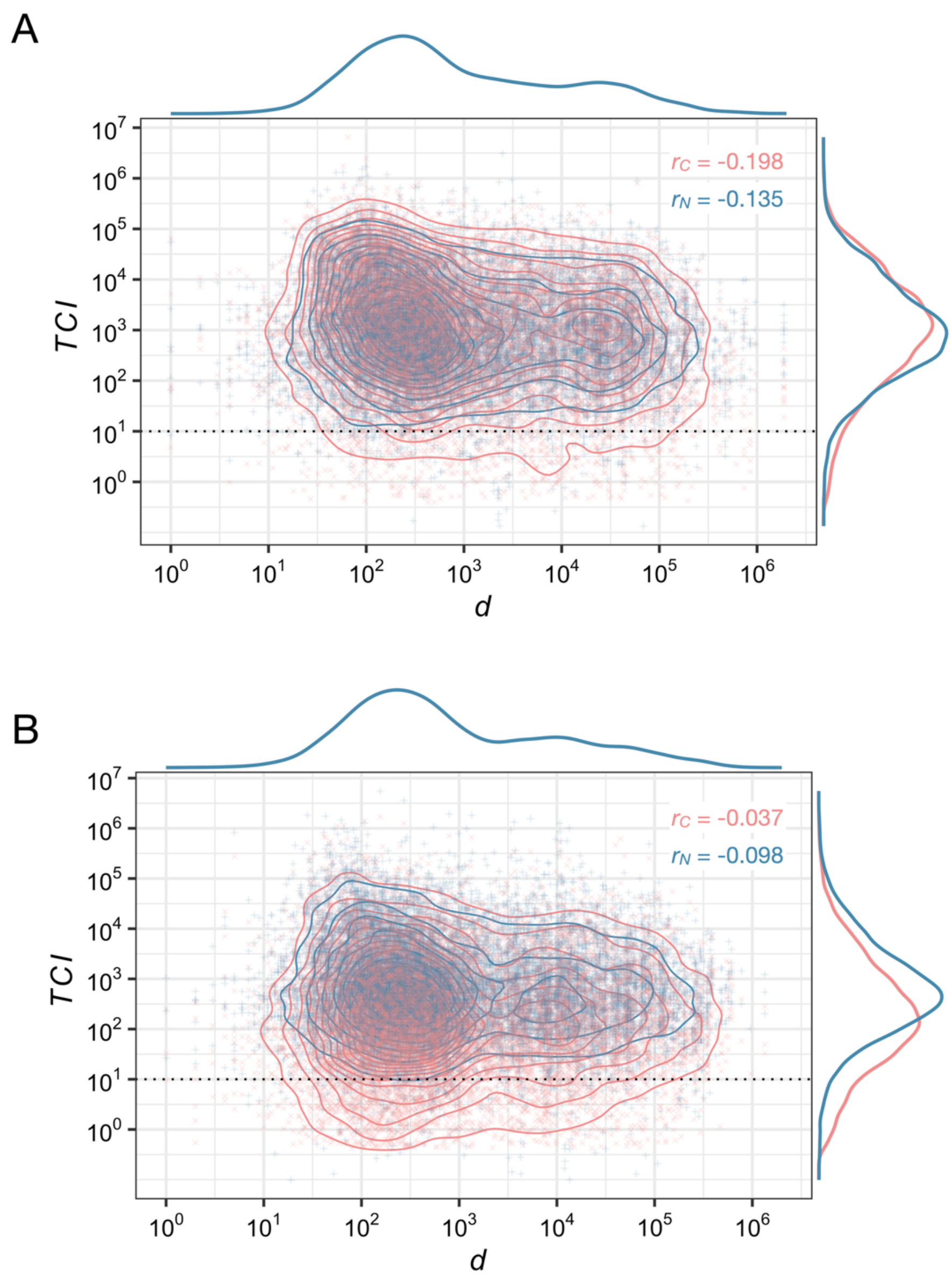

Supplementary Figure 26. Leaky deployment of TSSs across the entire range of d account for E_g differences between C and N. Terminal quartiles of genes sorted by vd/cH C/N ratios contrasting differences in Eg between C and N. Q1 genes **(A)** have higher TCI in C. For Q4 genes **(B)** however, across the entire spectrum of d TSSs exhibit low TCI in C reminiscent of leaky TSS deployments. The dashed line represents an arbitrary threshold at which the TCI differences between C and N are evident.

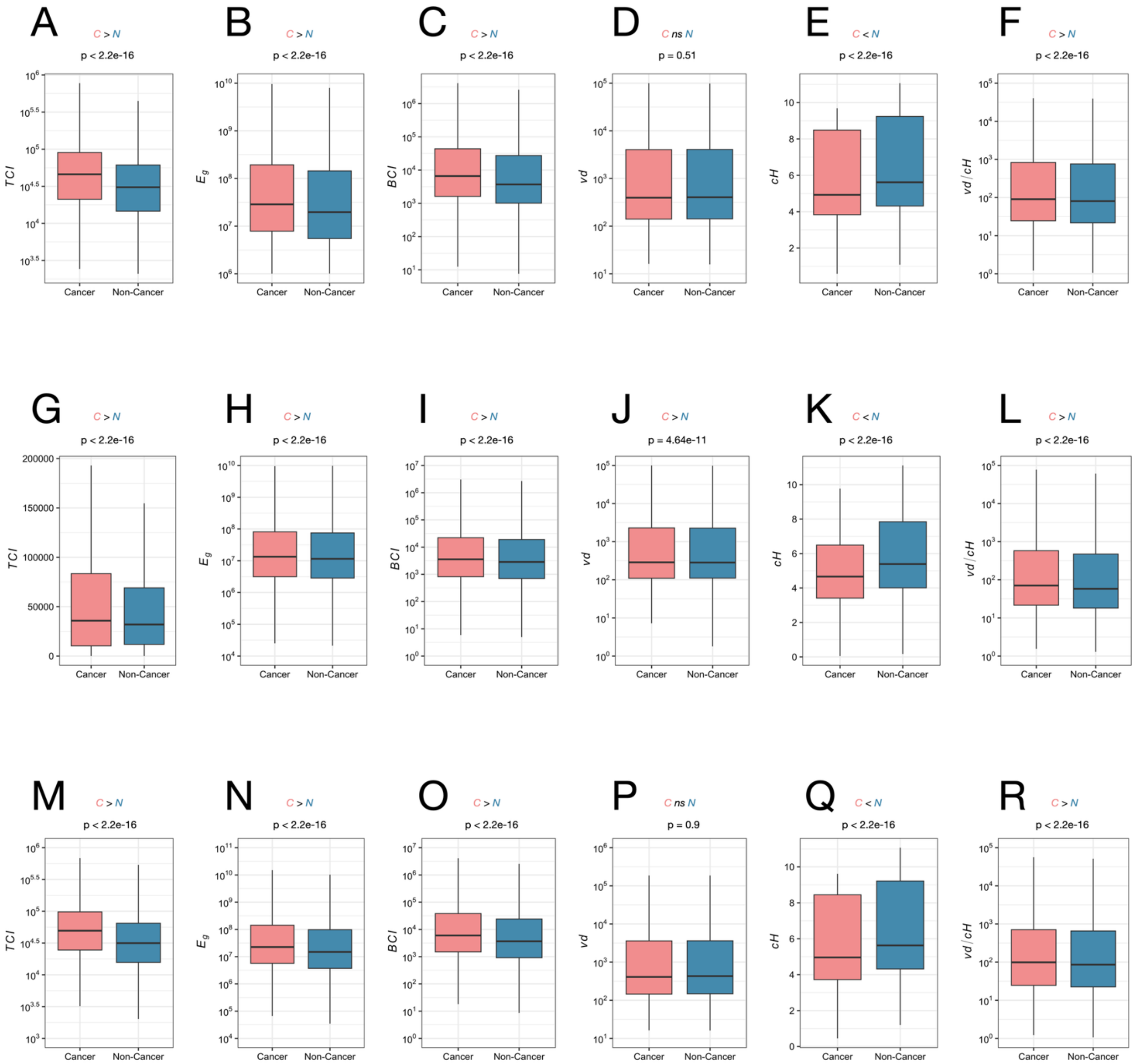

Supplementary Figure 27. Differences between *C* and *N* for the parameters (identified through vd_C/vd_N) TCI, E_g, BCI, vd, cH and vd/cH are properties related to gene function and not specific gene identities or locations: Significant *C* versus *N* differences exist for all the genes contained in the GO terms differentially enriched in cancers. DNA and chromatin organization **(A–F)**, Biosynthesis and metabolism **(G–L)**, Cell proliferation **(M–R)**, TCI **(A, G and M)**, E_g **(B, H and N)**, BCI **(C, I and O)**, vd **(D, J and P)**, cH **(E, K and Q)**, vd/cH **(F, L and R)**. Actual GO terms for which the genes were fetched for the analyses from GeneOntology database are mentioned in table S14. Samples are indicated along the X–axes and the Y–axes depict the parameters described above and indicated by the axes. Values on the Y–axes are median ± IQR and 95% CI. Differences between *C* and *N* are indicated at the top of each plot with an accompanying p–value (Wilcoxon signed–rank test paired by 947 genes (**A–F**), 5031 genes (**G–L**) and, 781 genes (**M–R**), p–values ≤ 4.64e-1.1).

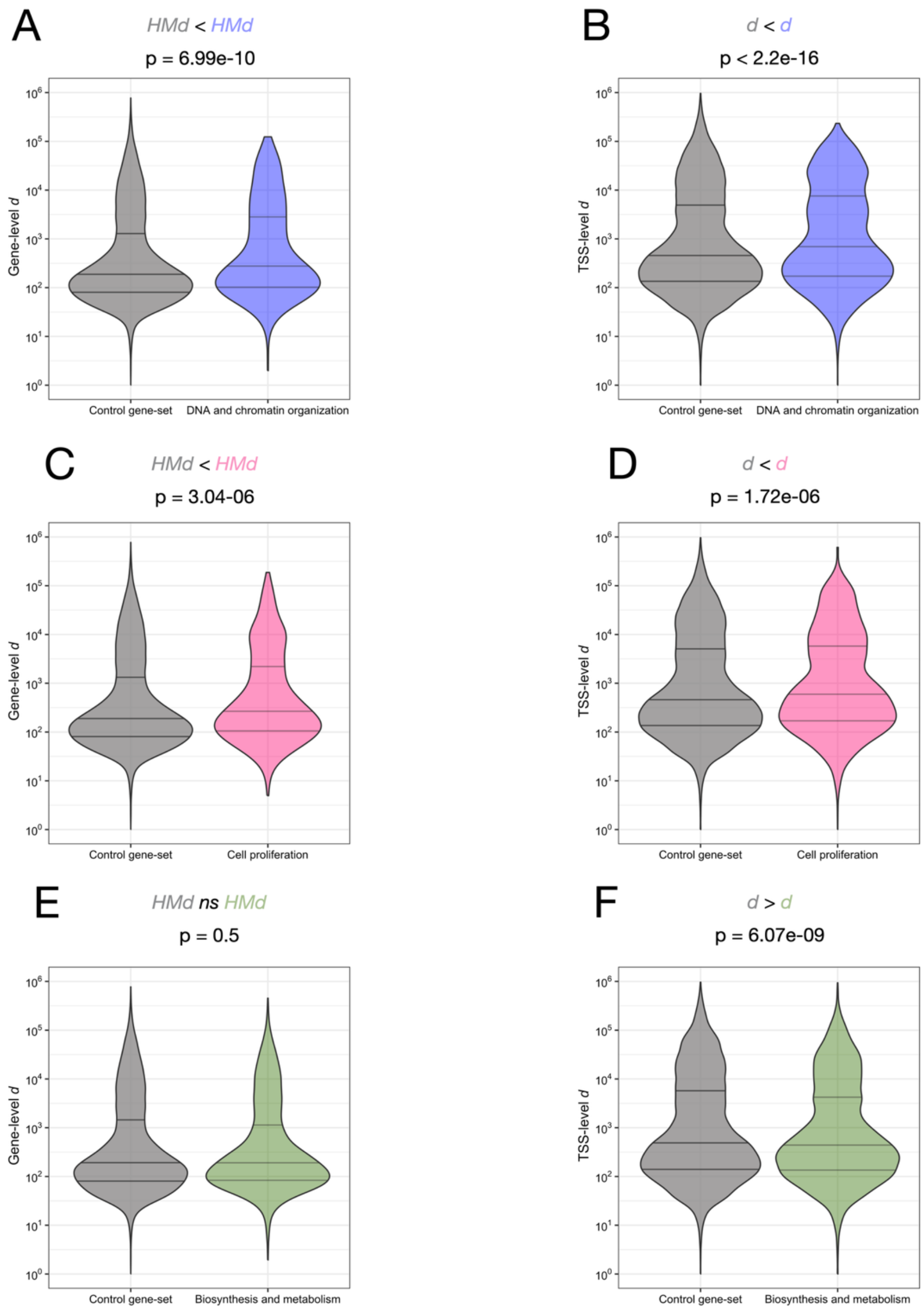

Supplementary Figure 28. Genes belonging to the differentially enriched functional categories between C and N (identified through vd_C/vd_N) have significantly different d profiles as compared to the rest of the genes (A–F); Wilcoxon rank–sum test, p–value ≤ 1.72e-6. Genes belonging to the functional categories are indicated along the X–axes by the assigned terms and the term Control gene–set (referring to all genes except the genes belonging to the functional category plotted to the right in the same graph. Y–axes represent the indicated parameters gene–level d (HMd) or TSS–level d. Values on the Y–axes are median $\pm$ IQR and 95% CI. DNA and chromatin organization **(A and B)**, cell proliferation **(C and D)** and biosynthesis and metabolism **(E and F)**, HMd **(A, C and E)**, d **(B, D and F)**. Actual GO terms for which the genes were fetched for the analyses. From GeneOntology database are mentioned in table S14.

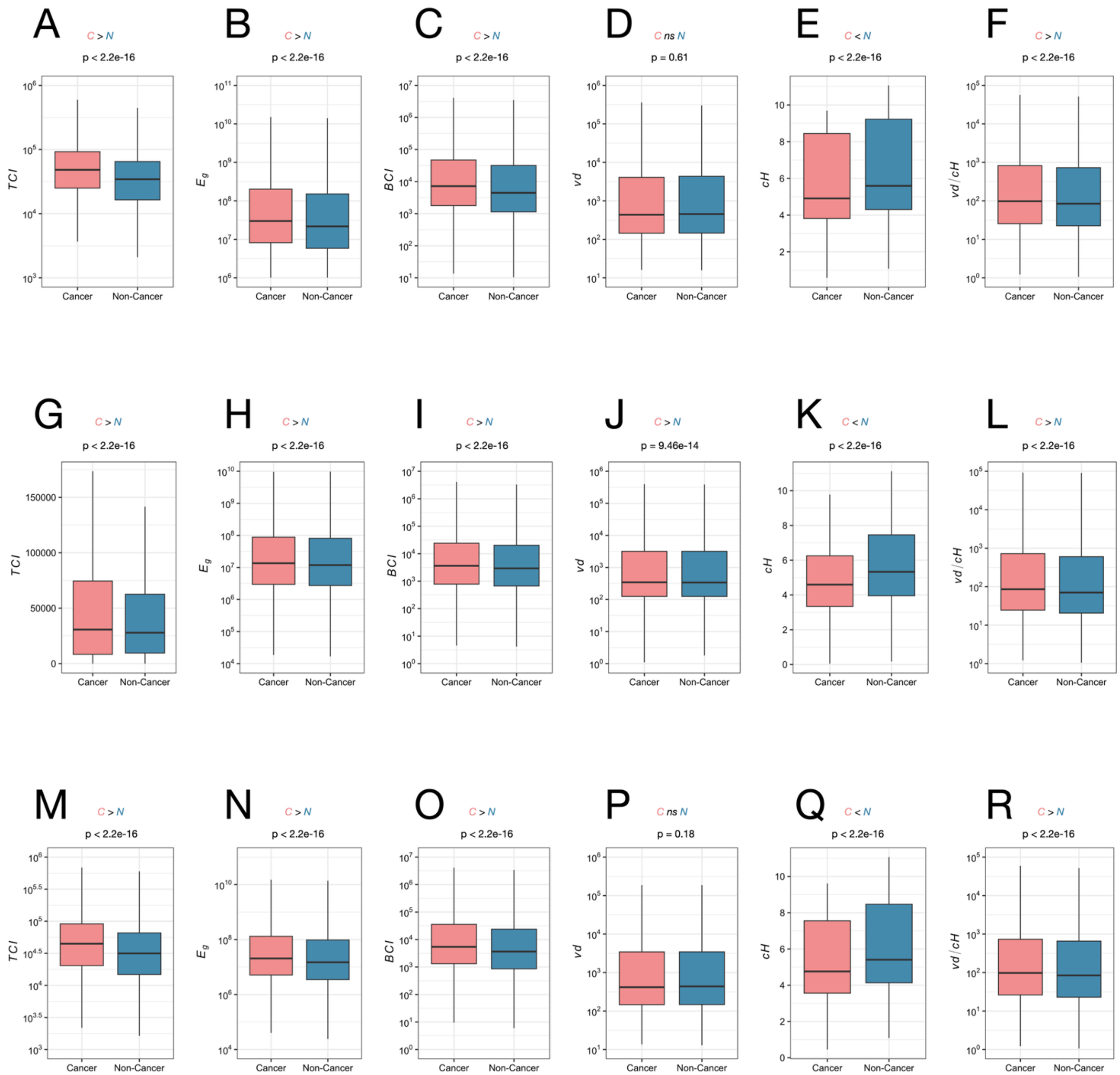

Supplementary Figure 29. Differences between *C* and *N* for the parameters (identified through C/N of vd/cH ratios) TCI, E_g, BCI, vd, cH and vd/cH are properties related to gene function and not specific gene identities or locations: Significant *C* versus *N* differences exist for all the genes contained in the GO terms differentially enriched in cancers. DNA and chromatin organization **(A–F)**, Biosynthesis and metabolism **(G–L)**, Cell proliferation **(M–R)**, TCI **(A, G and M)**, E_g **(B, H and N)**, BCI **(C, I and O)**, vd **(D, J and P)**, cH **(E, K and Q)**, vd/cH **(F, L and R)**. Actual GO terms for which the genes were fetched for the analyses from GeneOntology database are mentioned in table S17. Samples are indicated along the X–axes and the Y–axes depict the parameters described above and indicated by the axes. Values on the Y–axes are median $\pm$ IQR and 95% CI. Differences between *C* and *N* are indicated at the top of each plot with an accompanying p–value (Wilcoxon signed–rank test paired by 1158 genes (**A–F**), 6506 genes (**G–L**) and, 1237 genes (**M–R)** p–values $\leq$ 9.64e-14)**.**

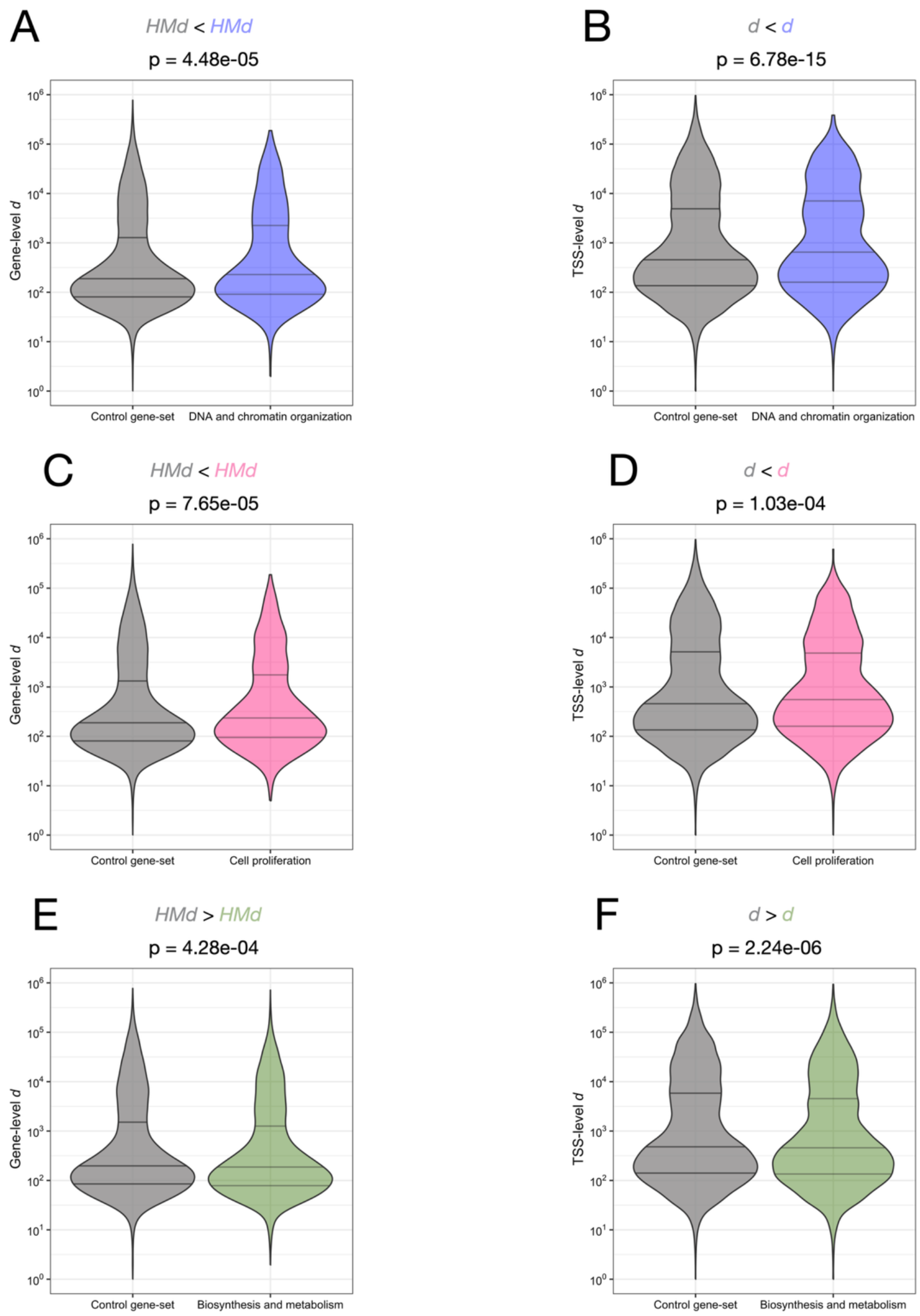

Supplementary Figure 30. Genes belonging to the differentially enriched functional categories between C and N (identified through C/N of vd/cH ratios) have significantly different d profiles as compared to the rest of the genes (A–F); Wilcoxon rank–sum test, p–value $\leq$1.72e-6. Genes belonging to the functional categories are indicated along the X–axes by the assigned terms and the term Control gene–set (referring to all genes except the genes belonging to the functional category plotted to the right in the same graph. Y–axes represent the indicated parameters gene–level d (HMd) or TSS–level d. Values on the Y–axes are median $\pm$ IQR and 95% CI. DNA and chromatin organization **(A and B)**, Cell proliferation **(C and D)** and Biosynthesis and metabolism **(E and F)**, HMd **(A, C and E)**, d **(B, D and F)**. Actual GO terms for which the genes were fetched for the analyses from GeneOntology database are mentioned in table S17.

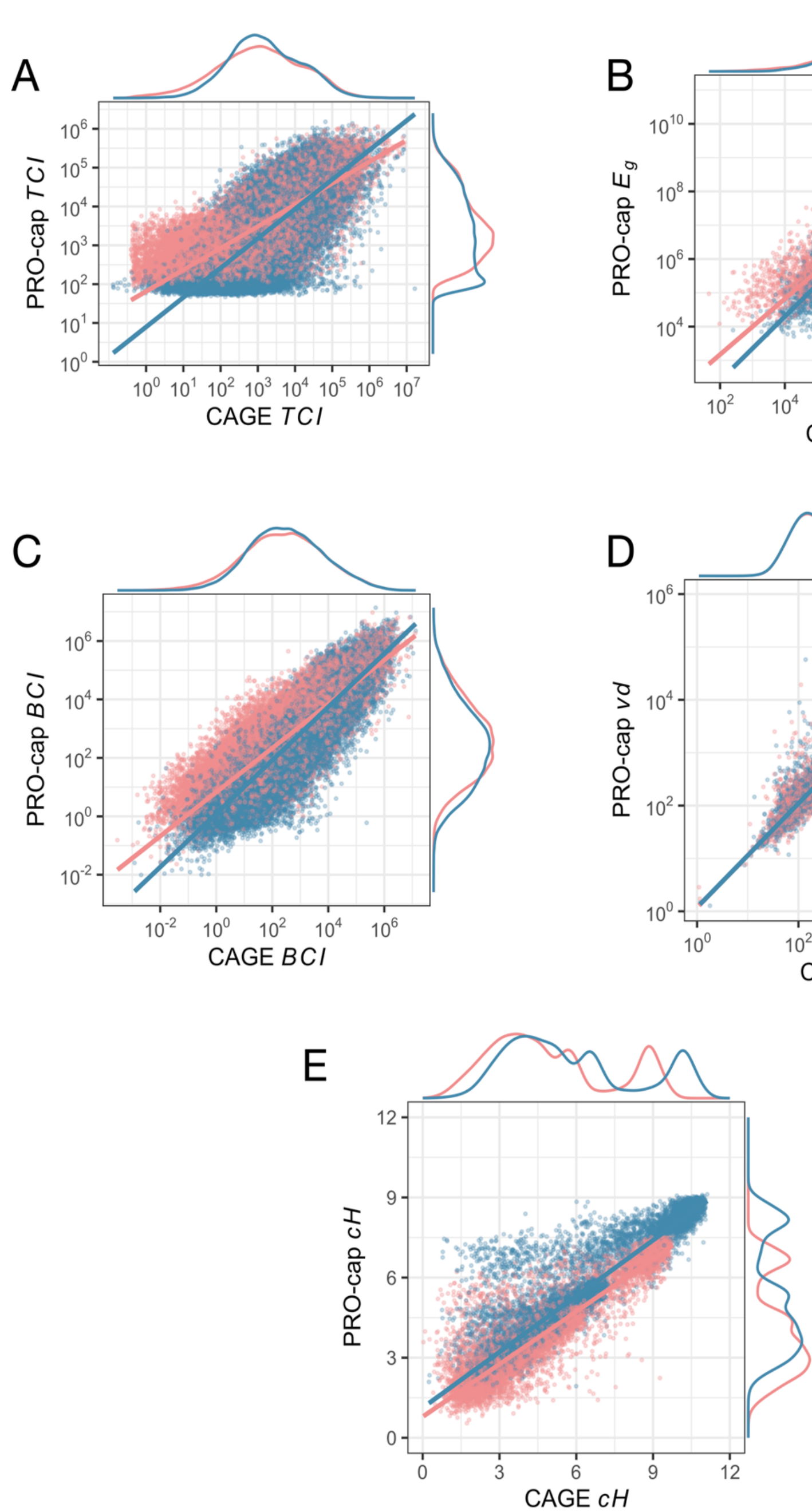

Supplementary Figure 31. Defining parameters of TSS deployments obtained through CAGE or PRO-cap compared for 52185 common TSS sets. A–E: X–axes show CAGE–derived values and Y–axes show PRO-cap–derived values. At lower values of TCI (**A**), Eg (**B**) and BCI (**C**) the higher signal in *C* through PRO-cap is evident as the pink trendlines representing *C* deviate towards higher Y–axis values unlike the blue trendline representing *N*. This difference is mitigated when the parameter vd (**D**) and cH (**E**) are compared showing the robustness of these parameters (and by extension, of vd/cH as well) for a cross–technique comparison between *C* and *N*. **A–F**; Wilcoxon rank–sum test, p–value ≤ 1.72e-6.

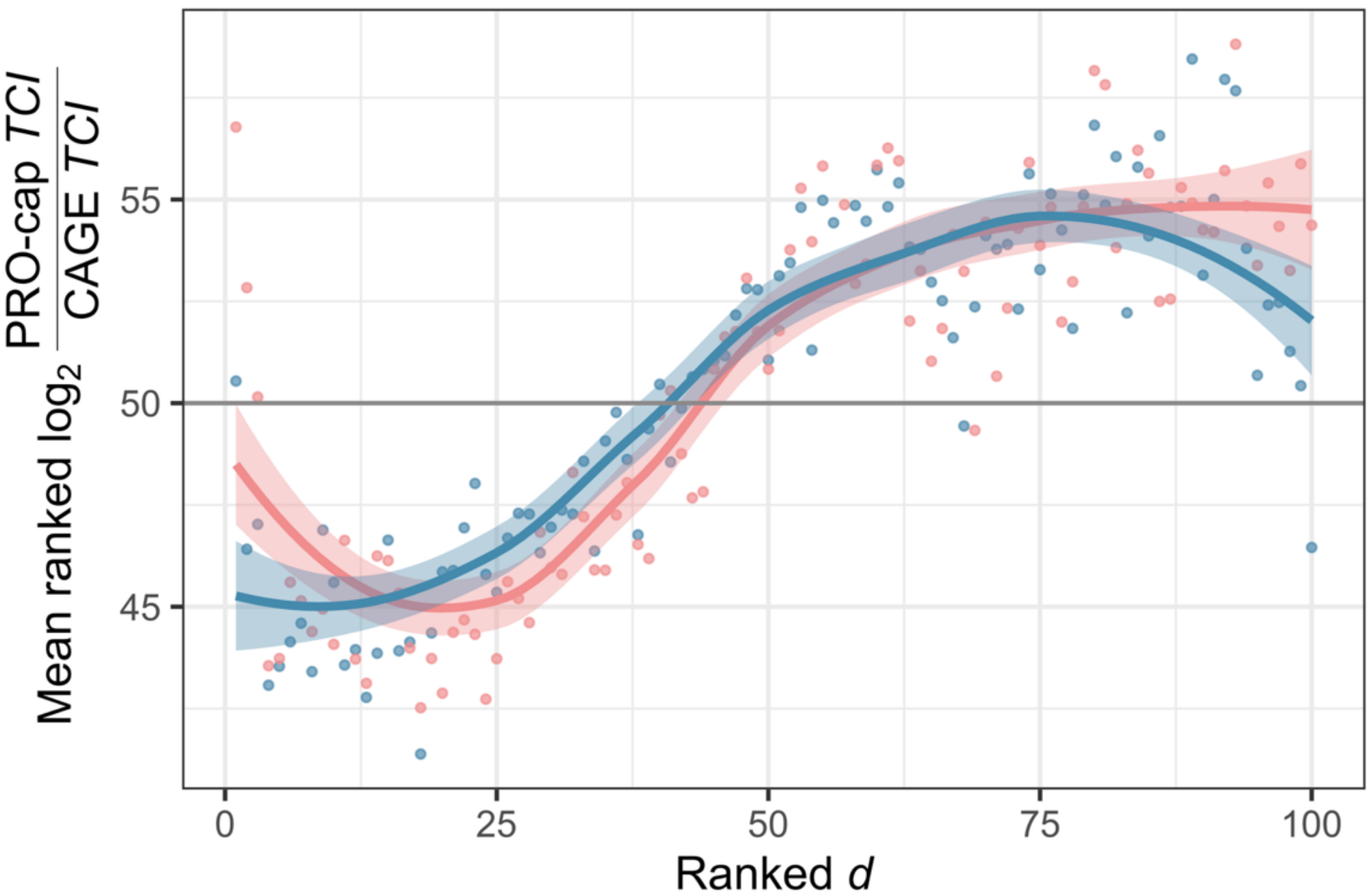

Supplementary Figure 32. Log₂ ratios of PRO-cap and CAGE *TCI* and ranked d show d–dependent TSS capture differences between the two techniques: A comparison of PRO-cap and CAGE signals at varying lengths of d is shown by plotting ranks of d (X–axis; d values and ranks both ordered ascending across 100 bins) against $\log_2$ ratio ranks (100 bins) of TCI values from PRO-cap and CAGE (Y–axis). The relationship patterns for C (pink) and N (blue) are depicted by a LOWESS fit ± 95% CI. The C and N values differ significantly (Wilcoxon rank–sum test, p–value ≤ 1.72e-6) showing that PRO-cap and CAGE have d-dependent differences in TSS activity capture between C and N. Notably, the termini of the LOWESS fits for C and N deviate strongly with PRO-cap showing a better capture at very low as well as very high d as compared to CAGE.

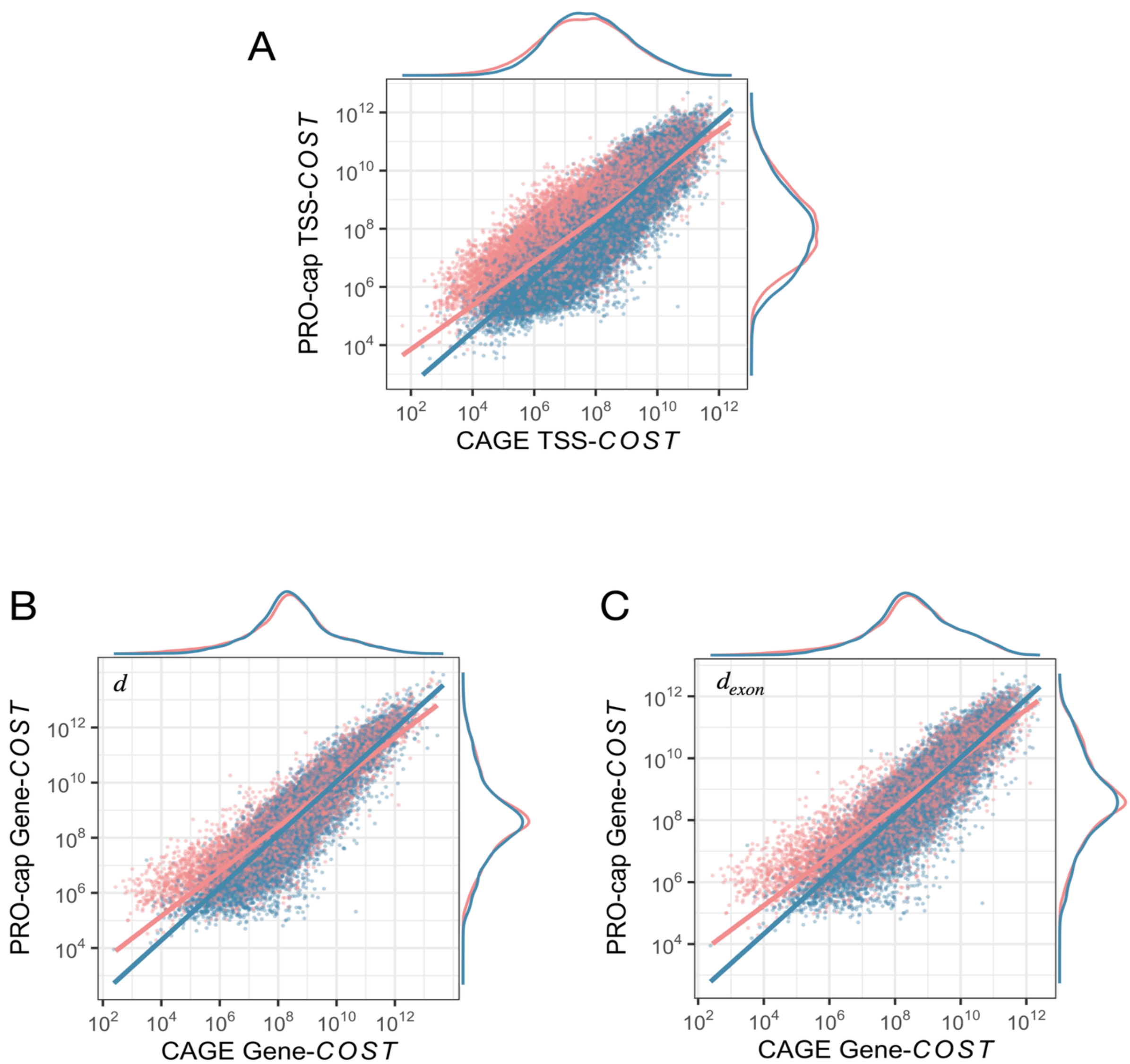

Supplementary Figure 33. TSS and Gene-level costs without and with introns. A: $COST$ calculated for entire d (introns ignored) at TSS level using PRO-cap or CAGE datasets show correlated values between the two techniques. However, a significantly deeper capture of low activity TSSs is evident in PRO-cap as compared to CAGE specifically for cancer samples. **B and C:** Scatter plots show relationship between PRO-cap and CAGE derived $COST$ values when the TSS level $COST$ values are cumulated gene–wise either ignoring introns for entire d **(B)** or by splicing out one intron at a time for each d (d_{exon}) **(C)**. The stronger capture of low activity TSSs through PRO-cap in cancers is further accentuated at gene level for d as well as d_{exon}. **A–C:** For all comparisons (either CAGE or PRO-cap, and TSS–level or gene–level) $COST_C > COST_N$ except TSS–level CAGE (Wilcoxon signed–rank test, p–value ≤ 2.2e-16).

2 Supplementary Tables

The supplementary tables are available as TSV files.

Table S1. GRCh38–hg38 genomic coordinates of the region between the first base of a TSS of a gene (CAGE–derived) and the first base of the most upstream start codon for that gene (GENCODE–derived): The length of this region is marked as d for that corresponding TSS in all subsequent tables denoting the distance (in bp) of the TSS from its start codon. TSSs with the same ID and overlapping coordinates were merged. TSS IDs follow the nomenclature as available in FANTOM CAGE data.

Table S2. TSS–level distance parameters $\boldsymbol{d}$, incremental $\boldsymbol{d}$ and fractional $\boldsymbol{d}$: d – the distance of the TSS from its corresponding start codon (as described in table S1, incremental d – the ratio of d increment for a (distal) TSS from its penultimate (proximal) TSS and d of the penultimate TSS, and, fractional d – the ratio between the d of a TSS and the highest d for a TSS for the same gene.

Table S3. Genome–encoded gene–level $\boldsymbol{d}$: Harmonic mean of d (HMd) for 15861 genes that have curated TSSs common in *N* as well as *C* were derived. Only d–values common to *C* and *N* were considered.

Table S4. CAGE samples from FANTOM5 with sample identities as mentioned in FANTOM, curated sample number appendix with a binary $\boldsymbol{N}$ (non–cancer) or $\boldsymbol{C}$ (cancer) sample ID. The sample indices are traceable to actual sample metadata in table S5.

Table S5. CAGE raw score matrix for all the samples: The first column header is TSS name (in the format *pn@GENE*), followed by 1255 columns with non–cancer CAGE scores and 476 columns with cancer CAGE scores and the last column contains the TSS d value. Sample names correspond to sample IDs as mentioned in table S4. CAGE raw scores include only those TSSs which were common to all *N* and *C* samples. The table is accessible through the following link: https://usegalaxy.org/api/datasets/f9cad7b01a4721357b7e32ed2db2c7a8/display?to_ext=tsv

Table S6. Transcript Count Index ($\boldsymbol{TCI}$) in $\boldsymbol{N}$ ($\boldsymbol{TCI_N}$) and $\boldsymbol{C}$ ($\boldsymbol{TCI_C}$) and TSS-level $\boldsymbol{E}$ ($\boldsymbol{E_t}$) in $\boldsymbol{N}$ ($\boldsymbol{E_{tN}}$) and $\boldsymbol{C}$ ($\boldsymbol{E_{tC}}$). The values are for 55048 CAGE TSSs as indexed in tables S4 and S5.

Table S7. Gene–level $\boldsymbol{E}$ for N ($\boldsymbol{E_{gN}}$) and C ($\boldsymbol{E_{gC}}$). The values are provided for 15861 genes to which the 55048 TSSs map.

Table S8. Base Count Index ($\boldsymbol{BCI}$) in $\boldsymbol{N}$ ($\boldsymbol{TCI_N}$ and $\boldsymbol{BCI_N}$) and $\boldsymbol{C}$ ($\boldsymbol{TCI_C}$ and $\boldsymbol{BCI_C}$). The values are for 55048 CAGE TSSs as indexed in tables S4 and S5.

Table S9. Gene–level vd for *N* (vd_N) and C (vd_C). The values are provided for 15861 genes to which the 55048 TSSs map.

Table S10. Gene–space entropies (gH) of 11928 genes for *N* (gH_N) and C (gH_C) and the C/N ratio of the entropies.

Table S11. Sample–space entropies (sH) for N (sH_N) and C (sH_C) and the C/N ratio of the entropies. sH for each TSS was calculated in two different ways. sH from non–zero scores: sH for a TSS from randomly selected 100 samples such that the 100 samples have a score greater than 0 in *N* as well as *C* and their sH_C/sH_N . TSSs that did not satisfy these criteria could not have their sH calculated and are marked NA. sH from all scores: sH for a TSS from randomly selected 100 samples in *N* and *C* and their $\log_2$ ratios. TSSs that could be used to calculate sH in both ways were considered *Frequently Deployed* while the other TSSs were labelled *Non–Frequently Deployed*.

Table S12. Compound entropy (cH) of 11928 genes for *N* (cH_N) and C (cH_C) and the vd/cH ratios of the entropies for *N* (vd_N/cH_N) and C (vd_C/cH_C).

Table S13. sH interpolation using simulated values. Coefficients of the exponential growth function with rate constant parameter for each of the assigned d bins. $y0$ is the offset, A is the initial value and, $R0$ is the rate constant.

Table S14. Tabulation of the GOrilla GO term enrichments for terminal quartile comparisons of vd. Column headers *GO Term*, *Description*, *p–value*, *FDR q–value* and *Enrichment* were obtained directly from the results generated by the software (PMID 19192299 and 17381235 and see methods for details). The header *Assigned term* corresponds to a group of related functional categories. The column *Comparison* indicates the target versus background. Q1 and Q4 correspond to the quartiles 1 and 4 respectively when sorted by vd_C/vd_N . *Ontology* indicates the biological process (BP) or molecular function (MF) categories.

Table S15. Tabulation of the GOrilla GO term enrichments for terminal quartile comparisons of gH. Column headers *GO Term*, *Description*, *p–value*, *FDR q–value* and *Enrichment* were obtained directly from the results generated by the software (PMID 19192299 and 17381235 and see methods for details). The header *Assigned term* corresponds to a group of related functional categories. The column *Comparison* indicates the target versus background. Q1 and Q4 correspond to the quartiles 1 and 4 respectively when sorted by gH_C/gH_N . *Ontology* indicates the biological process (BP) or molecular function (MF) categories.

Table S16. Tabulation of the GOrilla GO term enrichments for terminal quartile comparisons of sH. Since sH is a TSS–level parameter, the TSSs segregating into the two quartiles were grouped by their specific genes. Column headers *GO Term*, *Description*, *p–value*, *FDR q–value* and *Enrichment* were obtained directly from the results generated by the software (PMID 19192299 and

17381235 and see methods for details). The header *Assigned term* corresponds to a group of related functional categories. The column *Comparison* indicates the target versus background. Q1 and Q4 correspond to the quartiles 1 and 4 respectively when sorted by sH_C/sH_N . *Ontology* indicates the biological process (BP) or molecular function (MF) categories.

Table S17. Tabulation of the GOrilla GO term enrichments for terminal quartile comparisons of vd/cH. Column headers *GO Term*, *Description*, *p–value*, *FDR q–value* and *Enrichment* were obtained directly from the results generated by the software (PMID 19192299 and 17381235 and see methods for details). The header *Assigned term* corresponds to a group of related functional categories. The column *Comparison* indicates the target versus background. Q1 and Q4 correspond to the quartiles 1 and 4 when sorted by $\log_2$ ratio of vd_C/cH_C and vd_N/cH_N. *Ontology* indicates the biological process (BP) or molecular function (MF) analysis.

Table S18. Curated PRO-cap sample list. The table lists GSE and ENCODE identifiers of the samples and associated experiments for all the PRO-cap samples used for validatory analyses. Based on metadata the samples were curated and classified as *C* or *N*. PRO-cap samples indexed for usage in various statistical analyses are henceforth referred to as "PRO-cap samples".

Table S19. Scaled and normalized PRO-cap TSS activity scores. For 52185 TSSs common to CAGE and PRO-cap *C* and *N* samples, tag counts mapping to CAGE TSS peaks. Read counts obtained from independent PRO-cap experiments were scaled down to the *N* sample with the lowest non–zero score. The scaled scores were quantile normalized and used for calculations of TSS deployment parameters. These scaled and normalized values are listed. The table is accessible through the following link:
https://usegalaxy.org/api/datasets/f9cad7b01a4721352821d24d830913a2/display?to_ext=tsv

Table S20. Comparison summary of TSS and gene level parameters derived using CAGE and PRO-cap experiments. Multiple parameters were derived using PRO-cap data (TSS count 52904) as they were derived using CAGE data (TSS count 55048) and comparisons were performed between *C* and *N*. The table lists the parameters and the changes observed between *C* and *N*. Additionally, these comparisons were also done by restricting to the common TSS set of 52185 between PRO-cap (*N* and *C*) and CAGE (*N* and *C*).

Table S21. The parameter vd/cH obtained for *C* and *N* using PRO-cap data for 11513 genes.

Table S22. Tabulation of the GOrilla GO term enrichments for terminal quartile comparisons of PRO-cap derived vd/cH. Column headers *GO Term*, *Description*, *p–value*, *FDR q–value* and *Enrichment* were obtained directly from the results generated by the software (PMID 19192299 and 17381235 and see methods for details). The header *Assigned term* corresponds to a group of related functional categories. The column *Comparison* indicates the target versus background. Q1 and Q4 correspond to the quartiles 1 and 4 when sorted by $\log_2$ ratio of vd_C/cH_C and vd_N/cH_N From PRO-cap data. *Ontology* indicates the biological process (BP) or molecular function (MF) analysis.

Table S23. List of common PRO-cap and CAGE TSSs with GENCODE introns used for generating unitary splice variants of $\boldsymbol{d}$. Each TSS ID has a d and one d_{exon} value calculated per intron. These values of d and d_{exon} are used for $COST$ calculations.

Table S24. Sample–wise $\boldsymbol{COST}$ of Aggregated Promoterome ($\boldsymbol{CAP}$) for CAGE samples. The $COST$ values have been calculated using only d or d as well as d_{exon} values.

Table S25. Sample–wise $\boldsymbol{COST}$ of Aggregated Promoterome ($\boldsymbol{CAP}$) for PRO-cap samples. The $COST$ values have been calculated using only d or d as well as d_{exon} values.

Table S26. Tabulation of the GOrilla GO term enrichments for terminal quartile comparisons of gene–level $\boldsymbol{COST}$ values. Column headers *GO Term*, *Description*, *p–value*, *FDR q–value* and *Enrichment* were obtained directly from the results generated by the software (PMID 19192299 and 17381235 and see methods for details). The header *Assigned term* corresponds to a group of related functional categories. The column *Comparison* indicates the target versus background. Q1 and Q4 correspond to the quartiles 1 and 4 when sorted by $COST$ values. *Ontology* indicates the biological process (BP) or molecular function (MF) analysis.

Table S27. Summary of all statistical analyses performed with references to p–values, corresponding false discovery rate (FDRs) calculation details and references to the corresponding figures and tables.
