# Supplementary material for: Gene functions determine stochastic or adaptive futile transcription in cancers through deregulated start site deployments
Source: Front Bioinform. 2026 Oct 14;6:1883461. doi: 10.3389/fbinf.2026.1883461 (PMC13713664; doi:10.3389/fbinf.2026.1883461)
Supplement: Supplementary file 2 [file DataSheet3.pdf]

Supplementary Material

1 Gene and TSS annotation of CAGE data

hg38 CAGE peaks from UCSC and Riken databases were used as reference for annotating CAGE peak coordinates of samples (see Section 2.1) to their corresponding genes. We used the start coordinates of the CAGE peaks instead of individual CTSSs as the location of TSS; references to TSSs in this work are CAGE peak start coordinates. Closely spaced overlapping peaks were subsumed to a single merged peak. Additionally, all peaks mapping to the mitochondrial chromosome were excluded.

The UCSC hg38 CAGE peak data, available in the BigBed format, was downloaded and converted to BED6 format with 209911 coordinates in all. 102235 of the coordinates (including the mitochondrial chromosome) had the peaks annotated to a gene in the format “p<serial>@<HGNC/MGI/Entrez gene name>” as described elsewhere (32). The remaining 107676 coordinates were labelled with peak accession “hg_<serial_num>.<version_num>” as described in (61). To maximize the number of annotated TSSs that could be used for this analysis, we tried to determine the annotations of the remaining TSSs using Riken database. We used a combination of the Riken hg38 and hg19 robust promoters for this. The Riken hg38 robust promoters had 201295 CAGE peaks with CAGE peak IDs that included the peak accession “hg_<serial_num>.<version_num>” and the corresponding coordinates in the hg19 genome assembly (For example – hg19::chr1:564639..564649,+;hg_2.1). The gene annotated peaks were available in Riken’s hg19 robust promoter set (n = 201291).

The peak accession from the UCSC set mapped to 101847 peaks in the Riken hg38 robust promoter set. The Riken hg38 set had genomic coordinates for the peaks in hg38 and hg19 assemblies. Since the Riken hg19 promoter set had the peak identities, we mapped the 101847 peaks to the Riken hg19 set. 9125 peaks successfully mapped back to the hg19 assembly and were included with the remaining 98948 peaks (excluding the mitochondrial chromosome) with gene–annotated identities.

Some of the CAGE peak coordinates had multiple TSSs annotated to the same location. The databases had stored such locations with all TSSs separated by a “,”. We split these IDs into individual entries with the same coordinates. We retained these locations since in many cases the additional coordinates were mapping to non-coding RNA which would be eventually filtered out in the downstream steps.

Schematic 1 summarizes the pipeline followed to pre–process the FANTOM5 CAGE peak data for annotating TSSs with their genes. The databases used are underlined. The number of CAGE peaks downloaded from each database is mentioned as “n”. All file formats are written below the data in italics. The header of each file is mentioned in courier font with the genome assembly marked on the left within square brackets and colour–coded (blue for hg38 assembly and yellow for hg19 assembly). TSS IDs were available in two forms: “pn@GENE” is highlighted in green and “hg_<num>.<num>” is highlighted in red.

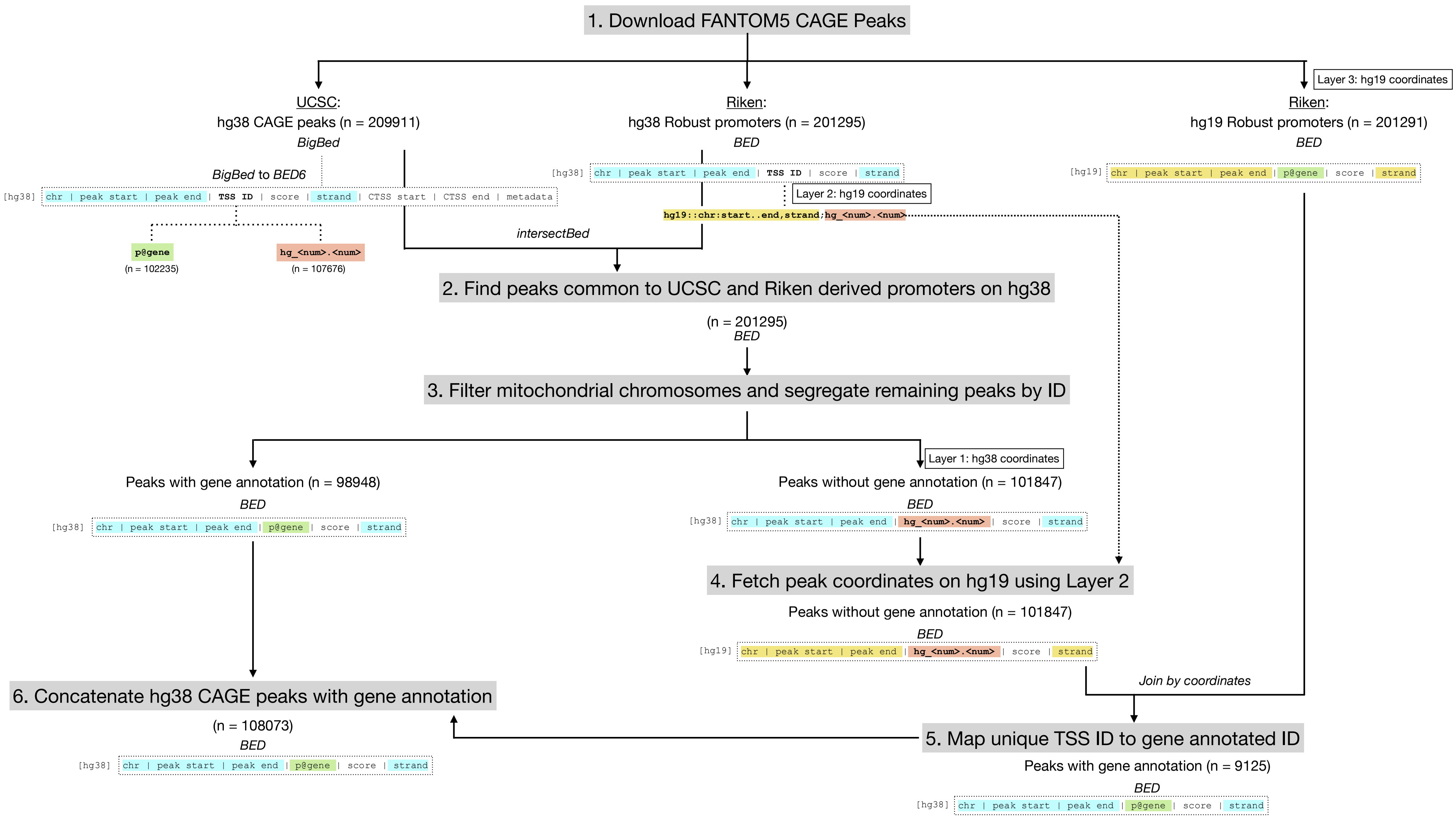

Schematic 1: Pre–processing FANTOM5 CAGE peak data and mapping their TSS annotations.

Note: "n" is the number of CAGE peaks. We consider start coordinates of these peaks are TSSs

2 Curation of FANTOM5 CAGE sample data

The FANTOM5 consortium has profiled 1816 human samples through phases 1 and 2 and these include 286 cell lines, 21 fractionations and perturbations, 561 primary cells, 783 time courses and 183 tissues. The consortium also provides technical replicates for 10 samples. We treated replicates (biological and technical) as independent samples. Thus, a total of 1826 individual CAGE tag start site (CTSS) files were available for the analysis. The 1826 samples aligned to the human reference genome GRCh38 were available in two formats – the raw read counts post-alignment were available in the CTSS BigWig files or the library size normalized TPM BigWig files. We used the tag count and RLE normalized TPM files for determining the deployment.

The 3652 TPM files were downloaded keeping the sample file names intact but replacing all special characters with an underscore for easier processing and to avoid mismatches when mapping with the metadata. Samples were classified as "Cancers (*C*)" if the keywords "cancer", "tumor", "oma", "leukemia" were present in the title and the remaining were labelled "Non–caners (*N*)". Some exceptions were manually corrected. These include "stomach fetal donor" that contains the pattern "oma" but is actually a non–cancer. Samples associated with the embryo such as embryonic stem cells (51 samples) and embryoid body cells (44 samples) were excluded from the analyses (95 samples excluded). Embryonic cell lines, however, were retained and segregated as non–cancers (embryonic kidney, pancreas and mesenchymal cell lines) while the embryonic carcinoma cell line was retained as cancer. The final number of samples used in the analyses include 1731 with 1255 non–cancers and 476 cancers.

All 3462 BigWig files were converted to BED format. The distal end of the CAGE peak coordinate with respect to the direction of transcription were assigned the TSS for the peak. The promoter annotations for the coordinates in the two BED files for each sample (forward and reverse strands) were obtained by mapping them to the CAGE peaks with gene annotations from databases (see Supplementary Materials, Section 1). The TPM values from overlapping coordinates of the same peak were summed (CAGE raw scores henceforth) and the two strands were concatenated to a single file for a sample. Non–cancers had 56394 TSSs while cancers had 55066 TSSs with 55048 TSSs commonly represented in at least one cancer and one non–cancer sample.

Schematic 2 summarizes download, curation and classification of the FANTOM5 CAGE sample TPM data. The databases used are underlined. All file formats are written below the data in italics. The header of the file is mentioned in courier font with the genome assembly marked on the left within square brackets.

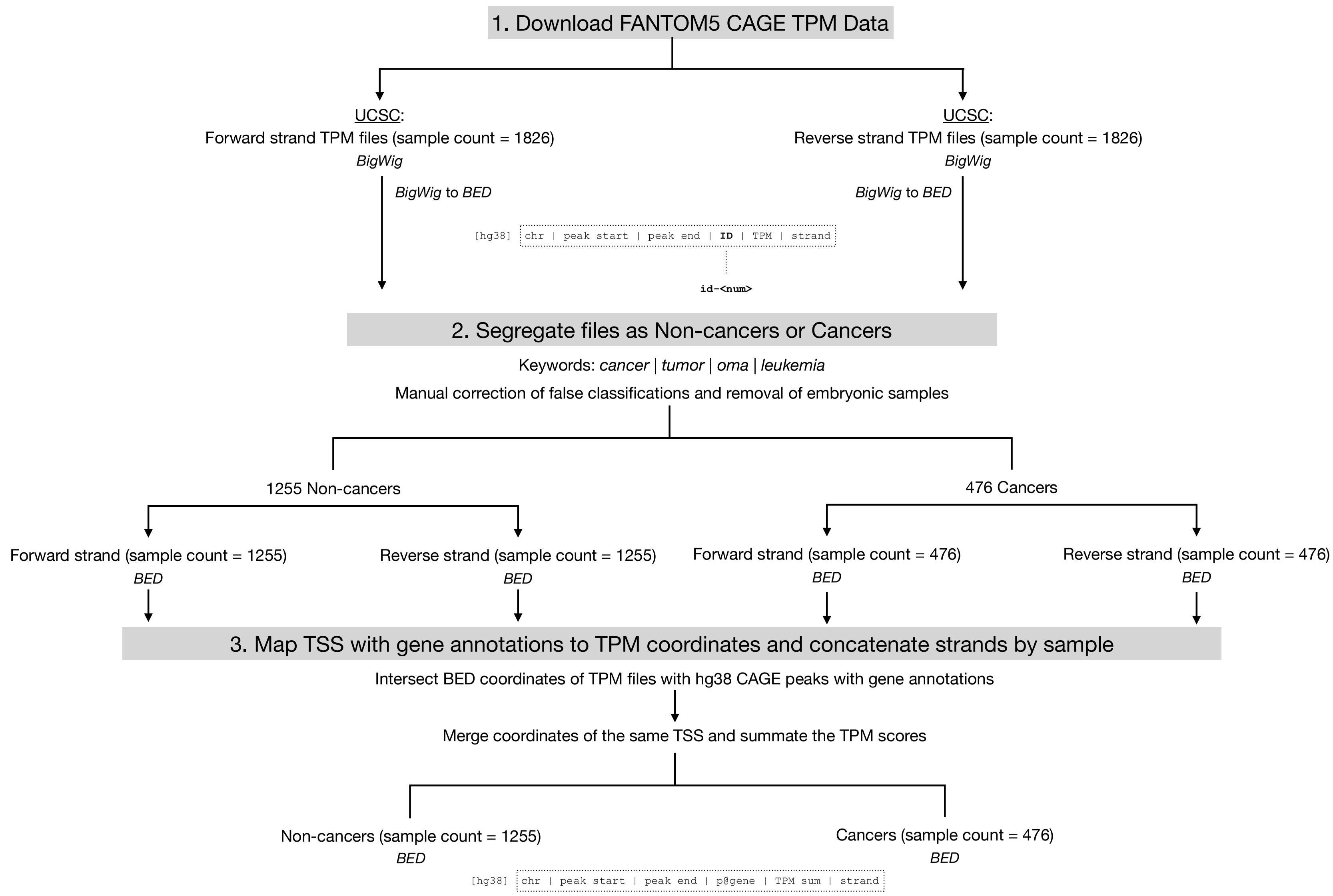

Schematic 2: Downloading, curating and classifying FANTOM5 CAGE samples TPM data.

Note: Each sample reports different number of TSSs

3 Estimating TSS distance (d) and its derivatives

The length of the region between a transcription start site and the most upstream translation start site of the same gene is the distance for that TSS. The length of this region is calculated using the genomic coordinates of the TSS (see Supplementary Materials, Section 1) and the start codon in a strand–specific manner.

Schematic 3 shows the pipeline for estimating d for a TSS on each strand. The file formats are written below the data in italics. The TSS coordinates for the derivation were obtained from the hg38 CAGE peaks with gene annotation (see Supplementary Material, Section 1) and the most upstream start codon from the GENCODE file. TSSs which initiate downstream of the most upstream start codon are not included. After eliminating mitochondrial genes and pseudo autosomal regions, 20072 gene–start codons were mapped to the 108073 TSSs by their genes. Of the total 57461 TSSs for which a d was measured, 29136 initiate from the plus strand and 28325 from the minus strand.

A simplified graphic of the genome is shown to illustrate the strand–wise measurement of d. The CAGE peak is marked in teal with peak coordinates marked with respect to the plus strand only. The vertical teal line represents the peak summit. The distal end of the CAGE peak is considered the TSS and marked with arrow labelled "pn@GENE". The most upstream start codon associated with the gene is highlighted by a green box. The TSS–d region is marked by a grey box with dashed border.

These 57461 TSS–d values were eventually subset to either 55048 TSSs (common to CAGE *N* and *C*) samples or 52904 (common to PRO-cap *N* and *C* samples). The remaining 2413 TSSs which were not included in the analyses belonged to the samples that were excluded and hence not reported in either the *C* or the *N* data.

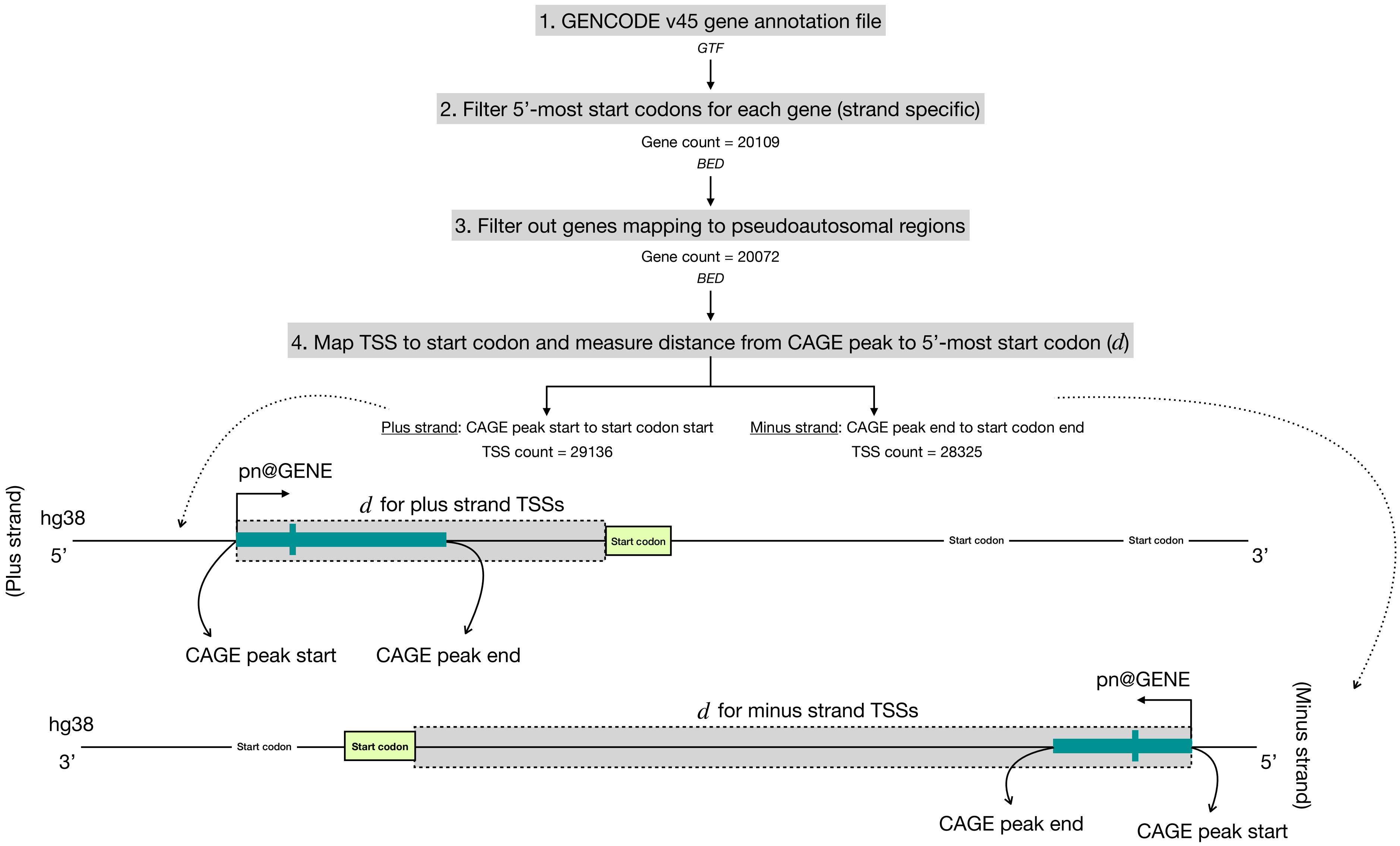

Schematic 3: Pipeline for measuring the TSS–*d*.

The following parameters were derived using the TSS distance (d) parameter. The derivation of these parameters requires gene annotations and remain constant across samples since they are genome encoded.

3.1 Gene–level distance ($\boldsymbol{HMd}$)

For gene–level comparisons, the distances of all TSSs for a gene had to be summarized by a single value for the gene. We used the harmonic mean of all TSSs for a gene as the gene–level distance since it provided a higher weight for smaller values. This way the gene-level distance distribution was closer to that of the TSS–level distance with stronger influence from the proximal TSSs.

$$HMd_g = \frac{|\tau|}{\sum_t^\tau \frac{1}{d_t}} \tag{I}$$

Where,
HMd_g is the harmonic mean of d for a gene g.
d_t is the distance of TSS t from the start codon of its gene g.
t is a TSS in the gene–space for gene g.
τ is the set of all TSSs in the gene–space for gene g.
$|\tau|$ is the number of TSSs in the gene–space for gene g.

3.2 Incremental d

Inter–TSS comparison for a gene was measured as the increase in distance for a TSS with respect to its previous TSS (adjacent and proximal to the start codon in the pair). Incremental d was calculated for each gene as a ratio of the d difference between two consecutive TSSs of a gene and the lower d in the pair.

$$d_i = \frac{d_u - d_p}{d_p} \tag{II}$$

Where,
d_i is the incremental d for the pair.
d_u is the distance of the longer TSS of the pair.
d_p is the distance of the TSS immediately downstream to d_u.

3.3 Fractional d

The ratio of the TSS–d and the most distal TSS for the same gene–space g.

$$d_f = \frac{d_t}{d_{max}} \tag{III}$$

Where,
d_f is the fractional d for a TSS t in its gene–space.
d_t is the actual d for the TSS t.
d_{max} is the d of the most distal TSS in the same gene–space.

4 Normalizing and scaling of CAGE scores

The raw CAGE scores were either normalized or scaled according to the analyses. Normalization of a TSS in a sample was done as the share of that score in the entire promoterome independently for *N* and *C*.

$$score_{t,s} = \frac{raw\ score_{t,s}}{\sum_{t=1}^{55048} \sum_{s=1}^{S} raw\ score_{t,s}} \qquad (IV)$$

Where,
$score_{t,s}$ is the normalized score of TSS t in sample s.
$raw\ score_{t,s}$ is the raw score of the TSS t in sample s.
t is the TSS.
s is a sample in the sample set S.
S is the cancer or non–cancer sample set.

Scaling, however, was performed commonly for both *N* and *C*. A scaling factor was estimated for each sample such that the sum of scores for each sample would be equal after scaling.

$$\gamma_{t,s} = raw\ score_{t,s} * f_s \qquad (V)$$

Where,
$\gamma_{t,s}$ is the scaled score of TSS t in sample s.
$raw\ score_{t,s}$ is the raw score of TSS t in sample s.
f_s is the scaling factor for sample s.
t is the TSS.
s is the sample in the sample set S.
S is the cancer or non–cancer sample set.

Note: All parameters derived from the raw score, normalized score or scaled scores are dependent on the sample set of the parameter. All the parameters described below would therefore contain a value in *N* and one in *C*. The sample (s) or sample set (S) identities, although not mentioned for brevity, are implied unless mentioned otherwise.

5 Transcript Count Index (TCI) of a TSS

The TCI is a population parameter that is calculated as the share of TSS' score across all the samples in the promoterome of that sample set, scaled by 1e9. Since the scores are normalized, the TCI is simply calculated from the sum of normalized scores for a TSS across all samples. The share of each TSS in the calculation of TCI suffers from a large denominator (~55000 TSSs x CAGE score per transcript x ~1000 samples) ranging in tens of millions. Simply for convenience of calculation and data visualization, we used a convenient universal common multiplication factor of 10^9.

$$TCI_t = \sum_{s}^{S} score_{t,s} * 10^9 \qquad (VI)$$

Where,
TCI_t is the transcript count index of TSS t in the sample set S.
$score_{t,s}$ is the normalized score of the TSS t in sample s.

s is a sample in the sample set S.
S is the cancer or non-cancer sample set.

A total of 55048 TCIs (one for each TSS) was obtained for *N* and *C* each. The maximum and minimum TCI at every d were calculated as TCI_{max} and TCI_{min} respectively.

6 Energy cost coefficient of transcription (E)

We summarize the energy cost coefficient of transcription of a TSS or its parent gene in the following ways:

6.1 TSS-level E (E_t)

The TSS–level E is a population parameter for each TSS from all its samples in a population. It is calculated as the number of times the d is transcribed.

$$E_t = (TCI * d)_t \qquad (VII)$$

Where,
E_t is the cost of transcription from the TSS t.
TCI_t is the transcript count index of the TSS t.
d_t is the distance of the TSS t from the ATG of its corresponding gene.

6.2 Gene-level E (E_g)

The gene–level E is a population parameter for each gene from all its samples in a population. It is calculated as the total energy cost of transcribing a gene from each of its TSSs.

$$E_g = \sum_{t}^{\tau} (TCI * d)_t \qquad (VIII)$$

Where,
E_g is the total cost of transcribing the gene g from all its TSSs.
t is a TSS in the gene–space for gene g.
τ is the set of all TSSs in the gene–space for gene g.
TCI_t is the transcript count index for the TSS t in the gene–space for gene g.
d_t is the distance of the TSS t from the start codon of its corresponding gene g.

7 Base Count Index (BCI)

The population parameter BCI for each TSS was calculated as the share of E of a TSS across all samples within the sample set, scaled by 1e9. The share of each TSS in the calculation of TCI suffers from a large denominator (~55000 TSSs x CAGE score per transcript x distance per TSS in bases x ~1000 samples) ranging in billions. Simply for convenience of calculation and data visualization, we used a convenient universal common multiplication factor of 10^9.

$$BCI_t = \sum_{s}^{S} (score_s * d)_t * 10^9 \qquad (IX)$$

Where,
$BCI_{t,S}$ is the base count index of TSS t in population S.
$score_{t,s}$ is the normalized score of the TSS t in sample s.
d_t is the distance of the TSS t from the start codon of its corresponding gene g.
s is a sample in the sample set S.
S is the *C* or *N* sample set.

8 Effective d (vd)

The effective d can be both a sample–level and population level value for each gene. The effective d at population level is calculated as the TCI–weighted arithmetic mean of d while at the sample level, it is the score–weighted arithmetic mean of d.

8.1 Population-level effective d

$$vd_g = \sum_{t}^{\tau} \frac{(TCI * d)_t}{\sum_t^\tau TCI_t} \qquad (X)$$

Where,
$vd_{g,S}$ is the effective distance of gene g in population S.
$TCI_{t,S}$ is the transcript count index of TSS t in population S.
d_t is the distance of TSS t from the start codon of its gene g.
t is the TSS in the gene–space for gene g.
τ is the set of all TSSs in the gene–space for gene g.
S is the *C* or *N* sample set.

8.2 Sample-level effective d

$$vd_g = \frac{\sum_t^\tau (score * d)_t}{\sum_t^\tau score_t} \qquad (XI)$$

Where,
vd_g is the effective distance of gene g in sample s.
$score_t$ is the normalized score of TSS t in sample s.
d_t is the distance of TSS t from the start codon of its gene g.
t is the TSS in the gene–space for gene g.
τ is the set of all TSSs in the gene–space for gene g.
s is the sample in sample set S.
S is the *C* or *N* sample set.

Note: vd is always assumed at the population–level in this work except when performing the PCAs.

9 Gene–space entropy (gH)

The gene–space entropy was calculated only for those genes which had at least two TSSs. We define the gene–space through the probability of a TSS deployment across all samples of the population with respect to the activities of all the TSSs of that gene.

$$P_g(t) = \frac{\sum_s^S \gamma_{t,s}}{\sum_t^\tau \sum_s^S \gamma_{t,s}} \qquad (XII)$$

Where,
$P_g(t)$ is the probability of occurrence of the TSS t for gene g.
$\gamma_{t,s}$ is the scaled score of the TSS t in sample s.
t is a TSS in the gene–space for gene g.
τ is the set of all TSSs in the gene–space for gene g.
s is a sample in the sample set S.
S is the *C* or *N* sample set.

We then derive the gene–space entropy as the Shannon entropy of the probabilities of all the TSSs of the gene. Shannon entropy measures the information in bits or binary transfer of information; The formula for entropy thus uses a base 2 in the logarithm. The gene–space, however, allows only one of the τ set of TSSs to be deployed at any point of time. Thus, given that the gene is transcribed, it can be transcribed in as many ways as the number of TSSs of that gene (also represented as $|\tau|$). The gene–space entropy thus uses a logarithm base equal to the number of TSSs contributing to that gene–space.

$$gH_g = -\sum_t^\tau P_g(t) * \log_{|\tau|} P_g(t) \qquad (XIII)$$

Where,
gH_g is the gene–space entropy for a given gene g in sample set S.
$P_g(t)$ is the probability of deployment of TSS t within its gene–space g.
t is the TSS in the gene–space for gene g.
τ is the set of all TSSs of the gene–space for gene g.

10 Sample–space entropy (sH)

Stochastic deployment of TSSs in the *N* and *C* populations across CAGE profiles was calculated using Shannon entropy for the sample–space of the TSS. Since entropy is a measure of the probability of occurrence, and the CAGE scores for a TSS is a direct measure of the number of times the TSS was deployed, we used the ratio of the scaled scores and number of samples in which the TSS was deployed as the probability equivalent in the sample–space entropy calculation. Since some TSSs could be represented only by a small proportion of the samples and there is an unequal sample count in the *N* and *C* sample sets, we considered only those TSSs that had a CAGE score in at least 100 non–cancer and 100 cancer samples.

$$P_s(t) = \frac{\gamma_{t,s}}{\sum_{s=1}^{100} \gamma_{t,s}} \qquad (XIV)$$

Where,
$P_s(t)$ is the probability of occurrence of the TSS t in its sample–space.
$\gamma_{t,s}$ is the scaled score for the TSS t in sample s.
t is a TSS.

s is a random sample in the sample set S.
S is the *C* or *N* sample set.

The sample–space was then calculated as simply the Shannon entropy with a log–base 2. Since the sampling involves a randomization, we estimate the sample–space entropy as the arithmetic mean of the sample–space entropy calculated through 10 independent iterations of random sampling.

$$sH_t = \frac{\sum_{i=1}^{10}\left(-\sum_{s=1}^{100} P_s(t) * \log_2 P_s(t)\right)}{10} \qquad (XV)$$

Where,
sH_t is the sample–space entropy for TSS t in the sample set S.
$P_s(t)$ is the probability of occurrence of the TSS t in its sample–space.
t is a TSS.
s is a randomly derived sample in the sample set S.
i is the iteration for the random draw.

This mean sample–space Shannon entropy from the 10 iterations shall henceforth be referred to as the sample–space entropy of the TSS. Some TSSs have such low deployment frequencies across samples that the random draw could not a minimum of 100 non–zero scores. We termed these TSSs as "*Non–Frequently Deployed*" and the remaining as "*Frequently Deployed*". The *Frequently Deployed* TSSs were further used to generate a new set of entropy values as described earlier except with only the non–zero scoring samples in *N* and *C* instead of all samples for the 100 randomizations. This filtering of samples was done independently for each TSS in *N* and in *C*.

11 Compound entropy (cH)

We use the product of the TSS probabilities in the sample–space and the gene–space to define the compound probability for that TSS. The gene-space probabilities were calculated as described in the gene–space entropy, however, the sample–space probabilities were calculated using all samples within *N* and *C* instead of just 100 random samples.

$$P_{cH}(t) = P_g(t) * P_s(t) \qquad (XVI)$$

Where,
$P_{cH}(t)$ is the compound probability of TSS t.
$P_g(t)$ is the gene–space probability of TSS t.
$P_s(t)$ is the sample–space probability of TSS t.
t is a *Frequently Deployed* TSS.

The compound entropy was then calculated for each gene similar to the gene–space entropy – with a log–base equal to the number of TSSs in that gene.

$$cH_g = -\sum_{t}^{\tau} P_{cH}(t) * \log_{|\tau|} P_{cH}(t) \qquad (XVII)$$

Where,
cH_g is the compound entropy for a gene g in sample set S.
$P_{cH}(t)$ is the probability of deployment of TSS t within its gene–space for gene g.
t is the TSS in gene–space for gene g.
τ is the set of all TSSs of the gene–space for gene g.
$|\tau|$ is the number of TSSs in the gene.
S is the *C* or *N* sample set.

All score–derived parameters at sample or population level and their hierarchy are described in schematic 4. All file formats are written below the data in italics. The header of the files is mentioned in courier font.

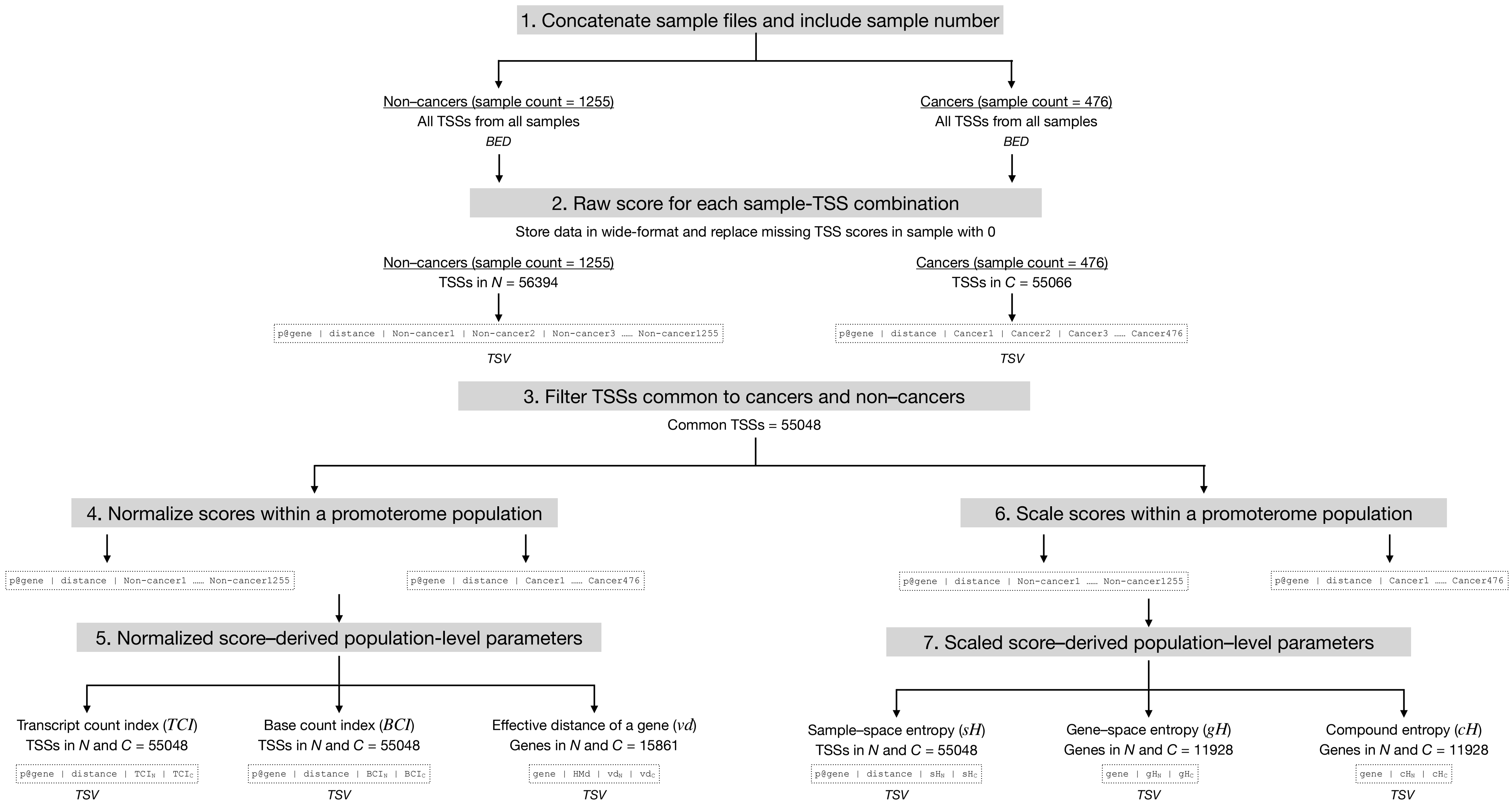

Schematic 4: Summary chart of score derived parameters.

12 PRO-cap data curation

The data for PRO-cap was downloaded from NCBI GEO through R package *GEOquery*. The keywords '(pro[All Fields] AND cap[All Fields] AND ("humans"[MeSH Terms] OR "Homo sapiens"[Organism] OR human[All Fields]) AND encode[All Fields]) AND "Homo sapiens"[porgn]' were used for the query. The GSM BigWigs associated with these keywords and the accompanying metadata including ENCODE ID were fetched. This gave us a total of 425 sample IDs.

The samples were manually curated was "Cancers (*C*)" or "Non–cancers (*N*)" and all excluded all embryo samples. All replicates (biological and technical) were given a unique sample status with the same GSM as its parent appended with _<replicate.number>. The final sample count available for analyses is 415 that includes 305 *N* and 110 *C* samples. The sample classified metadata is available in Supplementary Table S18.

Since the samples were downloaded as bigWig files, the data was processed the same way CAGE data was handled (see Supplementary Materials, Section 2). This was followed by an additional quantile normalization done for PRO-cap (normalization was done only for those TSSs that were present in *N* as well as *C* samples). Since the CAGE data was already library size normalized through TPM in the FANTOM protocol, we restricted the normalization to PRO-cap data only. For the normalization, the *N* sample with the highest number of TSSs having a non–zero score was used as the baseline. All other samples, both *N* and *C*, were quantile normalized with respect to this sample. The normalized score for each TSS of a sample matrix is available in Table S19.

All parameters (as described in Section 2.3) calculated for CAGE data were calculated the same way using PRO-cap data. A pipeline for downloading and pre–processing PRO-cap data is illustrated in schematic 5. All file formats are written below the data in italics. The header of the file is mentioned in courier font with the genome assembly marked on the left within square brackets.

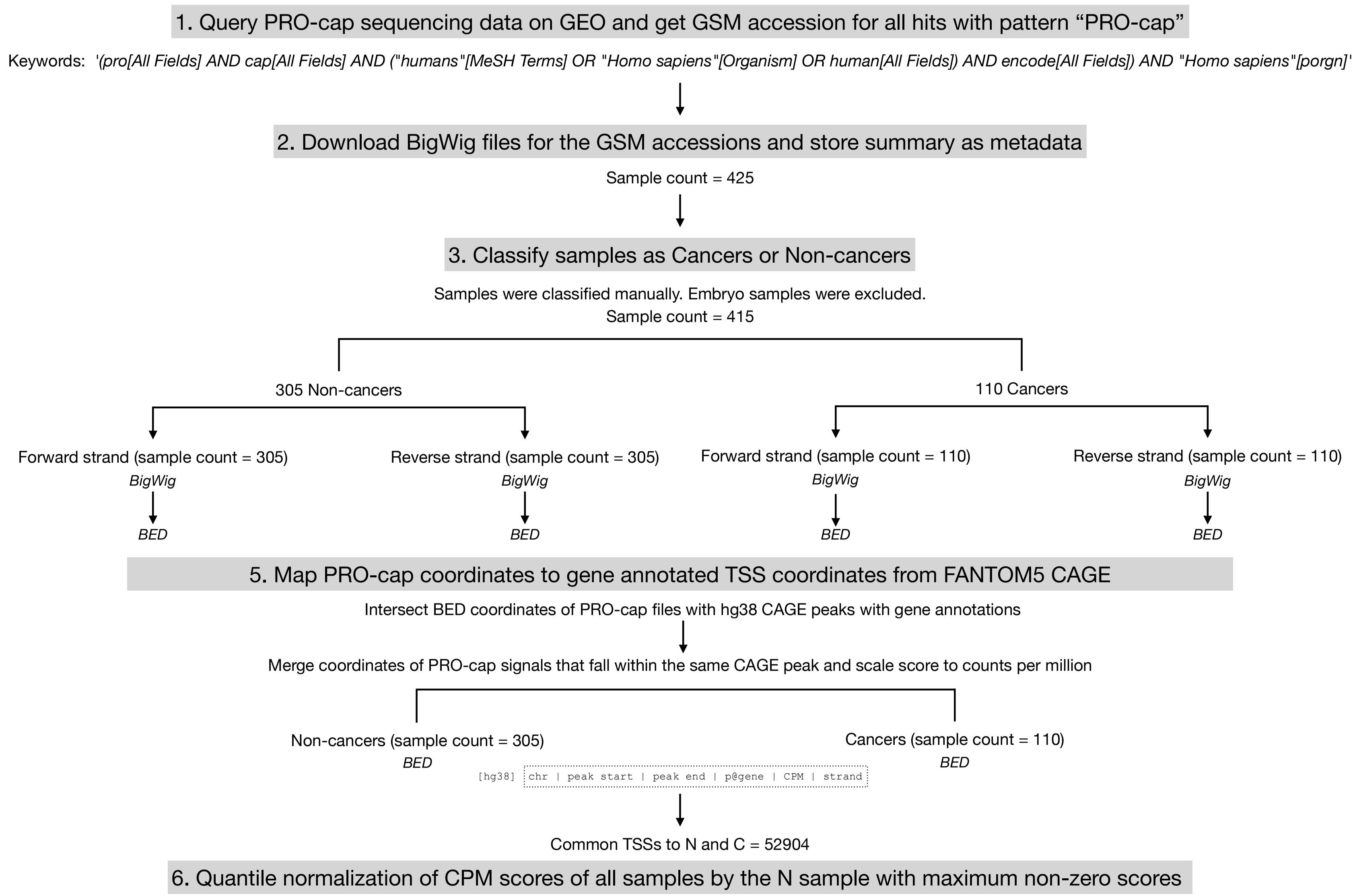

Schematic 5: Downloading, curating and classifying PRO-cap data.

13 Cost calculation

We derived the cost of transcription using TSS deployment and d based on earlier works that described the costs of transcribing a representative eukaryotic gene (as described by Lynch and Marinov, 2015). In adoption of the energy cost calculation framework, the gene–level parameters are replaced by d–level parameters (d length replaces the mRNA length, CAGE or PRO-cap signals replace mRNA count and the κ functions as the turnover rate) as described below.

13.1 Transcriptional cost of an unspliced population of d

This is the cost of transcribing the region between the transcription start site and the most upstream start codon without splicing the introns contained in d.

$$COST_{t,S} = TCI_{t,S}(46 \cdot d_t + 2.17 \cdot \kappa_S d_t)$$

Where,
$COST_{t,S}$ is the cost of transcribing the gene at the TSS t for the population S.
$TCI_{t,S}$ is the transcript count index for the TSS t in population S.
d_t is the length of d for the TSS t.
κ_S is the d–functionalization parameter for the population S.

13.2 Transcriptional $COST$ of spliced d

For a d that contains I introns, the cost is calculated as a combination of d_{exon} transcription and unitary splicings of I.

$$COST_{t,i,S} = TCI_{t,S}\left(46 \cdot d_{t,i,exon} + 2.17 \cdot \kappa_S d_t\right)$$

Where,
$COST_{t,i,S}$ is the cost of transcribing the gene at the TSS t spliced at intron i for the population S.
$TCI_{t,S}$ is the transcript count index for the TSS t in population S.
$d_{t,i,exon}$ is the length of the exons spliced at intron i for the TSS t.
d_t is the length d for the TSS t.
κ_S is the d–functionalization parameter for the population S.
i is an intron in the set of all introns I for d_t.

13.3 Gene–aggregated $COST$ of d

$$COST_{g,S} = \sum_{t}^{\tau} COST_{t,S}$$

Where,
$COST_{g,S}$ is the cost of transcribing the gene g for the population S.
$COST_{t,S}$ is the cost of transcribing the unspliced d for TSS t for the population S.
t is the TSS in gene–space for gene g.
τ is the set of all TSSs of the gene–space for gene g.
S is the C or N sample set.

13.4 Gene–aggregated $COST$ of spliced d

$$COST_{g,S} = \sum_{t}^{\tau} \sum_{i}^{I} COST_{t,i,S}$$

Where,
$COST_{g,S}$ is the cost of transcribing the gene g for the population S.
$COST_{t,i,S}$ is the cost of transcribing the d for TSS t spliced at intron i for the population S.
t is the TSS in gene–space for gene g.
τ is the set of all TSSs of the gene–space for gene g.
i is an intron in the set of all introns I for d_t.
S is the *C* or *N* sample set.

14 $COST$ Aggregate of Promoterome

The $COST$ aggregated across the entire promoterome of a sample including each splicing event within the d unitarily as well as the unspliced d.

$$CAP_s = \sum_{t}^{\tau} \sum_{i}^{I} COST_{t,i,s}$$

Where,
CAP_s is the $COST$ of aggregated promoterome for the sample s.
$COST_{t,i,S}$ is the cost of transcribing the d for TSS t spliced at intron i for the population S.
t is the TSS in gene–space for gene g.
τ is the set of all TSSs of the gene–space for gene g.
i is an intron in the set of all introns I for d_t.
S is the *C* or *N* sample set.
